# scMAPA: Identification of cell-type–specific alternative polyadenylation in complex tissues

**DOI:** 10.1093/gigascience/giac033

**Published:** 2022-04-30

**Authors:** Yulong Bai, Yidi Qin, Zhenjiang Fan, Robert M Morrison, KyongNyon Nam, Hassane M Zarour, Radosveta Koldamova, Quasar Saleem Padiath, Soyeon Kim, Hyun Jung Park

**Affiliations:** Department of Human Genetics, Graduate School of Public Health, University of Pittsburgh, Pittsburgh, PA 15261, USA; Department of Human Genetics, Graduate School of Public Health, University of Pittsburgh, Pittsburgh, PA 15261, USA; Department of Computer Science, School of Computing and Information, University of Pittsburgh, Pittsburgh, PA 15213, USA; Department of Medicine and Division of Hematology/Oncology, University of Pittsburgh, School of Medicine, Pittsburgh, PA 15213, USA; Department of Immunology, University of Pittsburgh, School of Medicine, Pittsburgh, PA 15213, USA; Department of Computational and Systems Biology, University of Pittsburgh Medical Center, Pittsburgh, PA 15213, USA; Department of Environmental and Occupational Health, Graduate School of Public Health, University of Pittsburgh, Pittsburgh, PA 15261, USA; Department of Medicine and Division of Hematology/Oncology, University of Pittsburgh, School of Medicine, Pittsburgh, PA 15213, USA; Department of Immunology, University of Pittsburgh, School of Medicine, Pittsburgh, PA 15213, USA; Department of Environmental and Occupational Health, Graduate School of Public Health, University of Pittsburgh, Pittsburgh, PA 15261, USA; Department of Human Genetics, Graduate School of Public Health, University of Pittsburgh, Pittsburgh, PA 15261, USA; Department of Neurobiology, School of Medicine, University of Pittsburgh, Pittsburgh, PA 15213, USA; Department of Pediatrics, School of Medicine, University of Pittsburgh, Pittsburgh, PA, 15224, USA; Division of Pediatric Pulmonary Medicine, UPMC Children’s Hospital of Pittsburgh, Pittsburgh, PA 15224, USA; Department of Human Genetics, Graduate School of Public Health, University of Pittsburgh, Pittsburgh, PA 15261, USA

**Keywords:** post-transcriptional regulation, alternative polyadenylation, single-cell RNA, cell-type–specific regulation, confounding factors

## Abstract

**Background:**

Alternative polyadenylation (APA) causes shortening or lengthening of the 3ʹ-untranslated region (3ʹ-UTR) of genes (APA genes) in diverse cellular processes such as cell proliferation and differentiation. To identify cell-type–specific APA genes in scRNA-Seq data, current bioinformatic methods have several limitations. First, they assume certain read coverage shapes in the scRNA-Seq data, which can be violated in multiple APA genes. Second, their identification is limited between 2 cell types and not directly applicable to the data of multiple cell types. Third, they do not control undesired source of variance, which potentially introduces noise to the cell-type–specific identification of APA genes.

**Findings:**

We developed a combination of a computational change-point algorithm and a statistical model, single-cell Multi-group identification of APA (scMAPA). To avoid the assumptions on the read coverage shape, scMAPA formulates a change-point problem after transforming the 3ʹ biased scRNA-Seq data to represent the full-length 3ʹ-UTR signal. To identify cell-type–specific APA genes while adjusting for undesired source of variation, scMAPA models APA isoforms in consideration of the cell types and the undesired source. In our novel simulation data and data from human peripheral blood mononuclear cells, scMAPA outperforms existing methods in sensitivity, robustness, and stability. In mouse brain data consisting of multiple cell types sampled from multiple regions, scMAPA identifies cell-type–specific APA genes, elucidating novel roles of APA for dividing immune cells and differentiated neuron cells and in multiple brain disorders.

**Conclusions:**

scMAPA elucidates the cell-type–specific function of APA events and sheds novel insights into the functional roles of APA events in complex tissues.

## Background

Many mammalian messenger RNAs contain multiple polyadenylation (pA) sites, e.g., proximal and distal, in their 3ʹ-untranslated region (3ʹ-UTR) [1, 2]. Using multiple pA sites in each gene, alternative polyadenylation (APA) post-transcriptionally produces multiple APA isoforms with various 3ʹ-UTR lengths. These APA events are involved in diverse cellular processes such as cell proliferation and differentiation in particular cell types. For example, cancer cells of diverse types are reported to undergo widespread 3ʹ-UTR shortening events [3], whereas senescent cells tend to show widespread 3ʹ-UTR lengthening events [4]. To identify such APA genes for each cell type (cell-type–specific APA genes) in complex tissues, developing a computational method that accurately analyzes single-cell RNA sequencing (scRNA-Seq) data is essential because the data present the cell-type–specific transcriptome.

**Figure 1:**
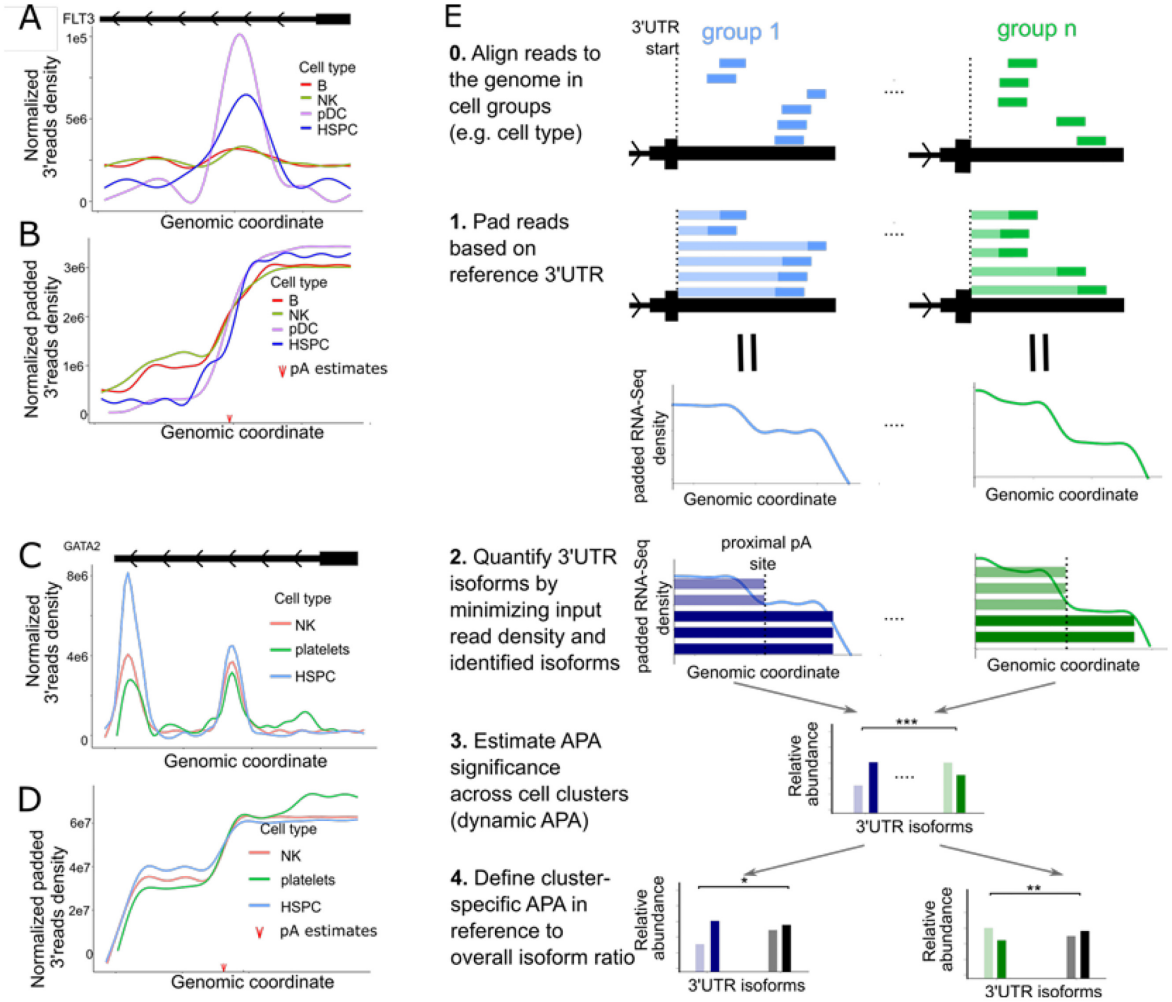
Motivation and schematic illustration of scMAPA. (A) The read density shape on the FLT3 3ʹ-UTR in multiple cell types of 10k PBMC scRNA-Seq data. (B) The transformed read density shape on the FLT3 3ʹ-UTR in multiple cell types of 10k PBMC scRNA-Seq data. The red arrow indicates the proximal polyA site predicted. (C) The read density shape on the *GATA2* 3ʹ-UTR in multiple cell types of 10k PBMC scRNA-Seq data. (D) The transformed read density shape on the *GATA2* 3ʹ-UTR in multiple cell types of 10k PBMC scRNA-Seq data. The red arrow indicates the proximal polyA site predicted. (E) In Steps 0 and 1, bars in solid color represent 3ʹ-biased scRNA-Seq reads and bars in light color indicate how the 3ʹ-biased reads are padded from the 3ʹ start site to the end of the read to represent the full-length 3ʹ-UTR of the transcript. In Step 2, the blue and green bars indicate the estimated isoforms in each cell type, where solid and light coloring mode indicate 3ʹ UTR long and short isoforms. In Steps 3 and 4, the bars represent the estimated number of APA isoforms in each cell type. HSPC: hematopoietic stem and progenitor cell; NK: natural killer; pDC: plasmacytoid dendritic cell.

To identify cell-type–specific APA genes in scRNA-Seq data, several bioinformatic methods have been developed, such as scDAPA [5], Sierra [6], and scAPA [7]. Although they have various strengths, they also have several limitations when used for complex tissue data. First, they only consider certain read coverage shapes in the input scRNA-Seq data to estimate APA events. This is because several scRNA-Seq techniques generate the 3ʹ-enriched reads and the accumulation of the reads that originate from the same APA isoform forms a peak. To discriminate the signal part of the peak from noise, the existing methods assume certain signal shapes in their peak calling. For example, scAPA uses the findPeaks module in the Homer package [8] with the preset peak size and height. However, these assumptions can be violated in multiple genes across multiple cell types. For example, it would be useful to quantify APA isoforms of *FLT3* and *GATA2*in the scRNA-Seq data on peripheral blood mononuclear cells (PBMCs) of a healthy donor (10k in https://www.10xgenomics.com/) because their abnormality may lead to blood disorders [9, 10]. However, their 3ʹ tags form peaks with different sizes and heights across various cell types (Fig. 1A and C) for which the existing methods would not be able to identify peaks from some of the cell types. Second, the existing methods cannot identify cell-type–specific APA genes when the scRNA-Seq data contain >2 cell types, which is typical for complex tissues. scDAPA and Sierra can only compare cell types in a pairwise fashion, which limits their ability for global comparison when >2 cell types exist. While scAPA is the only method to identify APA genes for multiple cell types, it identifies genes in which the APA isoform ratio (the ratio of long and short 3ʹ-UTR isoforms) varies across the cell types and does not further identify which specific cell types drive this variation. Third, the existing methods do not adjust for other factors that affect the scRNA-Seq data across cell types. For example, when the scRNA-Seq data are sampled from various brain regions, some cell types reside in multiple brain regions [11]. Then, molecular dynamics specific to the brain regions would affect different portions of the residing cell types, introducing noise to the cell-type–specific identification of APA genes. Thus, to identify cell-type–specific APA genes, one may need to adjust for the brain region information. Fourth, there is no simulation platform to compare statistical power and specificity of the methods identifying APA genes in scRNA-Seq data. Although such a platform is necessary to evaluate the methods with the ground truth, it has been challenging to simulate APA and non-APA genes because it is not clear how the read coverage shapes differ between APA and non-APA genes.

To address these limitations, we developed a combination of a computational optimization algorithm and a statistical model, single-cell Multi-group identification of APA (scMAPA). To address the first limitation and quantify APA isoforms without assumptions on the read coverage shape, scMAPA first transforms the input scRNA-Seq data and then formulates a change-point detection problem on the transformed data. First, scMAPA transforms the 3ʹ-enriched signal of scRNA-Seq data to represent the full-length 3ʹ-UTR signal. For *FLT3* and *GATA2* in the PBMCs of a healthy donor, this transformation makes the APA short and long isoforms readily distinguishable across all cell types regardless of the differences in read coverage shape (Fig. 1B and D). Then, on the transformed coverage shapes, scMAPA quantifies APA isoforms by detecting a change-point. To address the second and the third limitations to identify cell-type–specific APA genes while controlling undesired source of variation, scMAPA considers cell type information and the undesired source by developing a statistical model with them as covariates. To address the fourth limitation and simulate APA genes, we identified a common feature of APA genes in real data, a high variance in the APA isoform ratios across cell types, and simulate the APA isoform specific count matrix on the basis of the common feature. Because this simulation platform does not generate data at the level of read coverage shape, it can generate the ground truth APA genes without having to resolve the difference between APA and non-APA genes in the read coverage shape. By systematically addressing these limitations, scMAPA accurately and robustly identifies cell-type–specific APA genes and facilitates a systematic understanding of APA regulation in complex tissues in this article.

## Findings

### scMAPA

To identify cell-type–specific APA genes accurately and robustly, scMAPA combines a computational algorithm and a statistical model in 3 steps. First, scMAPA transforms each read in the scRNA-Seq data by padding it from the annotated 3ʹ-UTR start site to where the read ends (Step 1 in Fig. 1E). While the scRNA-Seq reads are usually 3ʹ biased owing to the 3ʹ selection and enrichment techniques in the library construction step, the transformed reads will represent the read coverage shape across the 3ʹ-UTRs. Second, scMAPA identifies a pA site that minimizes the difference between the expected coverage shape of the inferred APA isoforms and the accumulated observed coverage (change-point, Step 2 in Fig. 1E). Because the difference can be calculated by a quadratic function, scMAPA detects the change-point by quadratic programming [12]. To solve this problem for multiple cell types in scRNA-Seq data, scMAPA extends multiple modules of DaPars2 [13], which uses the quadratic programming approach to identify APA genes in bulk RNA-Seq data. Third, to simultaneously identify APA genes across cell types and for each cell type based on the APA isoforms quantified, scMAPA develops a multinomial regression model that explicitly models each APA isoform (Step 3 in Fig. 1E) with covariates representing the cell type and other source of variation (Step 4 in Fig. 1E). On the model, scMAPA uses the log-likelihood test and the Wald test to identify across–cell-type APA genes and cell-type–specific APA genes, respectively. Altogether, scMAPA is the first method to simultaneously identify across–cell-type and cell-type–specific APA genes in scRNA-Seq data of multiple cell types.

### scMAPA outperforms the other method in sensitivity for the multi-group setting

**Figure 2:**
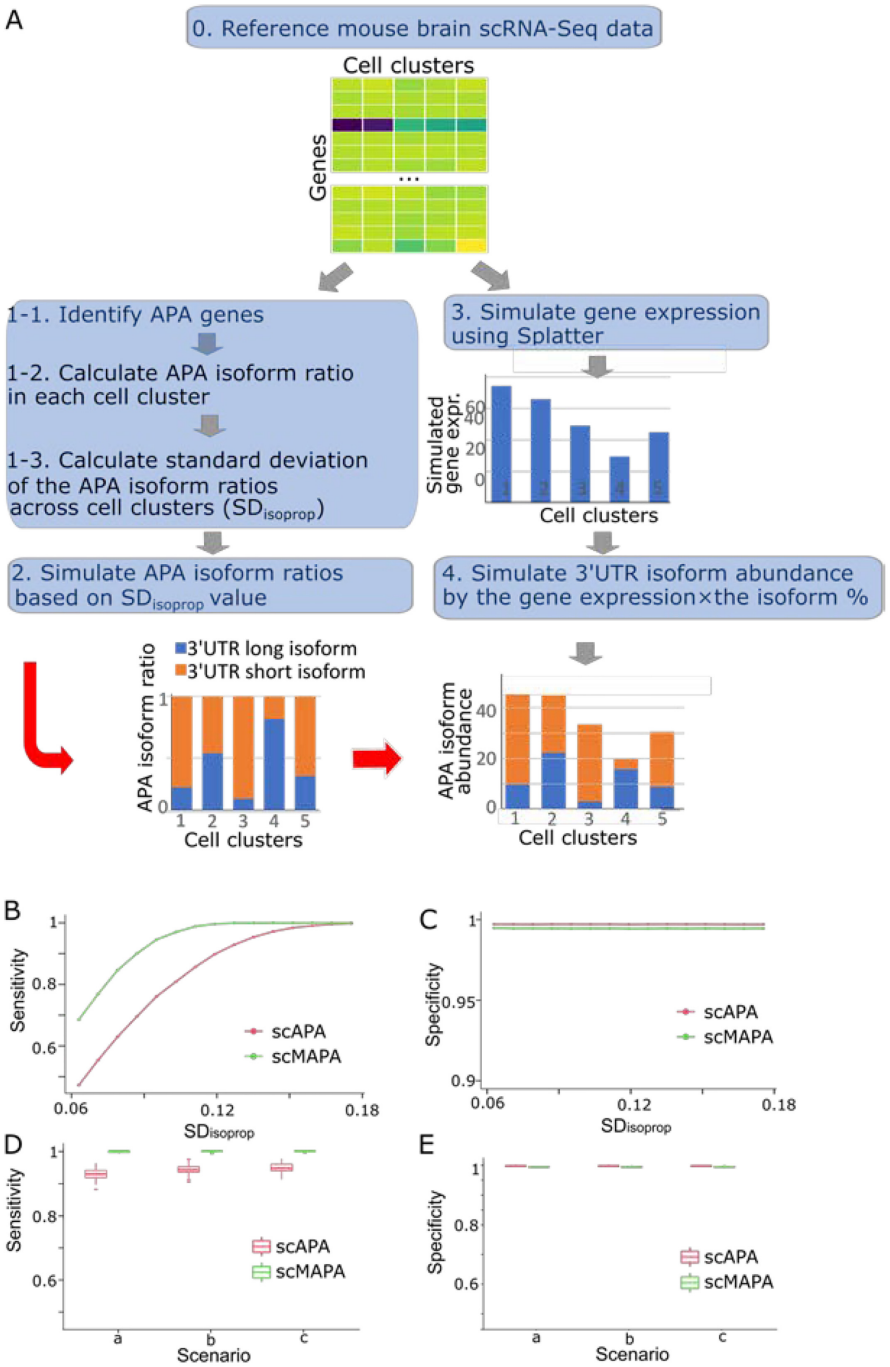
Performance assessment on the statistical component of scMAPA and scAPA using simulated data, with fixed number of true APA events (500 of 5,000) and uniform distribution of cell cluster size (600 cells in each cell type) (A). Illustration of the simulation process. Genes identified as significant APA genes by both scMAPA and scAPA were considered as APA genes. Genes identified as non-significant APA genes by both methods were considered as non-APA genes. (B), (C) Comparison of scMAPA vs scAPA in terms of sensitivity and specificity. We varied the standard deviation (SD) of APA isoforms across clusters (SD_isoprop_) for 500 true APA genes (0.06–0.18) with the fixed ${\mathrm{S}}{{\mathrm{D}}_{{\mathrm{isoprop}}}}$ value for 4,500 non-APA genes (0.009). (D), (E) Comparison of scMAPA vs scAPA in terms of sensitivity and specificity. We varied cell cluster size: (20%, 20%, 20%, 20%, 20%) for scenario a, (30%, 17.5%, 17.5%, 17.5%, 17.5%) for b, and (50%, 12.5%, 12.5%, 12.5%, 12.5%) for c. In the box-and-whisker plot, the lower end of the line indicates the minimum excluding outlier, the bottom line first quartile, the middle line median, the upper line third quartile, the upper end of the line maximum excluding outlier.

To assess the performance of scMAPA using the ground truth, we developed a novel simulation platform where APA isoform-specific expressions are simulated in multiple steps. First, to learn parameters from real data, we determined APA genes across 5 cell types of mouse brain scRNA-Seq data [11] (neurons, astrocytes, immune cells, oligodendrocytes, and vascular cells, Step 0 in Fig. 2A) as those identified by both scAPA and scMAPA. We used only scAPA and scMAPA because they are the only methods designed for >2 cell types. Then, we quantified a common feature of the APA genes by calculating the proportion of the long and short isoforms in each cell type and the standard deviation of the proportions across the 5 cell types (SD_isoprop_, see Methods). To validate the effectiveness of this measure for APA simulation, we calculated SD_isoprop_ values for non-APA genes that scAPA and scMAPA agreed on in the data. We found that SD_isoprop_ values significantly distinguish APA genes from non-APA genes (0.127 vs 0.009 of SD_isoprop_ on average, *P* < 10^–16^, Supplementary Fig. S2A), suggesting that it is reasonable to simulate APA genes to have high SD_isoprop_ values in the data of multiple (≥2) cell types (multi-group setting).

To simulate APA long and short isoform expressions, we simulated gene expression values for 5 simulated cell clusters (Step 3 in Fig. 2A) and divided the values into APA long and short isoforms based on the SD_isoprop_ values. Because the SD_isoprop_ values are the standard deviation of APA long and short isoform ratios, the simulation based on the high SD_isoprop_ values estimated from the APA genes spreads the APA long and short isoform expressions across the 5 cell clusters. In the same sense, the simulation based on the low SD_isoprop_ values produces less variable isoform expressions across the clusters. On the simulated APA isoform expressions for APA and non-APA genes, we ran scMAPA and scAPA to assess their sensitivity and specificity. In the first scenario simulating 500 APA and 4,500 non-APA genes, we varied SD_isoprop_ values for APA genes in the range observed in the mouse brain data (0.06–0.18, Supplementary Fig. S2A). Across all simulated SD_isoprop_ values, scMAPA consistently outperforms scAPA with higher sensitivity (Fig. 2B) while having a similar specificity (Fig. 2C). In assessing specificity, we did not vary SD_isoprop_ values for non-APA genes because the mouse brain data show a narrow range of SD_isoprop_ values for non-APA genes (Supplementary Fig. S2A). In the second scenario, we varied the number of APA and non-APA genes and the cell group size while fixing the SD_isoprop_ values for APA and non-APA genes (to 0.127 and 0.009, respectively). With various numbers of true APA genes (250, 500, and 1,000), scMAPA consistently outperforms scAPA in terms of sensitivity (Fig. 2D and Supplementary Fig. S2B and D) with a slight loss of specificity (Fig. 2E and Supplementary Fig. S2C, E, F). To sum, scMAPA outperforms scAPA in various simulation scenarios in terms of sensitivity with a similar level of specificity.

### scMAPA outperforms existing methods in identifying APA isoforms with high robustness

**Figure 3:**
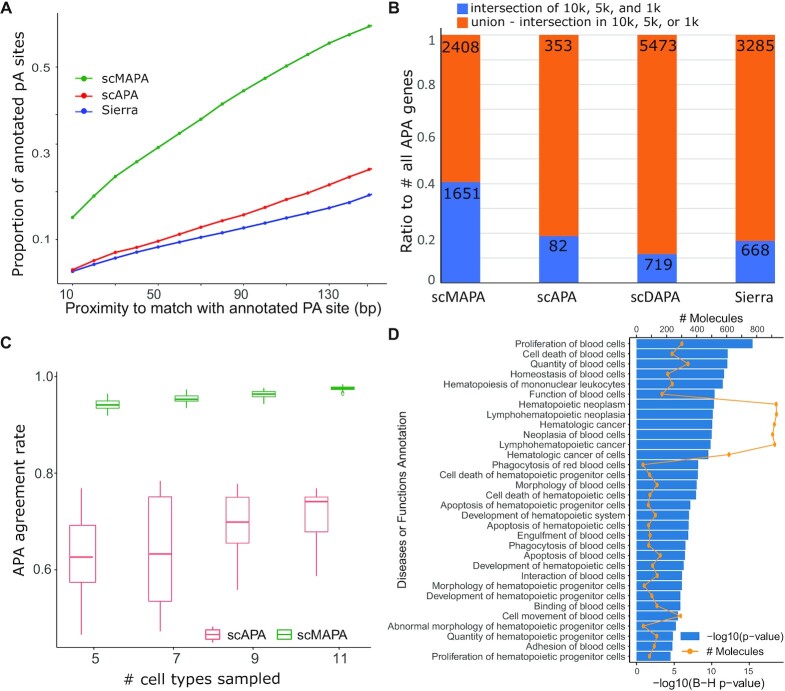
Performance assessment of scMAPA, scAPA, scDAPA, and Sierra using PBMC data. (A) The ratio of annotated pA sites identified by scMAPA vs scAPA and Sierra on the PBMC 10k data. The identified pA sites were deemed annotated when they are within a range to any annotated pA sites while the range was set from 10 to 130 bp, respectively. We extracted the annotated pA sites from PolyASite 2.0. (B) The ratio of significant APA genes found in all 3 PBMC data (10k, 5k, and 1k) in blue bar and in any combination but all 3 in orange by scMAPA, scAPA, scDAPA, and Sierra. (C) Box plots showing the proportion of the overlap between sample APA genes and total APA genes normalized to total APA genes (APA agreement ratio). The APA agreement ratio values were evaluated in various numbers of cell types sampled. (D) Significance of enrichment (blue bar) and number of overlaps (orange line) of 3,574 scMAPA APA genes on IPA Disease and Function terms with the keyword “blood” or “hematology” In the box-and-whisker plot, the lower end of the line indicates the minimum excluding outlier, the bottom line first quartile, the middle line median, the upper line third quartile, the upper end of the line maximum excluding outlier.

To assess the performance of scMAPA using real data, we used 3 PBMC data sets of various numbers of cells (1k, 5k, and 10k data representing the number of cells) from the 10x Genomics website (see Methods, Supplementary Table S1). To assess the accuracy of scMAPA in identifying annotated pA sites, we identified pA sites in the 10k and 5k data using scMAPA, scAPA, and Sierra. scDAPA was not included in this comparison because it does not return results that are compatible for the comparison, such as pA peaks, sites, or intervals. Among the identified pA sites, we calculated the proportion that are close to the annotated pA sites in PolyASite 2.0 [14] (see Methods). scMAPA consistently outperforms the other methods by identifying the highest proportion of the annotated pA sites across all degrees of proximity (Fig. 3A, Supplementary Fig. S3A and B). This result suggests the outperformance of scMAPA in identifying possible bona fide APA events originating from the annotated pA sites.

We further evaluated the robustness of the methods in 2 ways. First, we ran scMAPA, scAPA, scDAPA, and Sierra to identify APA genes in the 1k, 5k, and 10k PBMC data. Because the 1k, 5k, and 10k data sets comprise similar sets of cell types from healthy adults (1k and 10k from the same donor and 5k from another healthy donor, Supplementary Table S1), the APA genes are expected to overlap across the data sets. Thus, a high percentage of APA genes identified commonly across the data sets would indicate the robustness of the methods to the number of cells in the data. Although Sierra and scDAPA cannot identify APA genes directly from multiple (>2) cell types, we artificially identified the APA genes for multiple cell types by combining all pairwise identifications after false-discovery rate (FDR) control (see Methods). Compared to the competing methods, scMAPA identifies a 2-fold higher percentage of APA genes commonly across the 3 types of the data sets (40.7% vs 18.9%, 11.6%, and 18.6%, respectively, Fig. 3B), showing that scMAPA identifies APA genes robustly to the number of cells in the data. Second, from the 10k data comprising the total of 13 cell types, we randomly sampled various numbers of cell types (5, 7, 9, and 11) and ran scMAPA and scAPA separately in each sample. For direct comparison, we compared scMAPA only with scAPA, the only other method that can directly handle the multi-group setting. In the APA genes identified in each sample (sample APA genes), we calculated the overlap with those identified using all 13 cell types (total APA genes). Then, we calculated APA agreement ratio, defined as the number of the overlap between the sample and total APA genes normalized by the number of total APA genes. In all the numbers of cell types sampled, scMAPA outperforms scAPA with higher APA agreement ratios (Fig. 3C). Because the APA agreement ratio indicates the number of the total APA genes that are found in the sample APA genes, the result shows that scMAPA identifies APA genes robustly to the number of cell types in the data.

Furthermore, to investigate whether the APA genes identified by scMAPA are biologically relevant, we performed Ingenuity Pathway Analysis (IPA) on 3,574 APA genes that scMAPA identified in the 10k PBMC data. Especially, to accurately investigate the APA genes’ roles in PBMC biology, we set the 18,804 genes expressed in the data as the background (see Methods). This IPA analysis shows significant (Benjamini-Hochberg [B-H] *P* < 0.01) enrichments to 32 IPA terms that are characterized with keywords “blood” and “hematology” (Fig. 3D), suggesting that the APA genes identified by scMAPA can play important roles in PBMC biology.

To examine the unique contribution of scMAPA in characterizing the function of APA genes for PBMC biology, we manually inspected 1,432 APA genes that are identified only by scMAPA, not by other methods (scMAPA-unique APA genes, Supplementary Table S2). In the scMAPA-unique APA genes, we found clear changes in the APA isoform ratios across the cell types and great potential to function for PBMC biology. For example, *FLT3* and *GATA2*are included in the scMAPA-unique APA genes and show the dynamic APA isoform ratios across the cell types especially after the data transformation step of scMAPA (Fig. 1B and D). Interestingly, *GATA2*is an APA gene in the scRNA-Seq data of bone marrow mononuclear cells from patients with acute myeloid leukemia [15]. Because hematopoietic stem and progenitor cells (HSPC in Fig. 1D) originate from bone marrow [16], we speculate that the molecular mechanisms rendering the APA event on *GATA2* in the bone marrow mononuclear cells cause *GATA2* to show different APA patterns than other cells in the PBMC. Together, scMAPA enables accurate and robust identification of biologically relevant APA genes in the PBMC scRNA-Seq data.

### scMAPA estimates APA effect size and identifies APA genes across multiple cell types

**Figure 4:**
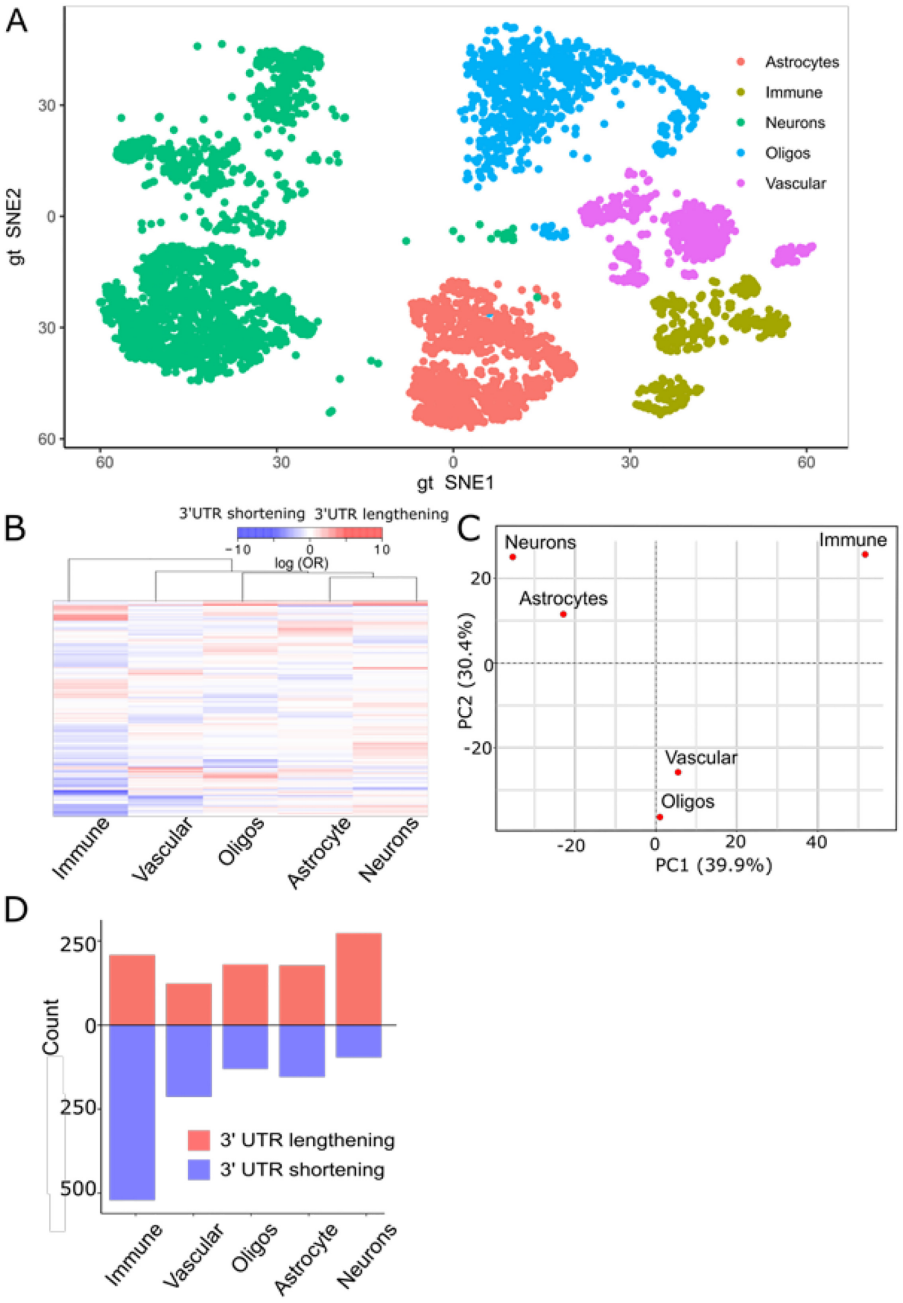
A novel module of scMAPA cell-type–specific APA identification on the mouse brain data. (A) tSNE plot showing the cell types of the mouse brain scRNA-Seq data. (B) Heat map of the APA effect sizes estimated for each cell type, representing the coefficients in the scMAPA logistic regression model. (C) PCA plot showing how the cell types are similar or dissimilar in the APA effect size. PC1 and PC2 together account for 70.3% of the variation. (D) Bar plot showing the counts of significant 3ʹ-UTR lengthening (red) and shortening (blue) identified in each cell type.

Compared with other methods, scMAPA is the only method that can estimate the effect size and the significance of APA events for each cell type in the multi-group setting (see Methods). To demonstrate how the APA effect size enables us to understand the post-transcriptional regulation in each cell type, we analyzed the mouse brain scRNA-Seq data comprising 5 major cell types: neurons, astrocytes, immune cells, oligodendrocytes, and vascular cells [11] (Fig. 4A, see Methods). First, to identify the distances among the cell types in terms of the APA effect size, scMAPA estimated the effect size of 3,223 genes significantly (B-H *P* < 0.05) identified as APA genes across the 5 cell types (Fig. 4B). Based on these effect sizes, we performed principal component analysis (PCA) (Fig. 4C) and calculated Euclidean distance (Supplementary Fig. S4A) between the cell types. While both analyses support the previous finding that immune cells and neurons are most different in terms of the APA effect size [7], they further reveal that immune cells are most different from all the other cell types. Second, to identify the overall relationships between APA regulation and gene expression regulation, we correlated the APA effect sizes of all the identified genes with their expression level. The result shows that the APA effect sizes are not correlated with their expression level in all the cell types (e.g., Spearman ρ < 0.05 for all cell types, Supplementary Fig. S4D–H), demonstrating that APA events are regulated independently of gene expression in the mouse brain.

Furthermore, cell-type–specific APA genes (3ʹ-UTR shortening and lengthening genes) identified by scMAPA provide a systematic understanding of cellular status. Previous studies showed that APA is involved in regulating cell division status. For example, various types of dividing cells are associated with widespread 3ʹ-UTR shortening [17, 18]. Likewise, differentiated and senescent cells are associated with widespread 3ʹ-UTR lengthening [19, 20]. To systematically extend these findings that were made in cell line data [17, 19, 21] or heterogeneous tissue data [18], we ran scMAPA in the mouse data further to identify 438 significant (B-H *P* < 0.05) cell-type–specific APA genes in neurons, 891 in immune cells, 374 in astrocytes, 422 in vascular cells, and 430 in oligodendrocytes, with some overlaps across the cell types (Supplementary Fig. S4B). A further division into 3ʹ-UTR shortening and lengthening genes in each cell type (Fig. 4D) showed that 3ʹ-UTR shortening and lengthening are significantly enriched in immune cells and neurons, respectively. As immune cells actively divide to dynamically regulate the immune system, the enriched 3ʹ-UTR shortening may contribute to active division. In the same sense, we could find a biological explanation for why 3ʹ-UTR lengthening is enriched in neurons. While neurons do not divide once they are formed in the brain, our result suggests that 3ʹ-UTR lengthening can play a significant role in keeping neurons from further dividing. Together, by identifying cell-type–specific APA genes, scMAPA systematically links the cellular APA profile to dividing immune cells and differentiated neurons.

### scMAPA adjusts for undesired source of variance to uncover APA functions that would be invisible without the adjustment

**Figure 5:**
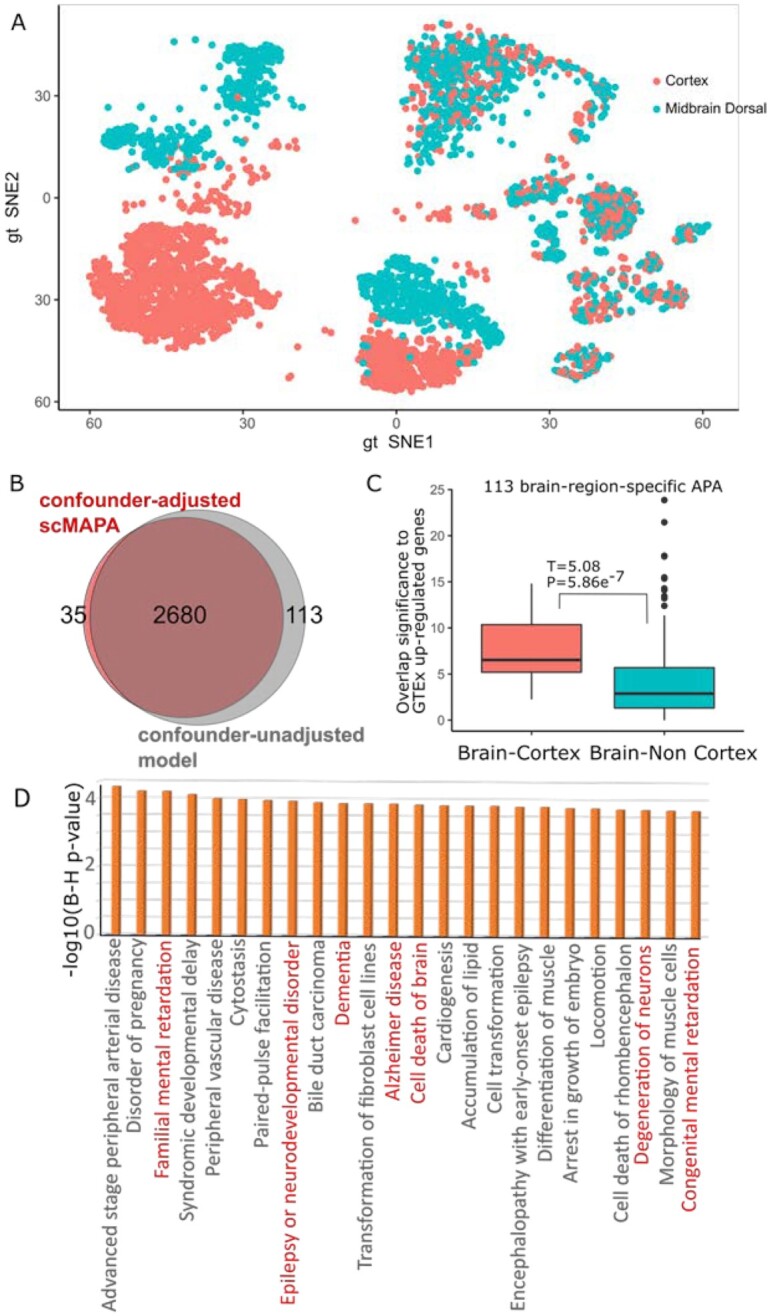
(A) tSNE plot showing the brain region of the mouse brain scRNA-Seq data. (B) Venn diagram showing the APA genes identified by the confounder-adjusted scMAPA and the confounder-unadjusted model. (C) Box plot showing significance of overlap between the 113 genes and the up-regulated genes in GTEx brain samples whether they are from cortex (red) or not (green). (D) Significance (B-H *P*-value) of IPA enrichment terms that are uniquely and significantly (B-H *P* < 10^–2^) enriched to 2,793 confounder-adjusted scMAPA. In the box-and-whisker plot, the lower end of the line indicates the minimum excluding outlier, the bottom line first quartile, the middle line median, the upper line third quartile, the upper end of the line maximum excluding outlier.

To show how scMAPA controls undesired source of variance in the data and why it is important, we analyzed the mouse brain data consisting of 5 cell types collected from 2 brain regions (cortex and midbrain). Because some cell types were collected from multiple brain regions (Figs 4A and 5A), APA genes associated with a brain region could be mistakenly identified as cell-type–specific APA genes, which would further confuse the study of cell-type–specific functions of APA genes. To see whether scMAPA can remove such false-positive APA genes, we set scMAPA to adjust for the brain region information (cortex and midbrain dorsal) (brain-region–adjusted scMAPA). Then, we compared the result from another scMAPA run that does not adjust for that information (brain-region–unadjusted model), separately. As the brain-region–adjusted scMAPA and the brain-region–unadjusted model identified 2,715 and 2,793 APA genes, respectively (Supplementary Table S6), 113 genes are not identified in the brain-region–adjusted scMAPA. Thus, these APA genes are expected to be related to the brain region from which it was sampled (cortex and midbrain) (Fig. 5B). To test whether the 113 genes function specifically for the brain region, we tested whether they express highly specifically in the brain region. To conduct this test comprehensively, we identified their human homolog genes in the Mouse Genomic Informatics (MGI) homology database and compared expression of the human homologs between cortex and other brain regions in the Genotype-Tissue Expression (GTEx) [22] (see Methods). The result shows that these APA genes are significantly up-regulated in brain cortex compared to other brain regions (*P* = 5.8 × 10^–7^, Fig. 5C, Supplementary Table S7), suggesting that their functions are specific to brain cortex. Because GTEx did not collect the expression data for midbrain, we did not conduct this analysis for midbrain. This result suggests that scMAPA can successfully adjust for undesired source of variation and identify APA genes likely caused by differences between cell types.

To demonstrate why adjusting for undesired source of variation is critical for accurate downstream analysis, we further conducted IPA analysis on the 2,715 and 2,793 APA genes identified by the brain-region–adjusted scMAPA and the brain-region–unadjusted model, respectively (brain-region–adjusted and brain-region–unadjusted APA genes, respectively). Comparing the IPA enrichment between brain-region–adjusted and brain-region–unadjusted APA genes, we found considerable differences in important terms for brain study: among the 24 terms to which the brain-region–adjusted APA genes are uniquely and significantly (B-H *P* < 0.01) enriched, 7 terms are directly related to brain diseases (Fig. 5D). For example, 2 terms with the keyword “mental retardation” are significantly enriched (B-H *P* < 2.2 × 10^–4^) only for the brain-region–adjusted APA genes. On the other hand, among the 30 terms to which the brain-region–unadjusted APA genes are uniquely and significantly enriched, no term refers to a brain disease (Supplementary Fig. S5A). With APA events potentially playing roles in brain diseases [23, 24], this result suggests that adjusting for the variation from brain region uncovers the APA genes that can play critical roles in brain disease, which would be invisible without the adjustment.

### Supplementary material

#### APA regulation on expression

Previous studies have suggested that APA genes are more likely differentially expressed [1, 2] because either 3ʹ-UTR shortening removes microRNA (miRNA) binding sites on the 3ʹ-UTR and evades miRNA-mediated repression or 3ʹ-UTR lengthening adds miRNA binding sites and enhances miRNA-mediated repression. Our analysis reaffirms the previous observations in the scRNA-Seq data.

#### scMAPA consensus with other methods

In the PBMC data, scMAPA results still recover most of the results from the other methods. To assess the overlap, we identified significant APA genes across all the cell types in scMAPA and scAPA. Because scDAPA and Sierra identify APA genes only between cell-type pairs, we combined the pairwise significant APA genes in each method separately. After controlling FDR on the combined APA genes, we called APA genes if they are significant in any of the pairwise identifications. While scMAPA identifies an intermediate number of APA genes between scDAPA and Sierra/scAPA (10k in Supplementary Fig. S3C and 5k in Supplementary Fig. S3D), more than half of scMAPA's findings are found in other methods (59.9% for 10k and 51.9% for 5k). While scMAPA solves an optimization problem based on the padding of 3ʹ-biased reads (Step 1 in Fig. 1C), it successfully recovers most results from other methods, validating the use of scMAPA for comprehensive identification.

#### Cell-type–specific APA genes in 10k PBMC data

The global size differences in PBMC cells are different from in the mouse brain data in several aspects. First, 3ʹ-UTR lengthening occurs more than 3ʹ-UTR shortening in all the cell types (Supplementary Fig. S4E). Second, however, the number of 3ʹ-UTR shortening genes is significantly correlated with that of lengthening genes across the cell types (*P* = 5 × 10^–5^, Supplementary Fig. S4F). Because both trends are not shown in the mouse brain data, scMAPA elucidates the unique APA profiles of the PBMC data.

#### Specificity of high expression in 113 brain-region–related APA genes for the brain cortex region

In demonstrating the high expression of the 113 brain-region*–*related APA genes in the brain cortex region, we further investigated whether the APA genes are not down-regulated in neither brain vs non-brain samples (Supplementary Fig. S5B) nor cortex vs non-cortex brain samples (Supplementary Fig. S5C). Also, this brain-region*–*specific expression pattern was not found for 2,715 APA genes identified by the brain-region*–*adjusted scMAPA (Supplementary Fig. S5D*–*G). Together with our analysis on up-regulation (Fig. 5), the results suggest that the 133 APA genes function specific to the brain region.

### Discussion

To identify APA genes in scRNA-seq data for complex tissue data, we developed scMAPA, which addresses several limitations in existing methods using a combination of a computational optimization algorithm and a statistical model. First, while existing methods detect APA signals with assumptions on the shape of the input data, scMAPA avoids such assumptions by formulating this task in quadratic programming. By solving this quadratic programming for genes with different read coverage shapes across cell types, scMAPA outperforms existing methods in accurately and robustly identifying APA genes in various simulated (Fig.   2) and PBMC data (Fig. 3). Second, scMAPA identifies APA genes specific to each cell type in a statistically rigorous model. These cell-type–specific APA genes elucidate their connections to the cell division status of immune cells and neurons in the mouse brain data (Fig. 4). Third, scMAPA can control confounding factors. In the mouse brain data of 5 cell types collected from 2 brain regions, scMAPA can distinguish the 113 APA genes that are likely related to the brain regions. By removing the false-positive APA genes from further analyses, scMAPA could clarify the functions of APA genes in brain diseases such as “mental retardation” (Fig. 5). Last, we developed a novel simulation platform in which to assess the statistical power of identification methods on the basis of a common feature of APA genes, the high variation of APA long and short isoforms (SD_isoprop_) across cell clusters.

When identifying the annotated pA sites, scMAPA makes point estimations of the pA sites. While other methods mainly produce interval estimates, point estimations are more directly relevant to further analyses than interval estimations, e.g., conducting omics data analyses and designing validation experiments. However, when point estimation methods are naively compared to interval estimation methods in terms of the distance to the annotated pA sites, point estimations produce generally disadvantageous results because point estimation returns a single point while interval estimation returns 2 points (start and end of the interval) to measure the distance. For example, the interval estimations produce better results than the point estimations within both Sierra and scAPA (Supplementary Fig. S3A and B). Even with this disadvantage of point estimation for comparison purposes, the point estimation of scMAPA outperforms the interval estimation results of Sierra and scAPA in identifying the annotated pA sites, showing a clear advantage of scMAPA (Fig. 3A, Supplementary Fig. S3A and B).

A limitation of this article is that, although scMAPA can consider >2 pA sites (see Methods), our analysis focused on the use of 2 pA sites (most distal and most proximal) for the following reasons. First, some of the methods that compare with scMAPA consider only 2 pA sites, e.g., scAPA. For fair comparisons, we limited scMAPA to consider 2 pA sites. Second, we focused on this binary APA trend to make it easier to investigate across multiple cell types. In the future, we plan to consider >2 pA sites in complex tissues after characterizing the binary trend across multiple cell types. For example, after solving the quadratic programming with >2 pA sites and developing a multinomial logistic regression model with the identified pA sites in the mouse brain data, we can estimate the APA effect size for each use of the multiple pA sites.

scMAPA can be extended in the following directions in the future. First, the transformation step of scMAPA allows us to use other methods originally developed for bulk RNA-Seq data (e.g., APATrap [25], TAPAS [26]) to analyze scRNA-Seq data. Because the methods can identify APA genes in the full-length 3ʹ-UTR signal of transcripts, scMAPA can use such methods on the transformed scRNA-Seq data that represent the full-length 3ʹ-UTR signal of transcripts. This extension can make those APA identification methods become reasonable alternatives because those methods are well established and studied in terms of sensitivity and specificity. Second, while existing methods developed for scRNA-Seq data are mostly designed for 3ʹ-biased scRNA-Seq data (e.g., 10x), scMAPA can be used for scRNA-Seq data that are not 3ʹ-biased (e.g., Smart-seq2 [27]) simply by skipping the data transformation step, because the scRNA-Seq data already present the full-length 3ʹ-UTRs.

Altogether, we developed scMAPA to identify APA genes in scRNA-Seq data of multiple cell types. With high sensitivity and robustness in addition to adjusting for undesired source of variations, scMAPA elucidates the cell-type–specific function of APA events, which is essential to shed novel insights into the functional roles of APA events in complex tissues.

## Methods

### Processing data sets

PBMC data. Aligned BAM files were downloaded from the 10X genomics repository (https://support.10xgenomics.com/single-cell-gene-expression/datasets). According to the data description of 10X, 1k and 10k data were generated from the same materials. The 5k data were generated from different cells. PCR duplicates were removed using UMI-tools 1.0.0 with “–method = unique –extract-umi-method = tag –umi-tag = UB –cell-tag = CB.” Cell clustering was performed using R package Seurat 3.1.4 [28]. To further validate the number of clusters, we examined the percentage of variance explained (between-group variance/total variance) against the different number of clusters in elbow plot analysis (Supplementary Fig. S3A–C for 1k, 5k, and 10k data, respectively). From the elbow plots, we can see that the number of clusters was set in an acceptable range of the explained variance (between the steepest increase and the flattening point), suggesting that Seurat's method delineated an appropriate number of clusters in the 1k, 5k, and 10k data. Especially, although 5× more cells in the 5k data did not proportionally increase the number of clusters from the 1k data, the defined clusters explain a very similar percentage of the variance (∼16.25%), supporting the number of clusters in the 1k and 5k data again.

Another support comes when checking the dimension-reduced space (UMAP) of the data (Supplementary Fig. S3D–F for 1k, 5k, and 10k data, respectively) because distinct cell types are expected to be well separated on the UMAP. Because it is the case for the 1k, 5k, and 10k data, we believe that the numbers of defined clusters were set appropriately. Then, we filtered to keep cells with >1,000 UMI counts and 500 genes expressed. Cells with >15% UMI counts from mitochondrial genes were filtered out. Then, raw data were normalized by regressing against UMI count, mitochondrial mapping percentage, and ribosome genes mapping percentage using the SCTransform function. We ran PCA analysis and took the top 20 principal components as input to the FindNeighbors function. Finally, the FindClusters function was run with resolution set to 0.2 to identify cell communities. Cell types were annotated by matching the expression pattern of well-known marker genes for PBMC [29].

Mouse brain data. Aligned BAM file and clustering results of cortex and midbrain dorsal from 2 donors were downloaded from [11]. PCR duplicates were removed using UMI-tools [30] with the same parameters used for PBMC data. To keep consistent with the analysis performed by scAPA, we included only neurons, immune cells, astrocytes, oligodendrocytes, and vascular cells in our analysis. Differential expression analysis was performed by the FindAllMarkers function of the Seurat package with min.pct set to 0.25 and all other parameters as default.

### Investigating sample-specific up-regulated genes in GTEx

First, the mouse-human homology data were downloaded from the Vertebrate homology database in the Mouse Genome Informatics (MGI) database (http://www.informatics.jax.org/homology.shtml) and used to find homologs in human. Then, we ranked GTEx samples on the basis of the overlap between the upregulated genes and the homolog genes using a database that curates the up- and down-regulated genes for each GTEx sample, Enrichr [31]. Enrichr evaluates the overlap by combining *P*-value and odds ratio (Combined Score in Enrichr). We could not conduct this analysis for the midbrain dorsal region because GTEx did not collect data from that region.

### scMAPA algorithm

#### Step 0. Split aligned reads by cell clusters

scMAPA takes aligned BAM files and user-provided clustering information (e.g., cell type) as a match table to split the whole BAM file into each cluster using pysam. Clustering information should include all the categorical variables that the user would like to consider in the modeling, not only cell type. For example, when detecting APA genes in the mouse brain data, we used both brain region and cell type as covariate variables. After splitting, UMI-tools is used to remove the PCR duplicates by grouping reads that share the same UMI. Furthermore, scMAPA can identify false APA identifications due to internal priming of A-rich internal regions if >7 consecutive adenines with up to 1 mismatch exist in 10 nt downstream of the predicted proximal pA site [14]. In the PBMC 10k data, we identified that 90 of 3,574 APA events are due to suspected internal priming according to this standard.

#### Step 1. Pad reads along the 3ʹ-UTR after preprocessing

We transform aligned scRNA-Seq data that use 3ʹ selection and/or enrichment techniques in library construction (e.g., Drop-Seq, CEL-Seq, and 10x Genomics). A 3ʹ-biased read assigned to the 3ʹ-UTR of a gene represents the most 3ʹ end part of the transcript. With this reasoning, we extend the 3ʹ-biased read starting from the annotated 3ʹ-UTR start site to where the read ends (Step 1 in Fig. 1). After padding all the reads this way, we recalculate the read coverage on the 3ʹ-UTRs using “bedtools genomecov” in the Bedtools package [32] for each gene. Because the result represents the full-length read coverage of the transcript in the 3ʹ-UTR, our novel padding step enables us to use sensitive statistical approaches as follows.

#### Step 2. Quantify 3ʹ-UTR long/short isoforms

For further quantification, we formulate an optimization problem to infer the proximal pA site. Because our transformation reveals the proximal pA site where the read coverage changes, the optimization problem is minimizing the difference between the accumulated density of the isoforms and the input RNA-Seq read coverage as follows.

\begin{equation*} \left( {w_{kL}^*,w_{kS}^*,P_k^*} \right) = \mathop {{\mathrm{argmin}}}\limits_{w_{kL}^*,w_{kS}^* \ge 0,1 < {P_k} < L} \left\| {{R_{ki}} - \left( {{w_{kL}}{I_{kL}} + {w_{kS}}{I_{kP}}} \right)} \right\|_2^2
\end{equation*}

where ${w_{kL}}$ and ${w_{kS}}$ are the transcript abundances of long and short 3ʹ-UTR isoforms for cell cluster *k*, respectively. ${R_{ki}} = {[ {{R_{ki1}}, \ldots ,\,\,{R_{kij}},\,\, \ldots ,\,\,{R_{kiL}}} ]^T}$ is the corresponding read coverage at single-nucleotide resolution normalized by total sequencing depth. *L* is the length of the longest 3ʹ-UTR length from annotation, ${P_k}$ is the length of alternative proximal 3ʹ-UTR to be estimated, ${I_{kL}}$ is an indicator function with *L* times of 1, and ${I_{kP}}$ has ${P_k}$ times of 1 and $L - {P_k}$ times of 0. We solve this equation using quadratic programming [18] as was done in DaPars2. We describe how this is extended to identify genes with >2 pA sites at the end of this section.

#### Step 3. estimate APA significance across cell clusters

To make sure that only genes with strong APA signals among multiple cell types are identified, we first filter out genes in which only 1 pA site is detected in <3 cell types. Then, for each gene, we calculate the counts per million mapped reads (CPM) for long and short isoforms separately and average over all cell types. Only genes with an average CPM >10 for both long and short isoforms are kept. In addition to gene-wise filtering, we also apply cell-wise filtering for each passed gene to keep only cell types with ≥20 raw counts of reads in the model. For each gene, cell types with extremely low coverage (<20) will not be used to estimate the APA status.

To model the relationship between the long/short isoform identified above and the given cell types, we build logistic regression for each gene with log-odds of the event that the transcript uses the distal pA site (having long isoform) as the outcome and cell types as predictors using a weighted effect coding scheme. When scRNA-Seq data are collected from multiple samples or individuals, scMAPA can be easily extended to control the effect of unmatched confounding factors by adding them into the regression model:

\begin{equation*} \ell = \ln \frac{p}{{1 - p}} = {\beta _0} + \mathop \sum \nolimits_i^{n - 1} {\beta _i} * {C_i} + \mathop \sum \nolimits_j^m {\beta _j} * {V_j}, \end{equation*}

where *p*/(1 − *p*) is the odds of the transcript having a long isoform. ${\beta _i}$ and ${C_i}$ denote the coefficients and the binary indicator of each cell type, respectively. *n* is the number of cell types. Because 1 cell type needs to be chosen as a reference for model fitting, scMAPA fits the model twice to get the estimates of coefficients for all cell types. ${V_j}$ and ${\beta _j}$ denote the sample-specific binary confounding variables (e.g., clinical variable) and their coefficients, respectively. *m* is the number of confounding factors.

When there is no confounding factor, the likelihood ratio test (LRT) between cell type only model and null model is conducted to test the unadjusted effect of cell type, which is equivalent to the likelihood ratio χ^2^ test of independence between long/short isoforms and cell types. With the existence of confounding variables, LRT between the full model and confounding variables–only model is conducted to test the adjusted effect of cell type. *P*-values from all tests are further adjusted by the B-H procedure to control the FDR at 5%. In addition, to ensure that there is a significant change in effect size, the odds ratio of each cell type against the grand mean of all included cell types is calculated. There should be ≥1 cell type whose odds ratio is >0.25 for a gene to be called an APA gene.

Currently, scMAPA assumes only 2 pA sites in the 3ʹ-UTRs. However, our logistic model for Step 2 can be easily extended to detect >2 peaks if using other quantifiers that can consider >2 pA sites. For example, when only 2 peaks are detected for a gene, a binary logistic regression model would be fitted. However, when >2 peaks are detected for a gene, a multinomial logistic regression model would be fitted. To the our knowledge, because the only current tool that detects >2 peaks is scAPA, a multinomial logistic regression model is only compatible with the peak detection result of scAPA. LRT test is used to estimate the significance of APA among multiple peaks and cell types similarly.

#### Identification of cluster-specific 3ʹ-UTR dynamics

For the genes where significant APA dynamics is detected, scMAPA further analyses which cell type significantly contributes to the APA in which direction within each gene. By using a weighted effect coding scheme, each coefficient in the logistic regression can be interpreted as a measurement of deviation from the grand mean of all cells. This grand mean is not the mean of all cell type means; rather it is the estimate of the proportion of long isoforms of all cells for each gene. So, the unbalanced cell population sizes, which are common in scRNA-Seq, would not affect the accuracy of estimation.

We use the following 2 criteria to determine the cluster-specific significant 3ʹ-UTR dynamics:

First, given coefficients estimated from logistic regression, we use the Wald test to determine the *P*-value of each coefficient. *P*-values among all genes with significant APA of the same cell type are further adjusted by FDR. Then, we further selected genes whose APA degrees change >2-fold. If the APA degree increases >2-fold, the respective gene is considered as 3ʹ-UTR lengthening; if the APA degree decreases <2-fold, the respective gene is considered as 3ʹ-UTR shortening. However, users can define a different cut-off value of fold change to call 3ʹ-UTR lengthening or shortening.

#### Identification of genes of >2 pA sites

scMAPA can be easily extended to detect >2 pA sites and subsequently identify their significant differential usage. To detect >2 pA sites, scMAPA uses a similar approach to DaPars as follows. Instead of optimizing the regression model with a fixed number of predictors (proximal and distal pA sites), the case with >2 pA sites across *n* cell types can be formulated as follows.

\begin{equation*} \left[ {\begin{array}{@{}*{4}{c}@{}} {{r_{11}}}&{{r_{12}}}& \cdots &{{r_{1n}}}\\ {{{\mathrm{r}}_{21}}}&{{r_{22}}}& \cdots &{{r_{2n}}}\\ \vdots & \vdots & \cdots & \vdots \\ {{r_{m1}}}&{{{\mathrm{r}}_{m2}}}& \cdots &{{r_{mn}}} \end{array}} \right] = {\left[ {\begin{array}{@{}*{4}{c}@{}} 1&1& \cdots &1\\ 0&1& \cdots &1\\ \vdots & \vdots & \cdots & \vdots \\ 0&0& \cdots &1 \end{array}} \right]_{m \times m}}{\left[ {\begin{array}{@{}*{3}{c}@{}} {{w_{11}}}& \cdots &{{w_{1{\mathrm{n}}}}}\\ {{w_{21}}}& \cdots &{{w_{21}}}\\ \vdots & \cdots & \vdots \\ {{w_{m1}}}& \cdots &{{w_{mn}}} \end{array}} \right]_{m \times n}}
\end{equation*}

where *m* is the length of the longest 3ʹ-UTR of a transcript. The quantity ${w_{ij}}$ is the estimated abundance of 1 possible 3ʹ-UTR *i* in cell type *j*. Then, detecting multiple pA sites and estimating the abundance can be optimized by a LASSO regularization, in which the following equation should be optimized.

\begin{equation*} \mathop {{\mathrm{argmin}}}\limits_W \frac{1}{2}\left\| {C - {\mathrm{MW}}} \right\|_2^2 + {\mathrm{\lambda }}{\left\| W \right\|_1}
\end{equation*}

While the number of non-zero ${{\mathrm{w}}_{{\mathrm{ij}}}}$ indicates the number of pA sites for this gene, scMAPA will consider the genes with ≤4 estimated non-zero ${{\mathrm{w}}_{{\mathrm{ij}}}}$ by default, which can be further changed by the user. While this would avoid overfitting, we expect the default value to allow us to capture most genes according to a recent study on the number of pA sites for genes [33].

After pA site detection, the binomial logistic regression could be extended to a multinomial logistic regression to identify differential pA site usage when >2 pA sites exist. If in total *P* pA sites are detected by the pA site detection module, the differential pA site identification could be modeled as follows:

\begin{equation*} {\mathrm{Prob}}\left( {{\mathrm{P}}{{\mathrm{A}}_i} = p} \right) = \frac{{{e^{{{\mathrm{\beta }}_p}}} \cdot {X_i}}}{{\mathop \sum \nolimits_{k = 1}^p {{\mathrm{e}}^{{{\mathrm{\beta }}_k}}} \cdot {X_i}}}, \end{equation*}

where *p* is 1 of the *p* pA sites. The quantity ${X_i}$ is a row vector of features of an observed transcript. ${{\mathrm{\beta }}_p}$ is the coefficients associated with pA site*p*.

### Simulation

First, we used Splatter [34], a widely known scRNA-Seq simulator, to simulate the cell-level count matrix, which acts as the base of synthetic data. Splatter was trained by unfiltered mouse brain data and set to generate count matrices containing 5,000 genes and 3,000 cells. The matrix then collapsed into 5 columns, representing the total count of 5 cell groups. We call this 5,000 × 5 matrix a cluster-level count matrix.

From the analyses of PBMC and mouse brain data, we found that the standard deviation of PDUI (percentage of distal polyA site usage, which is equivalent to the proportion of long isoforms) of each gene could act as a classifier of APA gene and non-APA gene. On that basis, the standard deviation of PDUI for APA genes in synthetic data is estimated by calculating the mean of standard deviations of PDUI from APA genes detected by both scMAPA and scAPA from mouse brain data. Similarly, the standard deviation of PDUI for non-APA genes was estimated by calculating the mean of standard deviations of PDUI from genes identified as non-APA by both scMAPA and scAPA. With the estimated standard deviations, a PDUI matrix with the same size (5,000 × 5) as the cluster-level count matrices was generated. Each row of the PDUI matrix has a standard deviation equal to either the estimated standard deviation for the APA gene or the non-APA gene. This is achieved by centering 5 randomly selected numbers from standard normal distribution to 0. Then multiply the desired standard deviation by these centered numbers and add them to the desired mean. The mean of each row was randomly picked from 0.05 to 0.95. Because the estimated SD_isoprop_ values are averaged to 0.127 and 0.009 for the APA and the non-APA genes, respectively, we generated simulation data with SD_isoprop_ for APA genes in a range centered on 0.13 while fixing that for non-APAs at 0.009. The rows representing true APA genes were randomly selected. Then, each number in the cluster-level count matrix is divided into the count of long isoforms and the count of short isoforms by multiplying and PDUI matrix or (1 − PDUI matrix), respectively. Finally, the Pearson χ^2^-squared test (scAPA) or logistic regression model + LRT (scMAPA) could be applied to assess the performance of these 3 methods. For each repeat of simulation, the PDUI matrix is regenerated but the cluster-level count matrix stays the same for the sake of computational burden. Every simulation design was repeated 100 times to derive summarized statistics.

To examine the effect of experimental design on statistical power to detect significant APA genes, we assess the performance of scMAPA and scAPA in the following aspects: (i) To test the effect of unbalanced cell populations, the proportions of 5 cell types in the synthetic cell-level count matrices were set to 3 scenarios with different distribution of cell-type populations: (20%, 20%, 20%, 20%, 20%), (30%, 17.5%, 17.5%, 17.5%, 17.5%), and (50%, 12.5%, 12.5%, 12.5%, 12.5%). (ii) To test the effect of the proportion of true APA genes, we set 3 levels of true APA proportions, 5%, 10%, and 20%. (iii) To test the effect of the extent of APA dynamics, instead of using mean of standard deviations, we set the standard deviations of true APA genes in the simulated PDUI matrix to the 15 equally spaced sequence of numbers between the first quartile and the third quartile of standard deviations estimated from APA genes in mouse brain data. In total, there were 9 scenarios, corresponding to 9 combinations of factors (i) and (ii). When testing factor (iii), we chose balanced cell type proportion (0.2, 0.2, 0.2, 0.2, 0.2) and 10% true APA genes.

#### Assessing accuracy of pA site estimation

To assess the pA site/peak interval prediction accuracy, we used peak lists or pA site lists from scMAPA, scAPA, and Sierra on PBMC data. The estimation accuracy is measured by the percentage of the predicted peaks or pA sites overlapped with pA sites annotated in PolyASite 2.0. Because it is meaningless to find the overlap between 2-point estimates, we expanded the point position from the annotation database to an interval by manually adding a distance ranging from 10 to 150 bp in a 10-bp increment to both sides of the annotated pA sites. scMAPA gives a point estimate of pA site as predicted proximal pA site and Sierra gives 2-point estimates as fit max position and max position. To make the comparison more comprehensive, we calculated the midpoint of peak interval as the pseudo point estimate of scAPA. The point estimates from these methods are considered as supported by the annotation database if the point position falls in the annotated interval (annotated pA site ± distance). For peak intervals estimated by scAPA and Sierra, as long as there is 1 bp overlap between the estimated interval and the annotated interval (either start or end of estimated interval falls in annotated pA site ± distance), the estimate would be considered as supported by annotation database. Then, the percentage supported by annotation is calculated as the number of pA sites or peak intervals supported by the annotation database divided by total peaks detected for each method.

### Running scDAPA, scAPA, and Sierra

Sierra and scDAPA were run with default parameters. scAPA was run with default parameters and intronic regions omitted. The genes with a CPM of <10 were filtered out. We want to point out that scAPA uses the chisq.test function in R to estimate the significance of dynamic pA site usage among multiple clusters. This potentially makes the identification of scAPA more conservative than other tools in the multi-group setting because it does not allow any cell type to have 0 count because R's chisq.test would return NA as *P*-value if there is 0 presented in the count table. However, it is common to observe that a few cell types would not express certain genes in scRNA-Seq, especially when the whole cell population is split into >5 clusters (cell types), which is typical for complex biological systems.

To compare scDAPA and Sierra with scAPA and scMAPA in multiple-cluster settings, because scDAPA and Sierra identify APA genes only between cell cluster pairs, we combined the pairwise significant APA genes in each method separately. After controlling FDR on the combined APA genes, we called APA genes if they are significant in any of the pairwise identifications.

### Controlling undesired source of variance in cell-type–specific identification of APA genes

To compare the running modes, we first divided the mouse brain data into 10 cell groups by cell type and brain region (5 cell types × 2 brain regions). In each data group, we quantified the APA isoforms using scMAPA in 2 running modes, referred to as brain-region–confounding/controlled in the main text. The brain-region–confounding model is formulated as

\begin{equation*} \mathrm{ APA}\ \mathrm{ Isoform} \sim \mathrm{ cell}\ \mathrm{ type}. \end{equation*}

And the brain-region–controlled model is formulated as

\begin{equation*} \mathrm{ APA}\ \mathrm{ Isoform} \sim \mathrm{ cell}\ \mathrm{ type} + {\mathrm{brain\ region}}{\mathrm{.}}
\end{equation*}

## Availability of Supporting Source Code and Requirements

Project name: scMAPA

Project home page: https://github.com/ybai3/scMAPA

RRID:SCR_021822

biotoolsID: biotools:scmapa

Operating system: Platform independent

Programming language: R

License: GNU GPL

## Data Availability

An archival copy of the code and other supporting data are available via the GigaScience database GigaDB [35].

## Additional Files

**Supplementary Figure S1**. Comparison of bioinformatic tools and statistical methods to identify dynamic APAs in scRNA-Seq data.

**Supplementary Figure S2**. Performance assessment on the statistical component of scMAPA and scAPA using simulated data.

**Supplementary Figure S3**. Performance assessment on scMAPA, scAPA, Sierra, and scDAPA using PBMC data.

**Supplementary Figure S4**. APA analysis across multiple cell types using scMAPA on mouse brain data.

**Supplementary Figure S5**. Functional analysis on cell-type/brain-region specific APA genes identified by scMAPA.

**Supplementary Table S1**. Cell type annotation based on marker genes curated in CellMarker20 for 10k, 5k, and 1k in the PBMC data.

**Supplementary Table S2**. Detailed information of APA genes detected by scMAPA, scAPA, scDAPA, and Sierra on the PBMC data including Ingenuity Pathway Analysis (IPA) analysis result.

**Supplementary Table S3**. scMAPA estimation result for cell-type-specific APA genes on the mouse brain data.

**Supplementary Table S4**. Result of IPA comparison analysis on the “Disease & Function” terms enriched for APA genes identified uniquely by scAPA, scMAPA and commonly by both on the mouse brain data.

**Supplementary Table S5**. Result of IPA comparison analysis on the “Disease & Function” terms enriched for APA genes identified uniquely in astrocyte, immune, oligos, vascular, and neuron cells.

**Supplementary Table S6**. scMAPA estimates on the input data that are split by cell type and brain region either with brain region as a confounder or not.

**Supplementary Table S7**. IPA upstream regulator analysis result (enrichment p-value) on APA genes that are supposed to be brain-region-specific and non-specific.

giac033_GIGA-D-21-00240_Original_Submission

giac033_GIGA-D-21-00240_Revision_1

giac033_GIGA-D-21-00240_Revision_2

giac033_Response_to_Reviewer_Comments_Original_Submission

giac033_Response_to_Reviewer_Comments_Revision_1

giac033_Reviewer_1_Report_Original_Submission Bin Tian -- 9/1/2021 Reviewed

giac033_Reviewer_2_Report_Original_Submission Christian Cole -- 10/3/2021 Reviewed

giac033_Supplemental_Files

## Abbreviations

APA: Alternative polyadenylation; B-H: Benjamini-Hochberg; bp: base pairs; FDR: false discovery rate; GTEx: Genotype-Tissue Expression; IPA: Ingenuity Pathway Analysis; LRT: likelihood ratio test; MGI: Mouse Genomic Informatics; miRNA: microRNA; NIH: National Institutes of Health; pA: polyadenylation; PBMC: peripheral blood mononuclear cells; PCA: principal component analysis; scRNA-Seq: single-cell RNA sequencing; SD_isoprop_: standard deviation of the proportions of the long and short isoforms across all cell types; tSNE: t-distributed stochastic neighbor embedding; UTR: untranslated region; CPM: Counts per million mapped reads.

## Competing Interests

The authors declare that they have no competing interests.

## Funding

H.J.P. was supported by the Joan Gollin Gaines Cancer Research Fund at the University of Pittsburgh, and the UPMC Hillman Cancer Center Biostatistics Shared Resource that is supported in part by award P30CA047904 at the NIH. S.K. was supported by K01 HL153792 at the NIH.

## Conflict of interests

The authors declare that they have no conflict of interests.

## Authors' Contributions

H.J.P. and Y.B. conceived the project and designed the experiments. Y.B. and Z.F. implemented the software. Y.B., Y.Q., and R.M.M. performed the analysis. S.K., K.N., H.M.Z., R.K., and Q.S.P. interpreted the results statistically and/or biologically.
