# Supplementary material for: scMAPA: Identification of cell-type–specific alternative polyadenylation in complex tissues
Source: Gigascience. 2022 Apr 30;11:giac033. doi: 10.1093/gigascience/giac033 (PMC9055853; doi:10.1093/gigascience/giac033)
Supplement: giac033_GIGA-D-21-00240_Original_Submission [file giac033_giga-d-21-00240_original_submission.pdf]

GigaScience

scMAPA: Identification of Cell-type-specific Alternative Polyadenylation in Complex Tissues

--Manuscript Draft--

**Manuscript Number:**	GIGA-D-21-00240
**Full Title:**	scMAPA: Identification of Cell-type-specific Alternative Polyadenylation in Complex Tissues
**Article Type:**	Technical Note
**Funding Information:**	Joan Gollin Gaines Cancer Research Fund — Dr Hyun Jung Park<br>National Cancer Institute (P30CA047904) — Dr Hyun Jung Park
**Abstract:**	Alternative polyadenylation (APA) causes shortening or lengthening of the 3′-untranslated region (3′-UTR) of genes (APA genes) in diverse cellular processes such as cell proliferation and differentiation (cell-type-specific APA). To identify cell-type-specific APA genes in scRNA-Seq data, current bioinformatic methods have several limitations. First, they assume certain read coverage shapes in the scRNA-Seq data, which can be violated in multiple APA genes. Second, their identification is limited between two cell types and not directly applicable to the data of multiple cell types. Third, they do not control confounders and may introduce noise to the elucidation of cell-type-specific functions of APA genes. To address these limitations, we developed a combination of a computational change-point algorithm and a statistical model, single-cell Multi-group identification of APA (scMAPA). To release the assumptions on the read coverage shape, scMAPA formulates a change-point problem after transforming the 3′ biased scRNA-Seq data to represent the full-length 3′UTR signal. To identify cell-type-specific APA genes while controlling confounders, scMAPA models APA isoforms with cell type and confounder information. In our novel simulation data and human peripheral blood monocellular data, scMAPA outperforms existing methods in terms of sensitivity, robustness, and stability. In mouse brain data consisting of multiple cell types sampled from multiple regions, scMAPA identifies cell-type-specific APA genes, elucidating novel roles of APA for dividing immune cells and differentiated neuron cells and in multiple brain disorders. Altogether, scMAPA elucidates the cell-type-specific function of APA events and sheds novel insights into the functional roles of APA events in complex tissues.
**Corresponding Author:**	Hyun Jung Park<br>University of Pittsburgh<br>Pittsburgh, PA UNITED STATES
**Corresponding Author Secondary Information:**	
**Corresponding Author's Institution:**	University of Pittsburgh
**Corresponding Author's Secondary Institution:**	
**First Author:**	Hyun Jung Park
**First Author Secondary Information:**	
**Order of Authors:**	Hyun Jung Park<br>Yulong Bai<br>Yidi Qin<br>Zhenjiang Fan<br>Robert Morrison<br>KyongNyon Nam<br>Hassane Zarour

Powered by Editorial Manager® and ProduXion Manager® from Aries Systems Corporation

	Radosveta Koldamova
	Quasar Padiath
	Soyeon Kim
**Order of Authors Secondary Information:**	
**Additional Information:**	
**Question**	**Response**
Are you submitting this manuscript to a special series or article collection?	No
**Experimental design and statistics**<br><br>Full details of the experimental design and statistical methods used should be given in the Methods section, as detailed in our Minimum Standards Reporting Checklist. Information essential to interpreting the data presented should be made available in the figure legends.<br><br>Have you included all the information requested in your manuscript?	Yes
**Resources**<br><br>A description of all resources used, including antibodies, cell lines, animals and software tools, with enough information to allow them to be uniquely identified, should be included in the Methods section. Authors are strongly encouraged to cite Research Resource Identifiers (RRIDs) for antibodies, model organisms and tools, where possible.<br><br>Have you included the information requested as detailed in our Minimum Standards Reporting Checklist?	Yes
**Availability of data and materials**<br><br>All datasets and code on which the conclusions of the paper rely must be either included in your submission or deposited in publicly available repositories (where available and ethically	Yes

Powered by Editorial Manager® and ProduXion Manager® from Aries Systems Corporation

appropriate), referencing such data using a unique identifier in the references and in the “Availability of Data and Materials” section of your manuscript.<br><br>Have you have met the above requirement as detailed in our Minimum Standards Reporting Checklist?	

Powered by Editorial Manager® and ProduXion Manager® from Aries Systems Corporation

Manuscript in docx Click here to access/download;Manuscript;ver24.docx

scMAPA: Identification of Cell-type-specific Alternative Polyadenylation in Complex Tissues

Yulong Bai[1], Yidi Qin[1], Zhenjiang Fan[2], Robert M. Morrison[5,6,7], KyongNyon Nam[3], Hassane Mohamed Zarour[5,6], Radosveta Koldamova[3], Quasar Saleem Padiath[1,4], Soyeon Kim[7,8†], Hyun Jung Park[1†]

[1] Department of Human Genetics, Graduate School of Public Health, University of Pittsburgh, Pittsburgh, USA

[2] Department of Computer Science, School of Computing and Information, University of Pittsburgh, Pittsburgh, USA

[3]Department of Environmental and Occupational Health, Graduate school of Public Health, University of Pittsburgh, Pittsburgh, USA

[4]Department of Neurobiology, School of Medicine, University of Pittsburgh, Pittsburgh, USA

[5]Department of Medicine and Division of Hematology/Oncology, University of Pittsburgh, School of Medicine, Pittsburgh, USA

[6]Department of Immunology, University of Pittsburgh, School of Medicine, Pittsburgh, USA

[7]Department of Computational and Systems Biology, University of Pittsburgh Medical Center, Pittsburgh, USA

[8]Department of Pediatrics, University of Pittsburgh Medical Center, Pittsburgh, USA

[9]Division of Pulmonary Medicine, Children's Hospital of Pittsburgh of UPMC, Pittsburgh, Pennsylvania, USA

†senior author; Correspondence: hyp15@pitt.edu (H.J.P.)

ABSTRACT

Background: Alternative polyadenylation (APA) causes shortening or lengthening of the 3′-untranslated region (3′-UTR) of genes (APA genes) in diverse cellular processes such as cell proliferation and differentiation (cell-type-specific APA). To identify cell-type-specific APA genes in scRNA-Seq data, current bioinformatic methods have several limitations. First, they assume certain read coverage shapes in the scRNA-Seq data, which can be violated in multiple APA genes. Second, their identification is limited between two cell types and not directly applicable to the data of multiple cell types. Third, they do not control confounders and may introduce noise to the elucidation of cell-type-specific functions of APA genes.

Findings: To address these limitations, we developed a combination of a computational change-point algorithm and a statistical model, single-cell Multi-group identification of APA (scMAPA). To release the assumptions on the read coverage shape, scMAPA formulates a change-point problem after transforming the 3′ biased scRNA-Seq data to represent the full-length 3′UTR signal. To identify cell-type-specific APA genes while controlling confounders, scMAPA models APA isoforms with cell type and confounder information. In our novel simulation data and human peripheral blood monocellular data, scMAPA outperforms existing methods in terms of sensitivity, robustness, and stability. In mouse brain data consisting of multiple cell types sampled from multiple regions, scMAPA identifies cell-type-specific APA genes, elucidating novel roles of APA for dividing immune cells and differentiated neuron cells and in multiple brain disorders.

Conclusions: Altogether, scMAPA elucidates the cell-type-specific function of APA events and sheds novel insights into the functional roles of APA events in complex tissues.

Keywords: post-transcriptional regulation, alternative polyadenylation, single-cell RNA, cell-type-specific regulation, confounding factors

FINDINGS

Introduction

Many mammalian messenger RNAs contain multiple polyadenylation (pA) sites, e.g., proximal and distal, in their 3′-untranslated region (3′-UTR) [1], [2]. Using multiple pA sites in each gene, alternative polyadenylation (APA) post-transcriptionally produces multiple APA isoforms with various 3′-UTR lengths. These APA events are involved in diverse cellular processes such as cell proliferation and differentiation in particular cell types. For example, cancer cells of diverse types are reported to undergo widespread 3′UTR shortening events [3], whereas senescent cells tend to show widespread 3′UTR lengthening events [4]. To identify such APA genes for each cell type (cell-type-specific APA genes) in complex tissues, developing a computational method that accurately analyzes single-cell RNA sequencing (scRNA-Seq) data is essential since the data presents the cell-type-specific transcriptome.

To identify cell-type-specific APA genes in scRNA-Seq data, several bioinformatic methods have been developed, such as scDAPA[5], Sierra [6], and scAPA [7]. Although they have various strengths, they also have several limitations to be used for complex tissue data.

First, they only consider certain read coverage shapes in the input scRNA-Seq data to estimate APA events. This is because several scRNA-Seq techniques generate the 3′ enriched reads and the accumulation of the reads that originate from the same APA isoform forms a peak. To identify the signal part of the peak from noise, the existing methods assume certain signal shapes in their peak calling. For example, scAPA utilizes findPeaks module in Homer package [8] with the preset peak size and height. However, these assumptions can be violated in multiple genes across multiple cell types. For example, one would be interested in quantifying APA isoforms of FLT3 and GATA2 in the scRNA-Seq data on Peripheral Blood Monocellular Cells (PBMC) of a healthy donor (10k in https://www.10xgenomics.com/) since their abnormality may lead to blood disorders [9], [10]. However, their 3′ tags form peaks with different sizes and heights across various cell types (**Fig. 1A, C**) that the existing methods would not be able to identify peaks from some of the cell types. Second, the existing methods cannot identify cell-type-specific APA genes when the scRNA-Seq data contains more than two cell types, which is typical for complex tissues. scDAPA and Sierra are only able to compare cell types in a pairwise fashion, which limits their ability for global comparison when more than two cell types exist. While scAPA is the only method to identify APA genes for multiple cell types, it identifies genes in which the APA isoform ratio (the ratio of long and short 3′-UTR isoforms) varies across the cell types and does not further identify which specific cell types drive this variation. Third, the existing methods do not adjust for confounding factors. Confounding can arise when cells are affected by factors that are not parts of the research hypothesis under investigation. For example, when complex tissue data consist of multiple cell types sampled from various regions, one may need to adjust for the sample region as a confounder if the hypothesis focuses only on identifying cell-type-specific APA genes. Fourth, there is no simulation platform to compare statistical power

and specificity of the methods identifying APA genes in scRNA-Seq data. Although such a platform is necessary to evaluate the methods with the ground truth, it has been challenging to simulate APA and non-APA genes since it is not clear how the read coverage shapes differ between APA and non-APA genes.

To address these limitations, we developed a combination of a computational optimization algorithm and a statistical model, single-cell Multi-group identification of APA (scMAPA). To address the first limitation and quantify APA isoforms without assumptions on the read coverage shape, scMAPA first transforms the input scRNA-Seq data and then formulates a change-point detection problem on the transformed data. First, scMAPA transforms the 3′-enriched signal of scRNA-Seq data to represent the full-length 3′UTR signal. For FLT3 and GATA2 in the PBMC of a healthy donor, this transformation made the APA short and long isoforms readily distinguishable across all cell types regardless of the differences in read coverage shape (**Fig. 1B, D**). Then, on the transformed coverage shapes, scMAPA quantifies APA isoforms by detecting a change-point. To address the second and the third limitations to identify cell-type-specific APA genes and to control confounders respectively, scMAPA considers cell type information and adjusts confounders by developing a statistical model with them as covariates. By incorporating such covariates in the model, scMAPA estimates statistical significance and the effect size of each APA event in each cell type while controlling the confounders. To address the fourth limitation and simulate APA genes, we identified a common feature of APA genes in real data, a high variance in the APA isoform ratios across cell types and simulate the APA isoform specific count matrix based on the common feature. Since this simulation platform does not generate data at the level of read coverage shape, it can generate the ground truth APA genes without having to resolve the difference between APA and non-APA

genes in the read coverage shape. By systematically addressing these limitations, scMAPA accurately and robustly identifies cell-type-specific APA genes and facilitates a systematic understanding of APA regulation in complex tissues in this manuscript.

Single-cell multi-group identification of alternative polyadenylation (scMAPA)

To identify cell-type-specific APA genes accurately and robustly, scMAPA combines a computational algorithm and a statistical model. scMAPA transforms the input data, formulates a change-point detection problem on the transformed data, and quantify APA isoforms by solving the problem using an optimization algorithm. First, scMAPA transforms each read in the scRNA-Seq data by padding it from the annotated 3'UTR start site to where the read ends (step 1 in **Fig. 1E**). While the scRNA-Seq reads are usually 3' biased due to the 3′ selection and enrichment techniques in the library construction step, the transformed reads will represent the read coverage shape across the 3'UTRs. Second, scMAPA identifies a pA site that minimizes the difference between the expected coverage shape of the inferred APA isoforms and the accumulated observed coverage (change-point, step 2 in **Fig. 1E**). Since the difference can be calculated by a quadratic function, scMAPA detects the change-point by quadratic programming[11]. To solve this problem for multiple cell types in scRNA-Seq data, scMAPA extends multiple modules of DaPars2 [12], which used the quadratic programming approach to identify APA genes in bulk RNA-Seq data.

To simultaneously identify APA genes across cell types and for each cell type based on the APA isoforms quantified, scMAPA develops a multinomial regression model that explicitly models each APA isoform (step 3 in **Fig. 1E**) with covariates representing cell types and

confounders (step 4 in **Fig. 1E**). On the model, scMAPA uses the log-likelihood test and the Wald test and identifies across-cell-type APA genes and cell-type-specific APA genes, respectively. Altogether, scMAPA is the first method to simultaneously identify across-cell-type and cell-type-specific APA genes in scRNA-Seq data of multiple cell types.

scMAPA outperforms the other method in sensitivity for the multi-group setting

To assess the performance of scMAPA using the ground truth, we developed a novel simulation platform where APA isoform-specific expressions are simulated with parameters learned from real data. Especially, to make the simulations unbiased and biologically reasonable, it is based on a common feature of APA genes we identified in a mouse brain scRNA-Seq data consisting of multiple cell types [13] (neurons, astrocytes, immune cells, oligodendrocytes, and vascular, step 0 in **Fig. 2A**) in the following procedure. First, we determined APA genes across the cell types as those identified by both scAPA and scMAPA. We used only scAPA and scMAPA since they are the only methods designed for more than two cell types. Since both methods determine the APA genes as those whose APA long and short isoforms vary highly among cell types, we decided to quantify a common feature of the APA genes by calculating the proportion of the long and short isoforms in each cell type and the standard deviation of the proportions across the five cell types ($SD_{isoprop}$, see Methods). To validate the effectiveness of this measure, we calculated $SD_{isoprop}$ values for non-APA genes that scAPA and scMAPA agreed on in the data. We found that high $SD_{isoprop}$ values significantly distinguish APA genes from non-APA genes (0.127 vs. 0.009 of $SD_{isoprop}$ on average, $p<2.2e^{-16}$, **S. Fig. 2A**), suggesting that it is reasonable to simulate APA genes to have high $SD_{isoprop}$ values in the data of multiple (≥2) cell types (multi-group setting).

To simulate APA genes with high $SD_{isoprop}$ values across 5 simulated cell clusters, we first selected the $SD_{isoprop}$ values estimated for the APA genes of the mouse brain data. Then, based on the selected $SD_{isoprop}$ value, which is the standard deviation of APA isoform ratios, we randomly generated the APA isoform ratios across the 5 simulated cell clusters (step 2 in **Fig. 2A**). After separately simulating gene expression values (step 3 in **Fig. 2A**), these isoform ratios were used to divide the gene expression values into APA long and short isoform expressions (step 4 in **Fig. 2A**). Similarly, we simulated the APA long and short isoform expressions for non-APA genes based on the $SD_{isoprop}$ values estimated for the non-APA genes of the mouse brain data. On the simulated APA isoform expressions for APA and non-APA genes, we ran scMAPA and scAPA to assess their sensitivity and specificity. In the first scenario simulating 500 APA and 4,500 non-APA genes, we varied $SD_{isoprop}$ values for APA genes in the range observed in the mouse brain data (0.06 to 0.18, **S. Fig. 2A**). Across all simulated $SD_{isoprop}$ values, scMAPA consistently outperforms scAPA with higher sensitivity (**Fig. 2B**) while having a similar specificity (**Fig. 2C**). In assessing specificity, we did not vary $SD_{isoprop}$ values for non-APA genes, since the mouse brain data showed a narrow range of $SD_{isoprop}$ values for non-APA genes (**S. Fig. 2A**). In the second scenario, we varied the number of APA and non-APA genes and the cell group size while fixing the $SD_{isoprop}$ values for APA and non-APA genes (to 0.127 and 0.009, respectively). With various numbers of true APA genes (250, 500, and 1,000), scMAPA consistently outperforms scAPA in terms of sensitivity (**Fig. 2D and S. Fig. 2B, D**) with a slight loss of specificity (**Fig. 2E and S. Fig. 2C, E, F**). To sum, scMAPA outperforms scAPA in various simulation scenarios in terms of sensitivity with a similar level of specificity.

scMAPA outperforms existing methods in identifying APA isoforms with high robustness

To assess the performance of scMAPA using real data, we used three PBMC data sets of various numbers of cells (1k, 5k, and 10k data representing the number of cells) downloaded from 10x Genomics website. Especially, to assess the performance across multiple cell types, we defined different numbers of cell types (6, 8, and 13 types for 1k, 5k, and 10k data respectively) based on Seurat's graph-based clustering [14] and annotated their cell types based on established marker genes [15] (see Methods, **S. Table 1**). To assess the accuracy of scMAPA in identifying annotated pA sites, we identified pA sites in the 10k and 5k data using scMAPA, scAPA, and Sierra. scDAPA was not included in this comparison, because it does not return results that are compatible for the comparison, such as pA peaks, sites, or intervals. Among the identified pA sites, we calculated the proportion of them that are close to the annotated pA sites in PolyASite 2.0[16] (see Methods). scMAPA consistently outperformed the other methods by identifying the highest proportion of the annotated pA sites across all degrees of proximity (**Fig. 3A**, **S. Fig. 3A, B**). This result suggests the outperformance of scMAPA in identifying possible bona fide APA events originated from the annotated pA sites.

We further evaluated the robustness of the methods in two ways. First, we ran scMAPA, scAPA, scDAPA, and Sierra to identify APA genes in the 1k, 5k, and 10k PBMC data. Since the 1k, 5k, and 10k data sets comprise similar sets of cell types from healthy adults (1k and 10k from the same donor and 5k from another healthy donor, **S. Table 1**), the APA genes are expected to overlap across the data sets. Thus, a high percentage of APA genes identified commonly across the data sets would indicate the robustness of the methods to the number of cells in the data. Although Sierra and scDAPA cannot identify APA genes directly from multiple (>2) cell types, we artificially identified the APA genes for multiple cell types by combining all pairwise

identifications after FDR control (see Methods). Compared to the competing methods, scMAPA identified a two-fold higher percentage of APA genes commonly across the 3 types of the data sets (40.7% vs. 18.9%, 11.6%, and 18.6% respectively, **Fig. 3B**), showing that scMAPA identifies APA genes robustly to the number of cells in the data. Second, from the 10k data comprising the total of 13 cell types, we randomly sampled various numbers of cell types (5, 7, 9, and 11) from the 13 cell types and ran scMAPA and scAPA separately in each sample. For direct comparison, we compared scMAPA only with scAPA, the only other method that can directly handle the multi-group setting. In the APA genes identified in each sample (sample APA genes), we calculated the overlap with those identified using all the 13 cell types (total APA genes). Then, we calculated APA agreement ratio, defined as the number of the overlap between the sample and total APA genes normalized by the number of total APA genes. In all the numbers of cell types sampled, scMAPA outperforms scAPA with higher APA agreement ratios (**Fig. 3C**). Since the APA agreement ratio indicates the number of the total APA genes that are found in the sample APA genes, the result shows that scMAPA identifies APA genes robustly to the number of cell types in the data.

Further, to investigate if the APA genes identified by scMAPA are biologically reasonable, we performed Ingenuity Pathway Analysis (IPA) on 3,574 APA genes that scMAPA identified in the 10k PBMC data. Especially, to accurately investigate the APA genes' roles in PBMC biology, we set the 18,804 genes expressed in the data as the background (see Methods). This IPA analysis shows significant (B-H p-value $< 10^{-2}$) enrichments to 32 IPA terms that are characterized with keywords "blood" and "hematology" (**Fig. 3D**), suggesting that the APA genes identified by scMAPA can play important roles in PBMC biology.

To examine the unique contribution of scMAPA in characterizing the function of APA genes for PBMC biology, we manually inspected 1,432 APA genes that are identified only by scMAPA, not by other methods (scMAPA-unique APA genes, **S. Table 2**). In the scMAPA-unique APA genes, we found clear changes in the APA isoform ratios across the cell types and great potential to function for PBMC biology. For example, FLT3 and GATA2 are included in the scMAPA-unique APA genes and showed the dynamic APA isoform ratios across the cell types especially after the data transformation step of scMAPA (**Fig. 1B, D**). Interestingly, GATA2 is an APA gene in the scRNA-Seq data of bone marrow mononuclear cells from acute myeloid leukemia patients [17]. Since bone marrow is developmentally related to peripheral blood, GATA2 may undergo the APA event in the PBMC under similar molecular mechanisms. Together, scMAPA enables accurate and robust identification of biologically reasonable APA genes in the PBMC scRNA-Seq data.

scMAPA estimates APA effect size and identifies APA genes across multiple cell types

Compared to other methods, scMAPA is the only method that can estimate the effect size and the significance of APA events for each cell type in the multi-group setting (see Methods). To demonstrate how the APA effect size enables us to understand the post-transcriptional regulation in each cell type, we analyzed the mouse brain scRNA-Seq data comprising five major cell types: neurons, astrocytes, immune cells, oligodendrocytes, and vascular [13] (**Fig. 4A,** see Methods). First, to identify the distances among the cell types in terms of the APA effect size, scMAPA estimated the effect size of 3,223 genes significantly (B-H P-val < 0.05) identified as APA genes across the five cell types (**Fig. 4B**). Based on these effect sizes, we performed the PCA analysis (**Fig. 4C**) and calculated Euclidean distance (**S. Fig. 4A**) between the cell types.

While both the analyses supported the previous finding that immune and neuron cells are most different in terms of the APA effect size [7], they further revealed that immune cells are most different from all the other cell types. Second, to identify the overall relationships between the APA regulation and the gene expression regulation, we correlated the APA effect sizes of all the identified genes with their expression level. The result shows that the APA effect sizes are not correlated with their expression level in all the cell types (e.g., Spearman's $\rho < 0.05$ for all cell types, **S. Fig. 4D-H**), demonstrating that APA events are regulated independently of gene expression regulation in the mouse brain.

Further, cell-type-specific APA genes (3′-UTR shortening and lengthening genes) identified by scMAPA provide a systematic understanding of cellular status. Previous studies showed that APA is involved in regulating cell division status. For example, various types of dividing cells are associated with widespread 3′-UTR shortening [18], [19]. Likewise, differentiated and senescent cells are associated with widespread 3′-UTR lengthening [20], [21]. To systematically extend these findings that were made in cell line data [18], [20], [22] or heterogeneous tissue data [19], we ran scMAPA in the mouse data further to identify 438 significant (B-H P-val < 0.05) cell-type-specific APA genes in neurons, 891 in immune, 374 in astrocyte, 422 in vascular, and 430 in oligos with some overlaps across the cell types (**S. Fig. 4B**). A further division into 3′-UTR shortening and lengthening genes in each cell type (**Fig. 4D)** showed that 3′-UTR shortening and lengthening are significantly enriched in immune cells and neuron cells, respectively. As immune cells actively divide to dynamically regulate the immune system, the enriched 3′-UTR shortening may contribute to the active division. In the same sense, we could find a biological explanation for why 3′-UTR lengthening are enriched in neurons. While neurons do not divide once they are formed in the brain, our result suggests that the 3′-

UTR lengthening can play a significant role in keeping neuron cells from further dividing. Together, by identifying cell-type-specific APA genes, scMAPA systematically links the cellular APA profile to dividing immune cells and differentiated neuron cells.

Controlling confounders through scMAPA uncovers functions of APA genes which would be invisible due to the confounders

To show how scMAPA controls confounding factors and why it is important, we analyzed the mouse brain data consisting of 5 cell types collected from 2 brain regions (cortex and midbrain). Since some cell types were collected from multiple brain regions (**Fig. 4A, 5A**), some APA genes associated with a brain region can be mistakenly identified as cell-type-specific APA genes, which would further confound studying cell-type-specific functions of APA genes. To see if scMAPA can remove such false positive APA genes, we ran scMAPA with the brain region information (cortex and midbrain dorsal) as the confounder (confounder-adjusted scMAPA) and without the confounder (confounder-unadjusted model), separately. As the confounder-adjusted scMAPA and the confounder-unadjusted model identified 2,715 and 2,793 APA genes respectively (**S. Table 6**), 113 genes are no longer identified as significant APA gene after adjusting brain region. Thus, these APA genes are expected to be related to the confounders (confounder-related APA genes, **Fig. 5B**), which is the brain region it was sampled from (cortex and midbrain). To test if the 113 genes function specifically for the brain region, we test if they express highly specifically in the brain region. To conduct this test comprehensively, we identified their human homolog genes in the Mouse Genomic Informatics (MGI) homology database and compared expression of human homologs between cortex and other brain regions in the Genotype-Tissue Expression (GTEx) [23] (see Methods). The result shows that these APA

genes are significantly up-regulated in brain cortex compared to other brain regions (p-value=$5.8e^{-7}$, **Fig. 5D**), suggesting that their functions are specific to brain cortex. Since GTEx does not collect the expression data for midbrain, we did not conduct this analysis for midbrain. This result suggests that, when scMAPA sets a confounder, it can successfully distinguish and exclude the APA genes that are likely related to the confounder.

To demonstrate why distinguishing and excluding the confounder-related APA genes is critical for accurate downstream analysis, we further conducted IPA analysis on the 2,715 and 2,793 APA genes identified by the confounder-adjusted scMAPA and the confounder-unadjusted model respectively (confounder-adjusted and confounder-unadjusted APA genes, respectively). Comparing the IPA enrichment between confounder-adjusted and confounder-unadjusted APA genes, we found considerable differences in important terms for brain study: among the 24 terms to which the confounder-adjusted APA genes are uniquely and significantly (B-H P-value $< 10^{-2}$) enriched, 7 terms are directly related to brain diseases (**Fig. 5D**). For example, two terms with the keyword "mental retardation" are significantly enriched (B-H P-value $< 10^{-3.5}$) only for the confounder-adjusted APA genes. On the other hand, among the 30 terms to which the confounder-unadjusted APA genes are uniquely and significantly enriched, no term refers to a brain disease (**S. Fig. 5A**). This result suggests that the confounder-adjusted scMAPA uncovers the APA genes that can play critical roles in the brain disease, which would be invisible due to the confounding factors.

Supplemental material

APA regulation on expression

Previous studies have suggested that APA genes are more likely differentially expressed[1], [2], since either 3′-UTR shortening removes microRNA (miRNA) binding sites on the 3′-UTR and evades miRNA-mediated repression or 3′-UTR lengthening adds miRNA binding sites and enhance miRNA-mediated repression. Our analysis reaffirms the previous observations in the scRNA-Seq data.

scMAPA consensus with other methods

In the PBMC data, scMAPA results still recover most of the results from the other methods. To assess the overlap, we identified significant APA genes across all the cell types in scMAPA and scAPA. Since scDAPA and Sierra identify APA genes only between cell-type pairs, we combined the pairwise significant APA genes in each method separately. After controlling FDR on the combined APA genes, we called APA genes if they are significant in any of the pairwise identifications. While scMAPA identifies an intermediate number of APA genes between scDAPA and Sierra/scAPA (10k in **S. Fig. 3C** and 5k in **S. Fig. 3D**), more than half of the scMAPA's findings are found in other methods (59.9% for 10k and 51.9% for 5k). While scMAPA solves an optimization problem based on the padding of 3′ biased reads (step 1 in **Fig. 1C**), it successfully recovers most results from other methods, validating the use of scMAPA for comprehensive identification.

Cell-type-specific APA genes in 10k PBMC data

The global size differences in PBMC cells are different from in the mouse brain data in several aspects. First, 3'UTR lengthening occurs more than 3'UTR shortening in all the cell types (**S. Fig. 4E**). Second, however, the number of 3'UTR shortening genes is significantly correlated with that of lengthening genes across the cell types (P-value=$5e^{-5}$, **S. Fig. 4F**). Since both trends are

not shown in the mouse brain data, scMAPA elucidates the unique APA profiles of the PBMC data.

Specificity of high expression in 113 confounder-related APA genes for the brain cortex region

In demonstrating the high expression of the 113 confounder-related APA genes in the brain cortex region, we further investigated if the confounder-related APA genes are not down-regulated in neither brain vs. non-brain samples (**S. Fig. 5B)** nor cortex vs. non-cortex brain samples (**S. Fig. 5C)**. Also, this brain-region-specific expression pattern was not found for 2,715 APA genes identified by the confounder-adjusted scMAPA (**S. Fig. 5D, E, F, G**). Together with our analysis on up-regulation (**Fig. 5**), the results suggest that the 133 APA genes function specific to the brain region.

Discussion

To identify APA genes in scRNA-seq data for complex tissue data, we developed scMAPA that addresses several limitations in existing methods using a combination of a computational optimization algorithm and a statistical model. First, while existing methods detect APA signals with assumptions on the shape of the input data, scMAPA does not rely on such assumptions by formulating this task in quadratic programming. By solving this quadratic programming for genes with different read coverage shapes across cell types, scMAPA outperforms existing methods in accurately and robustly identifying APA genes in various simulated (**Fig. 2**) and PBMC data (**Fig. 3**). Second, scMAPA identifies APA genes specific to each cell type in a statistically rigorous model. These cell-type-specific APA genes elucidates their connections to the cell division status of immune and neuron cells in the mouse brain data (**Fig. 4**). Third,

scMAPA can control confounding factors. In the mouse brain data of five cell types collected from two brain regions, scMAPA can distinguish the 113 APA genes that are likely related to the brain regions. By removing the false positive APA genes from further analyses, scMAPA could clarify the functions of APA genes on brain diseases such as ‘mental retardation’ (**Fig. 5**). Lastly, we developed a novel simulation platform in which to assess statistical power of APA identification methods based on a common feature of APA genes, the high variation of APA long and short isoforms ($SD_{isoprop}$) across cell clusters.

When identifying the annotated pA sites, scMAPA makes point estimations of the pA sites. While other methods mainly produce interval estimates, point estimations are more directly relevant to further analyses than interval estimations, e.g., conducting omics data analyses and designing validation experiments. However, when point estimation methods are naively compared to interval estimation methods in terms of the distance to the annotated pA sites, point estimations produce generally disadvantageous results, because point estimation returns a single point while interval estimation returns two points (start and end of the interval) to measure the distance. For example, the interval estimations produce better results than the point estimations within both Sierra and scAPA (**S. Fig. 3A, B**). Even with this disadvantage of point estimation for comparison purposes, the point estimation of scMAPA outperforms the interval estimation results of Sierra and scAPA in identifying the annotated pA sites, showing a clear advantage of scMAPA (**Fig. 3A, S. Fig. 3A, B**).

A limitation of this paper is that, although scMAPA can consider more than two pA sites (see Methods), our analysis focused on the use of two pA sites (most distal and most proximal) for the following reasons. First, some of the methods that compare with scMAPA consider only two pA sites, e.g. scAPA. For fair comparisons, we limited scMAPA to consider two pA sites.

Second, we focused on this binary APA trend to make it easier to investigate across multiple cell types. We plan to consider more than two pA sites in complex tissues after characterizing the binary trend across multiple cell types. For example, after solving the quadratic programming with >2 pA sites and developing a multinomial logistic regression model with the identified pA sites in the mouse brain data, we can estimate the APA effect size for each use of the multiple pA sites.

scMAPA can be extended in the following directions in the future. First, the transformation step of scMAPA allows us to use other methods originally developed for bulk RNA-Seq data (e.g. APATrap [30], TAPAS [31]) to analyze scRNA-Seq data. Since the methods can identify APA genes in the full-length 3′ UTR signal of transcripts, scMAPA can employ such methods on the transformed scRNA-Seq data that represents the full-length 3′ UTR signal of transcripts. This extension can make those APA identification methods as reasonable alternatives since those methods are well established and studied in terms of sensitivity and specificity. Second, while existing methods developed for scRNA-Seq data are mostly designed for 3′biased scRNA-Seq data (e.g. 10x), scMAPA can be used for the scRNA-Seq data that are not 3′biased (e.g. Smart-seq2 [32]) simply by skipping the data transformation step, since the scRNA-Seq data already present the full-length 3′-UTRs.

Altogether, we developed scMAPA to identify APA genes in scRNA-Seq data of multiple cell types. With high sensitivity and robustness in addition to adjusting for confounders, scMAPA elucidates the cell-type-specific function of APA events, which is essential to shed novel insights into the functional roles of APA events in complex tissues.

METHODS

Processing data sets

PBMC data. Aligned BAM files were downloaded from the 10X genomics repository (https://support.10xgenomics.com/single-cell-gene-expression/datasets). According to the data description of 10X, 1K, and 10K data were generated from the same materials. 5K data was generated from different cells. PCR duplicates were removed using UMI-tools 1.0.0 with "--method=unique --extract-umi-method=tag --umi-tag=UB --cell-tag=CB". Cell clustering was performed using R package Seurat 3.1.4[24]. We filtered to keep cells with more than 1000 UMI counts and 500 genes expressed. Cells with more than 15% UMI counts from mitochondrial genes were filtered out. Then, raw data were normalized by regressing against UMI count, mitochondrial mapping percentage, and ribosome genes mapping percentage using SCTransform function. We ran PCA analysis and took the top 20 principal components as input to FindNeighbors function. Finally, FindClusters function was run with resolution set to 0.2 to identify cell communities. Cell types were annotated by matching the expression pattern of well-known marker genes for PBMC [15].

Mouse brain data. Aligned BAM file and clustering results of cortex and midbrain dorsal from two donors were downloaded from [13]. PCR duplicates were removed using UMI-tools[25] same parameters used for PBMC data. To keep consistent with the analysis performed by scAPA, we included only neurons, immune cells, astrocytes, oligos, and vascular cells in our analysis. Differential expression analysis was performed by FindAllMarkers function of Seurat package with min.pct set to 0.25 and all other parameters as default.

Investigating sample-specific up-regulated genes in GTEx

First, the mouse-human homology data was downloaded from the Vertebrate homology database in the Mouse Genome Informatics (MGI) (http://www.informatics.jax.org/homology.shtml) and used to find homologs in human. Then, we ranked GTEx samples based on the overlap between the upregulated genes and the homolog genes using a database that curates the up- and down-regulated genes for each GTEx sample, Enrichr [26]. Enrichr evaluates the overlap by combining p-value and odds ratio (Combined Score in Enrichr). We could not conduct this analysis for the midbrain dorsal region, since the GTEx did not collect data from the region.

scMAPA algorithm

Step 0. *Split aligned reads by cell clusters.*

scMAPA takes aligned BAM files and user-provided clustering information (e.g. cell type) as a match table to split the whole BAM file into each cluster using pysam. Clustering information should include all the categorical variables that the user would like to consider in the modeling, but not only cell type. For example, when detecting APA genes in the mouse brain data, we used both brain region and cell type as covariate variables. After splitting, UMI-tools is used to remove the PCR duplicates by grouping reads that sharing the same UMI. Further, scMAPA can identify false APA identifications due to internal priming of A-rich internal regions if more than 7 consecutive adenines with up to 1 mismatch exists in 10 nt downstream of the predicted proximal PA site[16]. In the PBMC 10K data, we identified that 90 out of 3574 APA events are due to suspected internal priming according to this standard.

Step 1. *Pad reads along the 3'UTR after preprocessing.*

We transform aligned scRNA-Seq data that utilize 3′ selection and/or enrichment techniques in library construction (e.g. Drop-Seq, CEL-Seq, and 10x Genomics). A 3′ biased read assigned to the 3′UTR of a gene represents the most 3′ end part of the transcript. With this reasoning, we extend the 3′ biased read starting from the annotated 3'UTR start site to where the read ends (Step 1 in **Fig. 1**). After padding all the reads this way, we recalculate the read coverage on the 3′UTRs using 'bedtools genomecov' in Bedtools package[27] for each gene. Since the result represents the full-length read coverage of the transcript in the 3′UTR, our novel padding step enables us to employ sensitive statistical approaches as follows.

Step 2. *Quantify 3'UTR long/short isoforms.*

For further quantification, we formulate an optimization problem to infer the proximal pA site. Since our transformation reveals the proximal pA site where the read coverage changes, the optimization problem is minimizing the difference between the accumulated density of the isoforms and the input RNA-Seq read coverage as follows.

$$(w_{kL}^*, w_{kS}^*, P_k^*) = \underset{w_{kL}^*, w_{kS}^* \geq 0, 1<P_k<L}{\operatorname{argmin}} || R_{ki} - (w_{kL} I_{kL} + w_{kS} I_{kP}) ||_2^2$$

where w_{kL} and w_{kS} are the transcript abundances of long and short 3′-UTR isoforms for cell cluster k, respectively. $R_{ki} = \left[R_{ki1}, \dots, R_{kij}, \dots, R_{kiL}\right]^T$ is the corresponding read coverage at single-nucleotide resolution normalized by total sequencing depth. L is the length of the longest 3′-UTR length from annotation, P_k is the length of alternative proximal 3′-UTR to be estimated, I_{kL} is an indicator function with L times of 1, andI_{kP} has P_k times of 1 and $L - P_k$ times of 0. We solve this equation using quadratic programming [19] as was done in DaPars2. We will describe how this is extended to identify genes with more than two pA sites at the end of this section.

Step 3*. estimate APA significance across cell clusters.*

To make sure only genes with strong APA signals among multiple cell types are identified, we first filter out genes in which only 1 PA site is detected in less than 3 cell types. Then, for each gene, we calculate the CPM for long and short isoforms separately and average over all cell types. Only genes with an average CPM larger than 10 for both long and short isoforms are kept. In addition to gene-wise filtering, we also apply cell-wise filtering on passed genes to let only cell types with at least 20 raw counts enter the model fitting step. This ensures the estimation of the coefficient would not be biased by cell types with extremely low counts.

To model the relationship between the long/short isoform identified above and the given cell types, we build logistic regression for each gene with log-odds of the event that transcript uses distal polyA site (having long isoform) as the outcome and cell types as predictors using weighted effect coding scheme. When scRNA-Seq data were collected from multiple samples or individuals, scMAPA can be easily extended to control the effect of unmatched confounding factors by adding them into the regression model:

$$\ell = \ln \frac{p}{1-p} = \beta_0 + \sum_{i}^{n-1} \beta_i * C_i + \sum_{j}^{m} \beta_j * V_j$$

where $\frac{p}{1-p}$ is the odds of the transcript having a long isoform. β_i and C_i denote the coefficients and the binary indicator of each cell type, respectively. n is the number of cell types. Since one cell type needs to be chosen as a reference for model fitting, scMAPA fits the model twice to get the estimates of coefficients for all cell types. V_j and β_j denote the sample-specific binary confounding variables (e.g., clinical variable) and their coefficients, respectively. m is the number of confounding factors.

When there is no confounding factor, the likelihood ratio test (LRT) between cell type only model and null model is conducted to test the unadjusted effect of cell type, which is equivalent to the likelihood ratio chi-squared test of independence between long/short isoforms and cell types. With the existence of confounding variables, LRT between the full model and confounding variables only model is conducted to test the adjusted effect of cell type. P-values from all tests are further adjusted by the Benjamini–Hochberg procedure to control the false-discovery rate (FDR) at 5%. In addition, to ensure there is a significant change in effect size, the odds ratio of each cell type against the grand mean of all included cell types is calculated. There should be at least one cell type whose odds ratio is greater than 0.25 for a gene to be called an APA gene.

Currently, scMAPA assumes only 2 pA sites in the 3′-UTRs. However, our logistic model for step 2 can be easily extended to detect >2 peaks if employing other quantifiers that can consider >2 pA sites. For example, when only 2 peaks are detected for a gene, a binary logistic regression model would be fitted. However, when more than 2 peaks are detected for a gene, a multinomial logistic regression model would be fitted. To the best of our knowledge, since the only current tool that detects >2 peaks is scAPA, a multinomial logistic regression model is only compatible with the peak detection result of scAPA. LRT test is used to estimate the significance of APA among multiple peaks and cell types similarly.

Identification of cluster-specific 3′-UTR dynamics.

For the genes where significant APA dynamics is detected, scMAPA further analyses which cell type significantly contributes to the APA in which direction within each gene. By using a weighted effect coding scheme, each coefficient in the logistic regression can be interpreted as a measurement of deviation from the grand mean of all cells. This grand mean is not the mean of

all cell type means, rather it is the estimate of the proportion of long isoforms of all cells for each gene. So, the unbalanced cell population sizes, which are common in scRNA-Seq would not affect the accuracy of estimation.

We use the following two criteria to determine the cluster-specific significant 3′-UTR dynamics:

First, given coefficients estimated from logistic regression, we use the Wald test to determine the p-value of each coefficient. P-values among all genes with significant APA of the same cell type are further adjusted by FDR. Then, the absolute coefficient must be greater than $\ln(2)$, corresponding to a 2-fold change in odds ratio. $coefficient \geq \ln(2)$ would be considered as 3′-UTR lengthening and $coefficient \leq -\ln(2)$ would be considered as 3′-UTR shortening. However, users can define a different cutoff value than $\ln(2)$ for $coefficient$ for the stringency they want to set on the identification.

Identification of genes of more than two pA sites.

scMAPA can be easily extended to detect more than two pA sites and subsequently identify the significant differential usage of them. To detect more than two pA sites, scMAPA employs a similar approach to DaPars as follows. Instead of optimizing the regression model with a fixed number of predictors (proximal and distal pA sites), the case with more than 2 pA sites across n cell types can be formulated as follows.

$$\begin{bmatrix} r_{11} & r_{12} & \cdots & r_{1n} \\ r_{21} & r_{22} & \cdots & r_{2n} \\ \vdots & \vdots & \cdots & \vdots \\ r_{m1} & r_{m2} & \cdots & r_{mn} \end{bmatrix} = \begin{bmatrix} 1 & 1 & \cdots & 1 \\ 0 & 1 & \cdots & 1 \\ \vdots & \vdots & \cdots & \vdots \\ 0 & 0 & \cdots & 1 \end{bmatrix}_{m\times m} \begin{bmatrix} w_{11} & \cdots & w_{1n} \\ w_{21} & \cdots & w_{21} \\ \vdots & \cdots & \vdots \\ w_{m1} & \cdots & w_{mn} \end{bmatrix}_{m\times n}$$

where m is the length of the longest 3′-UTR of a transcript. w_{ij} is the estimated abundance of one possible 3′-UTR i in cell type j. Then, detecting multiple PA sites and estimating the

abundance can be optimized by a LASSO regularization, in which the following equation should be optimized.

$$\underset{W}{\operatorname{argmin}} \frac{1}{2} || C - MW ||_2^2 + \lambda \|W\|_1$$

While the number of non-zero w_{ij} indicates the number of pA sites for this gene, scMAPA will consider the genes with up to four estimated non-zero w_{ij} by default that can be further changed by the user. While this would avoid overfitting, we expect the default value to allow us to capture most genes according to a recent study on the number of pA sites for genes[28].

After PA sites detection, the binomial logistic regression could be extended to a multinomial logistic regression to identify differential PA site usage when more than 2 PA sites exist. If in total P PA sites are detected by PA site detection module, the differential PA sites identification could be modeled as following,

$$\mathrm{Prob}(\mathrm{PA}_i = p) = \frac{e^{\beta_p} \cdot X_i}{\sum_{k=1}^{P} e^{\beta_k} \cdot X_i}$$

where p is one of the P PA sites. X_i is a row vector of features of an observed transcript. β_p is the coefficients *associated with PA site p*.

Simulation

First, we used Splatter[29], a widely known scRNA-Seq simulator, to simulate the cell-level count matrix, which acts as the base of synthetic data. Splatter was trained by unfiltered mouse brain data and set to generate count matrices containing 5000 genes and 3000 cells. The matrix

then collapsed into 5 columns, representing the total count of 5 cell groups. We call this 5000 × 5 matrix a cluster-level count matrix.

From the analyses of PBMC and mouse brain data, we found that the standard deviation of PDUI (percentage of distal polyA site usage, which is equivalent to the proportion of long isoforms) of each gene could act as a classifier of APA gene and non-APA gene. Based on that, the standard deviation of PDUI for APA genes in synthetic data is estimated by calculating the mean of standard deviations of PDUI from APA genes detected by both scMAPA and scAPA from mouse brain data. Similarly, the standard deviation of PDUI for non-APA genes was estimated by calculating the mean of standard deviations of PDUI from genes identified as non-APA by both scMAPA and scAPA. With the estimated standard deviations, a PDUI matrix with the same size (5000 × 5) as the cluster-level count matrices was generated. Each row of the PDUI matrix has a standard deviation equal to either the estimated standard deviation for the APA gene or the non-APA gene. This is achieved by centering 5 randomly selected numbers from standard normal distribution to 0. Then multiply the desired standard deviation to these centered numbers and add them to the desired mean. The mean of each row was randomly picked from 0.05 to 0.95. Since the estimated $SD_{isoprop}$ values are averaged to 0.127 and 0.009 for the APA and the non-APA genes respectively, we generated simulation data with $SD_{isoprop}$ for APA genes in a range centered on 0.13 while fixing that for non-APAs at 0.009. The rows representing true APA genes were randomly selected. Then, each number in the cluster-level count matrix is divided into the count of long isoforms and the count of short isoforms by multiplying and PDUI matrix or (1-PDUI matrix), respectively. Finally, Pearson's chi-squared test (scAPA), logistic regression model + LRT (scMAPA) could be applied to assess the performance of these three methods. For each repeat of simulation, PDUI matrix is regenerated but the cluster-level count matrix keeps

the same for the sake of computational burden. Every simulation design was repeated 100 times to derive summarized statistics.

To examine the impact of experimental design on statistical power to detect significant APA genes, we assess the performance of scMAPA and scAPA in the following aspects: 1) To test the impact of unbalanced cell populations, the proportion of 5 cell types in the synthetic cell-level count matrices were set to three scenarios with different distribution of cell-type populations: (20%, 20%, 20%, 20%, 20%), (30%, 17.5%, 17.5%, 17.5%, 17.5%), and (50%, 12.5%, 12.5%, 12.5%, 12.5%). 2) To test the impact of the proportion of true APA genes, we set three levels of true APA proportions, 5%, 10%, and 20%. 3) To test the impact of the extent of APA dynamics, instead of using mean of standard deviations, we set the standard deviations of true APA genes in the simulated PDUI matrix to the 15 equally spaced sequence of numbers between the first quartile and the third quartile of standard deviations estimated from APA genes in mouse brain data. In total, there were 9 scenarios, corresponding to 9 combinations of factors 1) and 2). When testing factor 3), we chose balanced cell type proportion (0.2, 0.2, 0.2, 0.2, 0.2) and 10% true APA genes.

Assessing accuracy of PA site estimation

To assess the PA site/ peak interval prediction accuracy, we used peak lists or PA site lists from scMAPA, scAPA, and Sierra on PBMC data. The estimation accuracy is measured by the percentage of the predicted peaks or PA sites overlapped with PA sites annotated in PolyASite 2.0. Since it is meaningless to find the overlap between two-point estimates, we expanded the point position from the annotation database to an interval by manually adding a distance ranging from 10 bp to 150 bp in a 10 bp increment to both sides of the annotated PA sites. scMAPA gives a point estimate of PA site as predicted proximal PA site and Sierra gives two-point

estimates as fit max position and max position. To make the comparison more comprehensive, we calculated the midpoint of peak interval as the pseudo point estimate of scAPA. The point estimates from these methods are considered as supported by the annotation database if the point position falls in the annotated interval (annotated PA site ± distance). For peak intervals estimated by scAPA and Sierra, as long as there is 1 bp overlap between the estimated interval and the annotated interval (either start or end of estimated interval falls in annotated PA site ± distance), the estimate would be considered as supported by annotation database. Then, the percentage supported by annotation is calculated as the number of PA sites or peak intervals supported by the annotation database divided by total peaks detected for each method.

Running scDAPA, scAPA and Sierra

Sierra and scDAPA were run with default parameters. scAPA was run with default parameters and intronic regions omitted. The genes with a CPM of less than 10 were filtered out. We want to point out that scAPA employs chisq.test function in R to estimate the significance of dynamic PA sites usage among multiple clusters. This potentially makes the identification of scAPA much conservative than other tools in the multi-group setting since it does not allow any cell type to have 0 count, as R's chisq.test would return NA as p-value if there is 0 presented in the count table. However, it is common to observe that a few cell types would not express certain genes in scRNA-Seq, especially when the whole cell population is split into more than 5 clusters (cell types), which is typical for complex biological systems.

To compare scDAPA and Sierra with scAPA and scMAPA in multiple-cluster settings, since scDAPA and Sierra identify APA genes only between cell cluster pairs, we combined the

pairwise significant APA genes in each method separately. After controlling FDR on the combined APA genes, we called APA genes if they are significant in any of the pairwise identifications.

Controlling confounders

To compare the running modes, we first divided the mouse brain data into 10 cell groups by cell type and brain region (5 cell types × 2 brain regions). In each data, we quantified the APA isoforms using scMAPA in two running modes, referred to as brain-region-confounding/controlled in the main text. The brain-region-confounding model is formulated as

$APA\_Isoform \sim cell\_type$.

And the brain-region-controlled model is formulated as

$APA\_Isoform \sim cell\_type$+brain_region.

Availability of supporting source code and requirements

Project name: scMAPA

Project home page: https://github.com/ybai3/scMAPA

Operating system: Platform independent

Programming language: R

License: GNU GPL

DECLARATIONS

Competing interests The authors declare no competing financial interests.

Funding This work was supported partly by the Joan Gollin Gaines Cancer Research Fund at the University of Pittsburgh to H.J.P.. This project used the UPMC Hillman Cancer Center Biostatistics Shared Resource that is supported in part by award P30CA047904.

Author Contributions H.J.P and Y.B. conceived the project, designed the experiments. Y.B. and Z.F. implemented the software. Y.B., Y.Q., R.M. performed the analysis. S.K., K.N., H.M.Z., R.K., Q.P. interpreted the results statistically and/or biologically.

Acknowledgements We thank Daniel Weeks, Ph.D., Professor, Department of Human Genetics, University of Pittsburgh for valuable discussion. This research was supported in part by the University of Pittsburgh Center for Research Computing through the resources provided. We also acknowledge the authors of scAPA for their generous provision of their data.

REFERENCES

[1] A. Derti *et al.*, "A quantitative atlas of polyadenylation in five mammals.," *Genome Res.*, vol. 22, no. 6, pp. 1173–1183, Jun. 2012, doi: 10.1101/gr.132563.111.

[2] C. P. Masamha *et al.*, "CFIm25 links alternative polyadenylation to glioblastoma tumour suppression.," *Nature*, vol. 510, no. 7505, pp. 412–416, May 2014, doi: 10.1038/nature13261.

[3] L. C. Cheng *et al.*, "Widespread transcript shortening through alternative polyadenylation in secretory cell differentiation," *Nat. Commun.*, vol. 11, no. 1, p. 3182, 2020, doi: 10.1038/s41467-020-16959-2.

[4] M. Chen *et al.*, “3 ′ UTR lengthening as a novel mechanism in regulating cellular senescence,” *Genome Res.*, vol. 28, no. 3, pp. 285–294, 2018, doi: 10.1101/gr.224451.117.Freely.

[5] C. Ye *et al.*, “scDAPA: detection and visualization of dynamic alternative polyadenylation from single cell RNA-seq data,” *Bioinformatics*, vol. 36, no. 4, pp. 1262–1264, 2020, doi: 10.1093/bioinformatics/btz701.

[6] R. Patrick *et al.*, “Sierra: discovery of differential transcript usage from polyA-captured single-cell RNA-seq data,” *Genome Biol.*, vol. 21, no. 1, p. 167, 2020, doi: 10.1186/s13059-020-02071-7.

[7] E. D. Shulman and R. Elkon, “Cell-type-specific analysis of alternative polyadenylation using single-cell transcriptomics data,” *Nucleic Acids Res.*, vol. 47, no. 19, pp. 10027–10039, 2019, doi: 10.1093/nar/gkz781.

[8] S. Heinz *et al.*, “Simple combinations of lineage-determining transcription factors prime cis-regulatory elements required for macrophage and B cell identities,” *Mol. Cell*, vol. 38, no. 4, pp. 576–589, May 2010, doi: 10.1016/j.molcel.2010.05.004.

[9] A. H. Shih *et al.*, “Mutational Cooperativity Linked to Combinatorial Epigenetic Gain of Function in Acute Myeloid Leukemia,” *Cancer Cell*, vol. 27, no. 4, pp. 502–515, 2015, doi: https://doi.org/10.1016/j.ccell.2015.03.009.

[10] M. Jung *et al.*, “GATA2 deficiency and human hematopoietic development modeled using induced pluripotent stem cells.,” *Blood Adv.*, vol. 2, no. 23, pp. 3553–3565, Dec. 2018, doi: 10.1182/bloodadvances.2018017137.

[11] R. Bohnert and G. Rätsch, “rQuant.web: a tool for RNA-Seq-based transcript quantitation.,” *Nucleic Acids Res.*, vol. 38, no. Web Server issue, pp. W348-51, Jul. 2010, doi: 10.1093/nar/gkq448.

[12] L. Li *et al.*, “An atlas of alternative polyadenylation quantitative trait loci contributing to complex trait and disease heritability,” *Nat. Genet.*, vol. 53, no. July, 2021, doi: 10.1038/s41588-021-00864-5.

[13] A. Zeisel *et al.*, “Molecular Architecture of the Mouse Nervous System.,” *Cell*, vol. 174, no. 4, pp. 999-1014.e22, Aug. 2018, doi: 10.1016/j.cell.2018.06.021.

[14] T. Stuart *et al.*, “Comprehensive Integration of Single-Cell Data,” *Cell*, vol. 177, no. 7, pp. 1888-1902.e21, Jun. 2019, doi: 10.1016/j.cell.2019.05.031.

[15] X. Zhang *et al.*, “CellMarker: a manually curated resource of cell markers in human and mouse,” *Nucleic Acids Res.*, vol. 47, no. D1, pp. D721–D728, Jan. 2019, doi: 10.1093/nar/gky900.

[16] C. J. Herrmann, R. Schmidt, A. Kanitz, P. Artimo, A. J. Gruber, and M. Zavolan, “PolyASite 2.0: a consolidated atlas of polyadenylation sites from 3′ end sequencing,” *Nucleic Acids Res.*, vol. 48, no. D1, pp. D174–D179, Jan. 2020, doi: 10.1093/nar/gkz918.

[17] C. Ye, Q. Zhou, Y. Hong, and Q. Q. Li, “Role of alternative polyadenylation dynamics in acute myeloid leukaemia at single-cell resolution.,” *RNA Biol.*, vol. 16, no. 6, pp. 785–797, Jun. 2019, doi: 10.1080/15476286.2019.1586139.

[18] Y. Xiang *et al.*, “Comprehensive Characterization of Alternative Polyadenylation in Human Cancer,” vol. 110, no. November 2017, pp. 1–11, 2018, doi: 10.1093/jnci/djx223.

[19] Z. Xia *et al.*, “Dynamic Analyses of Alternative Polyadenylation from RNA- Seq Reveal Landscape of 3 ’ UTR Usage Across 7 Tumor Types,” *Nat. Commun.*, pp. 1–38, 2014.

[20] P. J. Shepard, E.-A. Choi, J. Lu, L. A. Flanagan, K. J. Hertel, and Y. Shi, “Complex and dynamic landscape of RNA polyadenylation revealed by PAS-Seq.,” *RNA*, vol. 17, no. 4, pp. 761–772, Apr. 2011, doi: 10.1261/rna.2581711.

[21] V. Hilgers, M. W. Perry, D. Hendrix, A. Stark, M. Levine, and B. Haley, “Neural-specific elongation of 3’ UTRs during Drosophila development.,” *Proc. Natl. Acad. Sci. U. S. A.*, vol. 108, no. 38, pp. 15864–15869, Sep. 2011, doi: 10.1073/pnas.1112672108.

[22] Z. Ji, J. Y. Lee, Z. Pan, B. Jiang, and B. Tian, “Progressive lengthening of 3’ untranslated regions of mRNAs by alternative polyadenylation during mouse embryonic development.,” *Proc. Natl. Acad. Sci. U. S. A.*, vol. 106, no. 17, pp. 7028–7033, Apr. 2009, doi: 10.1073/pnas.0900028106.

[23] A. Feiglin, B. K. Allen, I. S. Kohane, and S. W. Kong, “Comprehensive Analysis of Tissue-wide Gene Expression and Phenotype Data Reveals Tissues Affected in Rare Genetic Disorders,” *Cell Syst.*, vol. 5, no. 2, pp. 140-148.e2, 2017, doi: 10.1016/j.cels.2017.06.016.

[24] Y. Hao *et al.*, “Integrated analysis of multimodal single-cell data.,” *Cell*, vol. 184, no. 13, pp. 3573-3587.e29, Jun. 2021, doi: 10.1016/j.cell.2021.04.048.

[25] T. Smith, A. Heger, and I. Sudbery, “UMI-tools: Modeling sequencing errors in Unique Molecular Identifiers to improve quantification accuracy,” *Genome Res.*, vol. 27, no. 3, pp. 491–499, 2017, doi: 10.1101/gr.209601.116.

[26] M. V. Kuleshov *et al.*, “Enrichr: a comprehensive gene set enrichment analysis web server 2016 update,” *Nucleic Acids Res.*, vol. 44, no. W1, pp. W90–W97, 2016, doi: 10.1093/nar/gkw377.

[27] A. R. Quinlan, *BEDTools: The Swiss-Army tool for genome feature analysis*, vol. 2014. 2014.

[28] B. J. Harrison *et al.*, “Detection of Differentially Expressed Cleavage Site Intervals Within 3′ Untranslated Regions Using CSI-UTR Reveals Regulated Interaction Motifs ,” *Frontiers in Genetics* , vol. 10. p. 182, 2019, [Online]. Available: https://www.frontiersin.org/article/10.3389/fgene.2019.00182.

[29] L. Zappia, B. Phipson, and A. Oshlack, “Splatter: simulation of single-cell RNA sequencing data,” *Genome Biol.*, vol. 18, no. 1, p. 174, 2017, doi: 10.1186/s13059-017-1305-0.

[30] C. Ye, Y. Long, G. Ji, Q. Q. Li, and X. Wu, “APAtrap: identification and quantification of alternative polyadenylation sites from RNA-seq data,” doi: 10.1093/bioinformatics/bty029.

[31] A. Arefeen, J. Liu, X. Xiao, and T. Jiang, “TAPAS : tool for alternative polyadenylation site analysis,” *Bioinformatics*, vol. 34, no. February, pp. 2521–2529, 2018, doi: 10.1093/bioinformatics/bty110.

[32] S. Picelli, O. R. Faridani, A. K. Björklund, G. Winberg, S. Sagasser, and R. Sandberg, “Full-length RNA-seq from single cells using Smart-seq2.,” *Nat. Protoc.*, vol. 9, no. 1, pp. 171–181, Jan. 2014, doi: 10.1038/nprot.2014.006.

S. Table 1. Cell type annotation based on marker genes curated in CellMarker[20] for 10k, 5k, and 1k in the PBMC data.

S. Table 2. Detailed information of APA genes detected by scMAPA, scAPA, scDAPA, and Sierra on the PBMC data including Ingenuity Pathway Analysis (IPA) analysis result.

S. Table 3. scMAPA estimation result for APA genes on the mouse brain data.

S. Table 4. Result of IPA comparison analysis on the "Disease & Function" terms enriched for APA genes identified uniquely by scAPA, scMAPA, and commonly by both on the mouse brain data (1,446, 2,175, and 1,048 respectively).

S. Table 5. Result of IPA comparison analysis on the "Disease & Function" terms enriched for APA genes identified uniquely in astrocyte, immune, oligos, vascular, and neuron cells.

S. Table 6. scMAPA estimates on the input data that are split by cell type and brain region either with brain region as a confounder or not.

S. Table 7. IPA upstream regulator analysis result (enrichment p-value) on 113 and 2,715 APA genes that are supposed to be brain-region-specific and non-specific, respectively.

 Click here to access/download;Figure;Fig1.docx

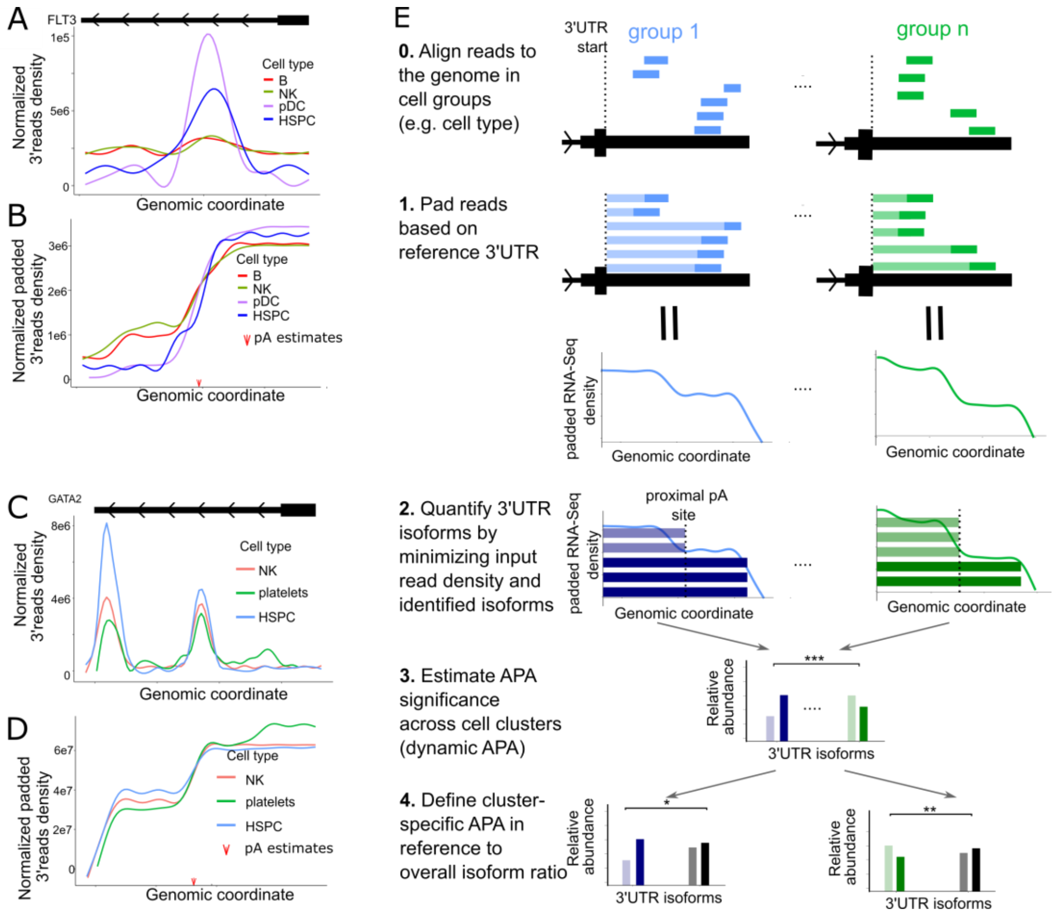

Figure 1. Motivation and schematic illustration of scMAPA. (A) The read density shape on the FLT3 3′-UTR in multiple cell types of 10k PBMC scRNA-Seq data. (B) The transformed read density shape on the FLT3 3′-UTR in multiple cell types of 10k PBMC scRNA-Seq data. The red arrow indicates the proximal polyA site predicted. (C) The read density shape on the GATA2 3′-UTR in multiple cell types of 10k PBMC scRNA-Seq data. (D) The transformed read density shape on the GATA2 3′-UTR in multiple cell types of 10k PBMC scRNA-Seq data. The red arrow indicates the proximal polyA site predicted. (E) In Step 0 and 1, bars in solid color represent 3′ biased scRNA-Seq reads and bars in light color indicate how the 3′ biased reads are padded from the 3′ start site to the end of the read to represent the full-length 3′ UTR of the transcript. In Step 2, the blue and green bars indicate the estimated isoforms in each cell type, where solid and light coloring mode indicate 3′ UTR long and short isoforms. In Step 3 and 4, the bars represent the estimated number of APA isoforms in each cell type.

Figure
Click here to access/download;Figure;Fig2.docx

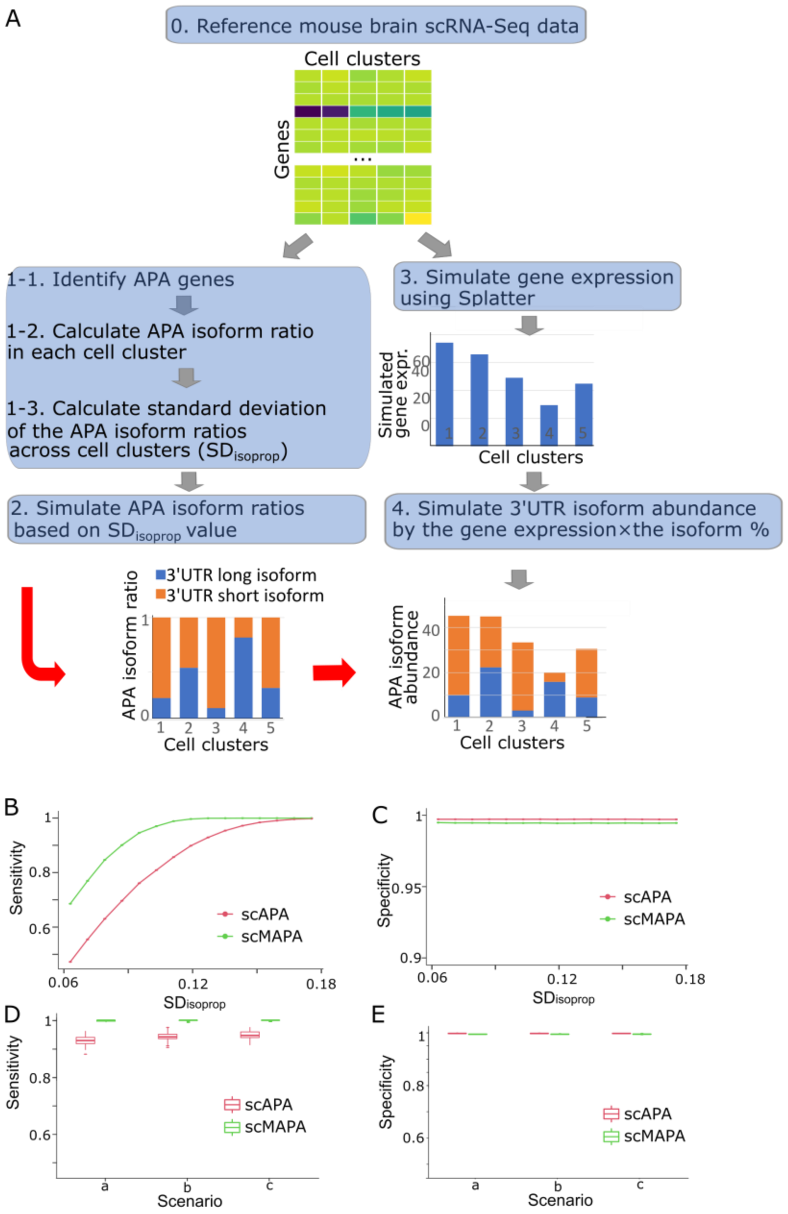

Figure 2. Performance assessment on the statistical component of scMAPA and scAPA using simulated data. With fixed number of true APA events (500 out of 5000) and uniform distribution of cell cluster size (600 cells in each cell type) (A). Illustration of the simulation process. Genes identified as significant APA genes by both scMAPA and scAPA were considered as APA genes. Genes identified as non-significant APA genes by both methods were considered as non-APA genes. (B) Comparison of scMAPA vs. scAPA in terms of sensitivity. We varied the standard deviation (SD) of APA isoforms across clusters ($SD_{isoprop}$) for 500 true APA genes (0.06 to 0.18) with the fixed $SD_{isoprop}$ value for 4,500 non-APA genes (0.009). (C) Comparison of scMAPA vs. scAPA in terms of specificity in the same scenario. (D) Comparison of scMAPA vs. scAPA in terms of sensitivity. We varied cell cluster size: (20%, 20%, 20%, 20%, 20%) for scenario a, (30%, 17.5%, 17.5%, 17.5%, 17.5%) for b, and (50%, 12.5%, 12.5%, 12.5%, 12.5%) for c.

Figure Click here to access/download;Figure;Fig3.docx

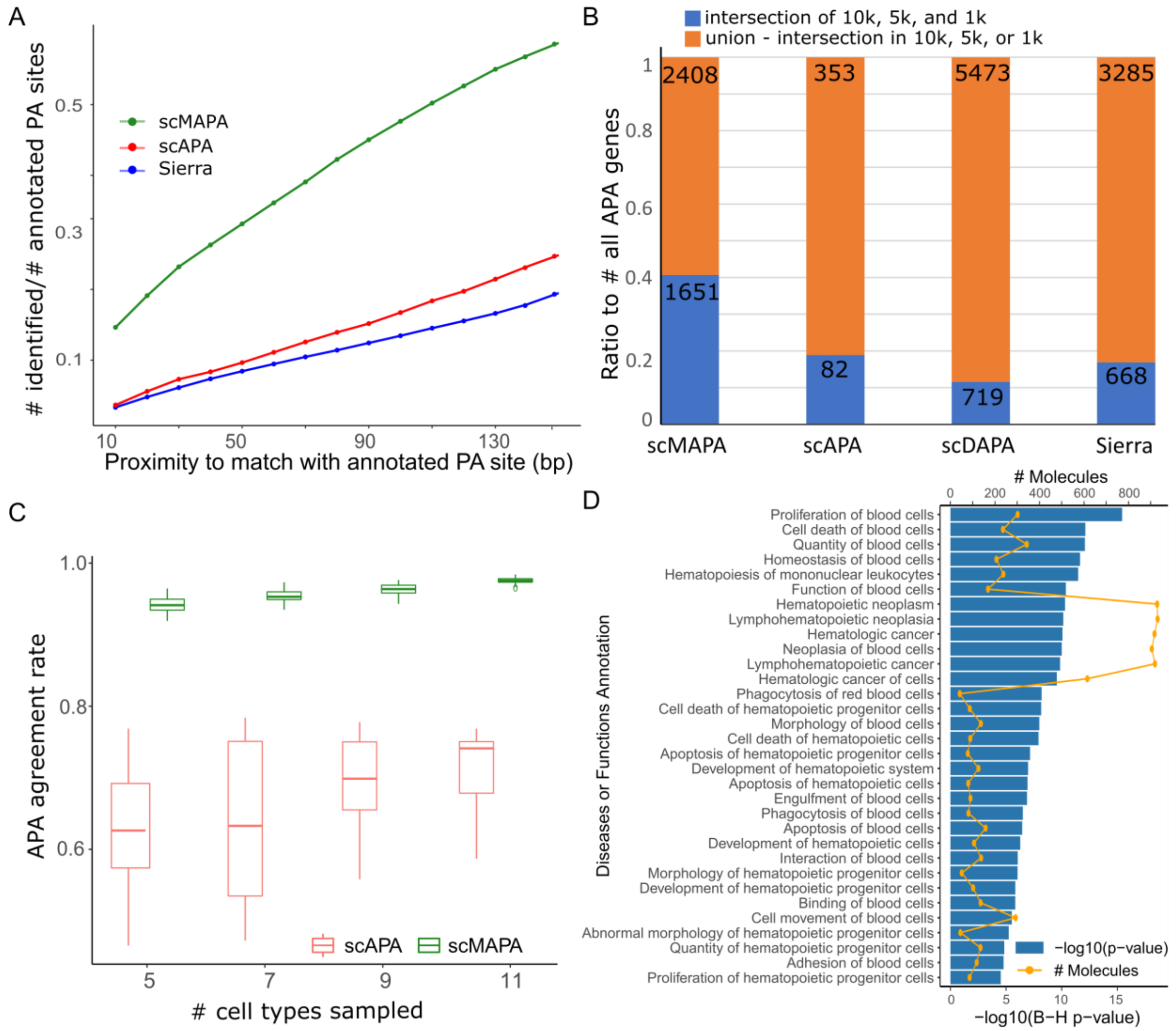

Figure 3. Performance assessment of scMAPA, scAPA, scDAPA, and Sierra using PBMC data. (A) The ratio of annotated pA sites identified by scMAPA vs. scAPA and Sierra on the PBMC 10k data. The identified pA sites were deemed annotated when they are within a range to any annotated pA sites while the range was set from 10 bp to 130 bp, respectively. We extracted the annotated pA sites from PolyASite 2.0. (B) The ratio of significant APA genes found in all three PBMC data (10k, 5k, and 1k) in blue bar and in any combination but all three in orange by scMAPA, scAPA, scDAPA, and Sierra (C) Box plots showing the proportion of the overlap between sample APA genes and total APA genes normalized to total APA genes (APA agreement ratio). The APA agreement ratio values were evaluated in various numbers of cell types sampled. (D) Significance of enrichment (blue bar) and number of overlaps (orange line) of 3,574 scMAPA APA genes on IPA Disease and Function terms with the keyword “blood” or “hematopoiesis”.

Figure Click here to access/download;Figure;Fig4.docx

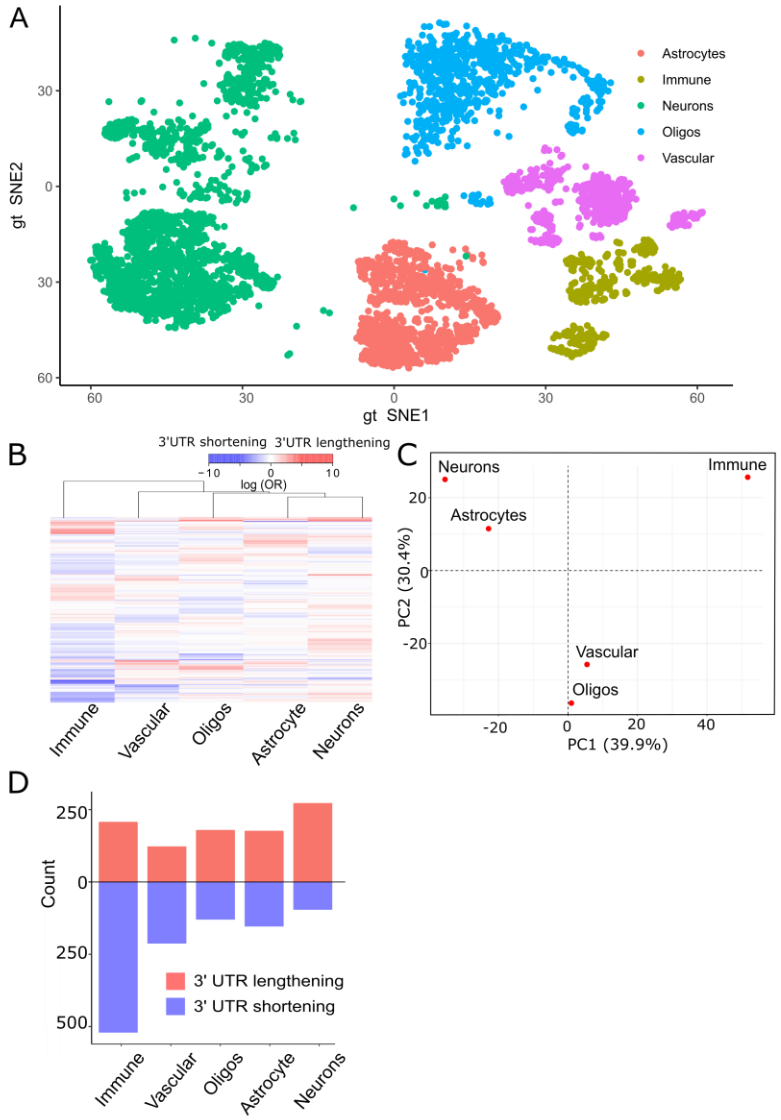

Figure 4. A novel module of scMAPA cell-type-specific APA identification on the mouse brain data. (A) tSNE plot showing the cell types of the mouse brain scRNA-Seq data. (B) Heatmap of the APA effect sizes estimated for each cell type, representing the coefficients in the scMAPA logistic regression model. (C) PCA plot showing how the cell types are similar or dissimilar in the APA effect size. PC1 and PC2 together account for 70.3% of the variation. (D) Bar plot showing the number of significant 3′-UTR lengthening (red) and shortening (blue) identified in each cell type.

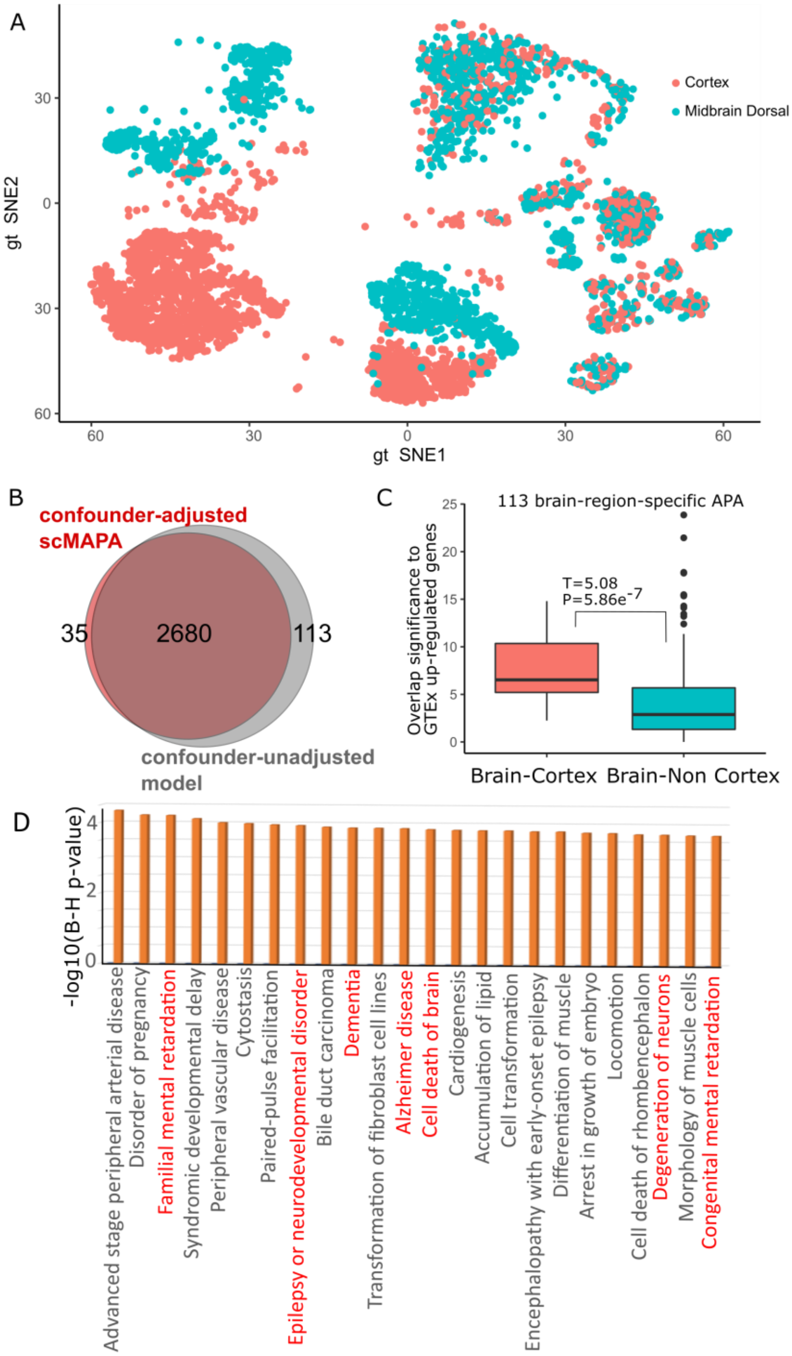

Figure 5. (A) tSNE plot showing the brain region of the mouse brain scRNA-Seq data. (B) Venn diagram showing the APA genes identified by the confounder-adjusted scMAPA and the confounder-unadjusted model. (C) Box plot showing significance of overlap between the 113 genes and the up-regulated genes in GTEx brain samples whether they are from cortex (red) or not (green). (D) Significance (B-H p-value) of IPA enrichment terms that are uniquely and significantly (B-H p-value$<10^{-2}$) enriched to 2,793 confounder-adjusted scMAPA.

Click here to access/download
Supplementary Material
ver24_SFigures.docx

Supplementary Table 1

Click here to access/download
Supplementary Material
STable1_Supplementary Material.xlsx

Supplementary Table 2

Click here to access/download
Supplementary Material
STable2_Supplementary Material.xlsx

Supplementary Table 3

Click here to access/download
Supplementary Material
STable3_Supplementary Material.xlsx

Supplementary Table 4

Click here to access/download
Supplementary Material
STable4_Supplementary Material.xlsx

Supplementary Table 5

Click here to access/download
Supplementary Material
STable5_Supplementary Material.xlsx

Supplementary Table 6

Click here to access/download

Supplementary Material

STable6_Supplementary Material.xlsx

Supplementary Table 7

Click here to access/download
Supplementary Material
STable7_Supplementary Material.xlsx

Cover letter
Click here to access/download;Personal Cover;cover_letter_GigaScience.doc

University of Pittsburgh

Department of Human Genetics

Graduate School of Public Health
A300 Crabtree Hall
130 DeSoto Street
Pittsburgh, PA 15261
412-624-3018
Fax: 412-624-3020

Aug. 6th, 2021

Dear Dr. Zauner,

I would like to submit our manuscript, "**scMAPA: Identification of Cell-type-specific Alternative Polyadenylation in Complex Tissues**" for publication. This is a revision to our previous submission (GIGA-D-21-00066, "**Cell-type-specific alternative polyadenylation (APA) genes reveal the function of dynamic APA in complex tissues"**). Please find our point-to-point response to reviewers' comments in the next page.

Alternative polyadenylation (APA) is emerging as an important regulatory layer in transcriptomic analysis. Recently, several bioinformatics tools have been developed to identify genes with dynamic APA in single-cell RNA-Seq data. However, the current methods lack statistical power and interpretability for complex tissues.

To address these limitations, we developed scMAPA that systematically integrates two novel steps. First, it quantifies 3′-UTR long and short isoforms without posing assumptions on the signal shape of input data, increasing sensitivity in identifying APA genes. Second, it estimates the significance of the APA genes for each cell type while controlling confounders, enabling to study APA function in complex biological systems. Using our novel simulated and biological data, we showed that scMAPA outperforms the other methods and demonstrated how the increased power and enhanced interpretability would help better understand the APA biology in complex tissues.

We would like to ask to exclude the following researchers who are in competition to develop similar methods to review our manuscript.

- Dr. Wei Li at UCI (wei.li@uci.edu)
- Dr. Congting Ye at Xianmen University (yec@xmu.edu.cn)
- Dr. Xiaohui Wu at Xianmen University (xhuister@xmu.edu.cn)
- Dr. Ran Elkon at Tel Aviv University (ranel@tauex.tau.ac.il)
- Dr. Eldad David Shulman at Tel Aviv University (eldadshulman@mail.tau.ac.il)
- Dr. Richard P. Harvey at UNSW Australia (r.harvey@victorchang.edu.au)
- Dr. Kitty K. Lo at UNSW Australia (kitty.lo@sydney.edu.au)

Best Regards,

Hyun Jung Park, Ph.D.
HYP15@pitt.edu
http://parklab.pitt.edu

Summary: We would like to appreciate that Reviewers acknowledged the significance and novelty of our findings. For example:

- "*a **novel tool** for the identification*" (Reviewer #1)
- "*scMAPA, **improves on existing methods***" (Reviewer #1)
- "*this work **should be of use to researchers** interested in APA studies*" (Reviewer #2)
- "***The work was well carried out***" (Reviewer #2)

Please see below for a detailed **point-by-point response**. The comments of editor or reviewers are indicated in red font.

Response to Editor

Thank you for considering GigaScience. Peer review of your manuscript is now complete and, in the light of the reports, and my own assessment as Editor, I regret to inform you that your manuscript cannot be accepted for publication in GigaScience in its present form. Please find the reviewers' reports at the end of this email.

- Reviewer 2 would like to see some additional validation, e.g. including scDAPA and Sierra in the performance assessment, among other points.

Response:

Our performance assessment used simulated and biological (PBMC) data. When using the simulated data, we could not include scDAPA and Sierra because they do not meet two assumptions underlying the use of the simulated data. First, our simulation assumes five simulated cell clusters because $SD_{isoprop}$ determines the APA isoform ratios across the five simulated cell clusters. However, both scDAPA and Sierra are not designed to handle multiple cell clusters. For example, Sierra modified DEXSeq[1] such that it does not apply to more than 2 conditions (which is cell types in this case). Second, our simulation assumes that the method identifies APA events based on the expression levels of APA isoforms. For example, scAPA and scMAPA use Pearson's $\chi2$ and likelihood ratio test on the identified APA isoform abundances, respectively. However, scDAPA and Sierra do not separate the task of identifying APA isoform abundances and of calling APA events based on the abundances.

However, on the PBMC data, we included scDAPA and Sierra. Including scDAPA and Sierra was possible since the real data are not restricted by $SD_{isoprop}$. Since it is not restricted by $SD_{isoprop}$, we collected the scDAPA and Sierra results from all pairwise identifications after FDR control (see Methods).

- Reviewer 1 also has a number of critical concerns that require more work. The reviewer seems to have missed the link to the "supporting methods", which was included in the PDF. However, the reviewer is correct that the methods section should be part of the main paper.

Response: We apologize that we did not put the methods section in the main paper. In this submission, we ensured that all the sections are aligned in the proper order in the main paper.

The write-up of the manuscript also needs to be improved in terms of clarity.

Response: We greatly appreciate pointing out this important improvement. We updated our overall writing in the new submission.

Besides what the Editor pointed out; we addressed all the Reviewers' comments in the below.

Response to Reviewer #1

The authors here present a novel tool for the identification of alternatively polyadenylated (APA) genes from single cell RNA sequencing (scRNA-seq). The tool, scMAPA, improves on existing

methods by modelling the expression profile of APA sites without assuming data are peak-based. scMAPA can achieve this even for multi-cellular source data.

In the manuscript the authors make reference several times to a Methods section which does not appear to exist as well as multiple supplementary figures and tables which are also not available to this reviewer.

It also appears that this manuscript is not properly formatted for a Technical Note which requires Abstract, Keywords, Findings and Methods not Abstract, Introduction, Results and Discussion.

The authors need to follow the instructions for the appropriate manuscript type they wish to submit as currently this is not a Technical Note.

Given these deficiencies, I cannot review the submission properly, but give the following general guidance and comments.

* Given their use of a simulation, how have the authors ensured that the simulation is both representative and not unintentionally optimised to favour scMAPA?

Response:

To ensure that the simulated APA gene data are representative, we identified high variance of APA isoform ratios as a common feature of APA genes across multiple cell types in the manuscript as follows. “we decided to quantify a common feature of the APA genes by calculating the proportion of the long and short isoforms in each cell type and the standard deviation of the proportions across the five cell types ($SD_{isoprop}$, see Methods). To validate the effectiveness of this measure, we calculated $SD_{isoprop}$ values for non-APA genes that scAPA and scMAPA agreed on in the data. We found that high $SD_{isoprop}$ values significantly distinguish APA genes from non-APA genes (0.127 vs. 0.009 of $SD_{isoprop}$ on average, $p<2.2e^{-16}$, **S. Fig. 2A**), suggesting that it is reasonable to simulate APA genes to have high $SD_{isoprop}$ values in the data of multiple (≥2) cell types” With this, we simulated the APA genes to have a higher variance in the APA isoform (3′-UTR long and short) ratios than non-APA genes across the simulated cell clusters (step 2 in **Fig. 2A**).

To ensure that the simulation is not optimized to favour scMAPA, we set $SD_{isoprop}$ based on the APA and non-APA genes identified by both scMAPA and scAPA in the mouse brain data (Step 1 in **S. Fig. 2A**). This is because scMAPA and scAPA are the only methods that can identify APA genes in the multi-cluster setting (**S. Fig. 1C**). To conduct another important part of the simulation which is simulating gene expression levels (Step 3 in **S. Fig. 2A**), we ensured not to favor scMAPA by using an independent method, Splatter, and by taking the parameters from public biological data, the mouse brain data. Since the rest step (Step 4 in **S. Fig. 2A**) is a simple numeric calculation based on the simulated data above, no other bias is expected to favor scMAPA.

* How is SDisoprop is good parameter for discriminting between good and bad performance?

Response: $SD_{isoprop}$, defined as the standard deviation value of the APA isoform ratios across cell clusters, is a good parameter, because this measure captures the common characteristic of APA genes as shown above.

* The description of how IPA was used and how it supports the authors' conclusions is unclear. Could they do the same with GO?

Response: Thanks to your comments, I clarified the manuscript as follows; “we performed Ingenuity Pathway Analysis (IPA) on 3,574 APA genes that scMAPA identified in the 10k PBMC data. Especially, to accurately investigate the APA genes’ roles in PBMC biology, we set the 18,804 genes expressed in the data as the background.”

To clarify how this analysis supports our conclusion, we updated the description in the manuscript as follows, “This IPA analysis shows significant (B-H p-value < 10^{-2}) enrichments to 32 IPA terms that are characterized with keywords “blood” and “hematology” (**Fig. 3D**), suggesting that the APA genes identified by scMAPA can play important roles in PBMC biology.”

With the reviewer’s suggestion of doing the same with GO, we identified multiple GO terms significantly enriched with the 3,574 genes using gProfiler, including 3 GO terms with keyword “hematopoietic” (**R. Table 1**). The “hematopoietic” GO terms support our conclusion that scMAPA enables the identification of biologically reasonable APA genes in the PBMC scRNA-Seq data.

GO biological process	Total # genes	Observed # of overlaps	Expected # of overlaps	Over-/under-expressed	Fold enrichment	FDR p-value
regulation of hematopoietic progenitor cell differentiation (GO:1901532)	90	34	14.06	Over	2.42	2.21E-03
regulation of hematopoietic stem cell differentiation (GO:1902036)	75	27	11.72	Over	2.3	1.34E-02
hematopoietic or lymphoid organ development (GO:0048534)	619	141	96.69	Over	1.46	3.12E-03

R. Table 1. GO analysis on 3,574 APA genes identified by scMultiAPA.

* The use of the heatmap and dendrogram in Figure 4 to support "systematically confirms the previous finding" is not very convincing.

Response: We thank the reviewer for this suggestion. We agree with the reviewer that the current claim is not very convincing for two reasons. First, since the “previous finding” refers to the APA pattern difference between neuron and immune cell type, our previous claim that computational analyses “systematically confirm” the APA pattern differences might be too strong. Hence, we changed it into “the analyses supported the previous finding” in the manuscript. Second, the heatmap and dendrogram might not directly show the difference. To demonstrate the difference in a more direct way, we conducted additional analyses described as follows. “we performed the PCA analysis (**Fig. 4C**) and calculated Euclidean distance (**S. Fig. 4A**) among the cell types. While both the analyses supported the previous finding that immune and neuron cells are most different in terms of the APA effect size [26], they further revealed that immune cells are different from all other cell types.”

* The confounding factors section is very confusing and I am not sure what it is the authors are trying to highlight.

Response: We appreciate the reviewer’s feedback. In response to this reviewer’s comment, we extensively worked to improve the clarity of this section in the manuscript as follows. “To show how scMAPA controls confounding factors and why it is important, we analyzed the mouse brain data consisting of 5 cell types collected from 2 brain regions (cortex and midbrain). Since some cell types were collected from multiple brain regions (**Fig. 4A, 5A**), some APA genes associated with a brain region can be mistakenly identified as cell-type-specific APA genes, which would further confound studying cell-type-specific functions of APA genes. To see if scMAPA can remove such false positive APA genes, we ran scMAPA with the brain region information (cortex and midbrain dorsal) as the confounder (confounder-adjusted scMAPA) and without the

confounder (confounder-unadjusted model), separately. As the confounder-adjusted scMAPA and the confounder-unadjusted model identified 2,715 and 2,793 APA genes respectively (**S. Table 6**), 113 genes are no longer identified as significant APA gene after adjusting brain region. Thus, these APA genes are expected to be related to the confounders (confounder-related APA genes, **Fig. 5B**), which is the brain region it was sampled from (cortex and midbrain). To test if the 113 genes function specifically for the brain region, we test if they express highly specifically in the brain region. To conduct this test comprehensively, we identified their human homolog genes in the Mouse Genomic Informatics (MGI) homology database and compared expression of human homologs between cortex and other brain regions in the Genotype-Tissue Expression (GTEx) [2] (see Methods). The result shows that these APA genes are significantly up-regulated in brain cortex compared to other brain regions (p-value=$5.8e^{-7}$, **Fig. 5D**), suggesting that their functions are specific to brain cortex. Since GTEx does not collect the expression data for midbrain, we did not conduct this analysis for midbrain. This result suggests that, when scMAPA sets a confounder, it can successfully distinguish and exclude the APA genes that are likely related to the confounder.

To demonstrate why distinguishing and excluding the confounder-related APA genes is critical for accurate downstream analysis, we further conducted IPA analysis on the 2,715 and 2,793 APA genes identified by the confounder-adjusted scMAPA and the confounder-unadjusted model respectively (confounder-adjusted and confounder-unadjusted APA genes, respectively). Comparing the IPA enrichment between confounder-adjusted and confounder-unadjusted APA genes, we found considerable differences in important terms for brain study: among the 24 terms to which the confounder-adjusted APA genes are uniquely and significantly (B-H P-value $< 10^{-2}$) enriched, 7 terms are directly related to brain diseases (**Fig. 5D**). For example, two terms with the keyword "mental retardation" are significantly enriched (B-H P-value $< 10^{-3.5}$) only for the confounder-adjusted APA genes. On the other hand, among the 30 terms to which the confounder-unadjusted APA genes are uniquely and significantly enriched, no term refers to a brain disease (**S. Fig. 5A**). This result suggests that the confounder-adjusted scMAPA uncovers the APA genes that can play critical roles in the brain disease, which would be invisible due to the confounding factors."

Finally, with respect to the code on github, it is good there is a tutorial to follow with simple steps 1, 2 and 3. However, the implementation is impossible to follow without know the source data very well. In the reference data they link to (omitted here for simplicity), there are 132 different sample BAM files, none of which match the description of "Neurons, Immunes, and Oligos" as per the README. It is the same with the "Cluster information" file.
Where is the information for barcodes for each sample? I cannot, therefore, test the code for functionality. I recommend the authors make the tutorial much clearer making it absolutely clear what each step's inputs are and where they come from.
Response: We deeply appreciate your careful review. We revised our github page according to the reviewer's advice. To run scMAPA, we provide mb_example.bam and mb_cluster.csv as example data on our GitHub page, which is a downsized mouse brain scRNA data [3]. In the example data, we only keep three cell clusters: Neurons, Immune cells, and Oligos to make it easy to use. For fast processing, 200 cells are kept for each cluster. Additionally, we put example commands and configurations for each step on our Github page.

Thank you for your time and effort to review our manuscript. We appreciate your detailed comments. Please feel free to let us know if there are things to improve. We are looking forward to hearing your thoughts about our revised manuscript.

Response to Reviewer #2

This manuscript by Bai et al. reports a new computational method named scMAPA for APA analysis in single cells. The authors show the performance of scMAPA compared to other programs. They additionally revealed some interesting APA events in PBMC and brain cell data. Overall, this work should be of use to researchers interested in APA studies. The work was well carried out. There are, however, multiple issues that prevent the manuscript from being acceptable for publication at this stage.

Thank you for your acknowledgment of our method. Please see below for a detailed **point-by-point response**. The red fonts are your comment, and the black fonts are our response.

Major:

- Performance assessment should include scDAPA and Sierra.

Response: Our performance assessment used simulated and biological (PBMC) data. When using the simulated data, we could not include scDAPA and Sierra because they do not meet two assumptions underlying the use of the simulated data. First, our simulation assumes five simulated cell clusters because $SD_{isoprop}$ determines the APA isoform ratios across the five simulated cell clusters. However, both scDAPA and Sierra are not designed to handle multiple cell clusters. For example, Sierra modified DEXSeq[1] such that it does not apply to more than 2 conditions (which is cell types in this case). Second, our simulation assumes that the method identifies APA events based on the expression levels of APA isoforms. For example, scAPA and scMAPA use Pearson's $\chi 2$ and likelihood ratio test on the identified APA isoform abundances, respectively. However, scDAPA and Sierra do not separate the task of identifying APA isoform abundances and of calling APA events based on the abundances.

However, on the PBMC data, we included scDAPA and Sierra. Including scDAPA and Sierra was possible since the real data are not restricted by $SD_{isoprop}$. Since it is not restricted by $SD_{isoprop}$, we collected the scDAPA and Sierra results from all pairwise identifications after FDR control (see Methods).

Some false positive and false negative examples should be shown.

Thanks to the reviewer's comment, we inspected potential false positive and false negative examples of scMAPA identifications. First, among 62 significant APA events identified only by scMAPA in the 10k PBMC data, we found a potential false-positive gene in which the APA isoform ratios do not seem to vary much, PF4 (**R. Fig. 1A**). After our data transformation, the padded 3'UTR read density of PF4 shows limited variations across the cell types. Due to the limited variation, one may not suspect a significant APA event in PF4. An inspection of the pA estimate identified for PF4 also corroborates the possibility that it may be a false positive since the identified pA site does not show an apparent 'change-point' of the read coverage. Second, among 41 APA events identified by all the other methods (scAPA, Sierra, and scDAPA) but scMAPA, we found a potential false-negative gene in which the APA isoform ratios seem to vary, such as BCL9L. The padded 3'UTR read density of BCL9L shows various APA effect sizes especially in four cell clusters (B-2, CD16+ Mono, Platelets, and HSPC, **R. Fig. 1B, C**). However, since all the four clusters with a high APA effect size contain small sample sizes (**R. Fig. 1C**), it makes statistical sense not to estimate a significant APA event for BCL9L.

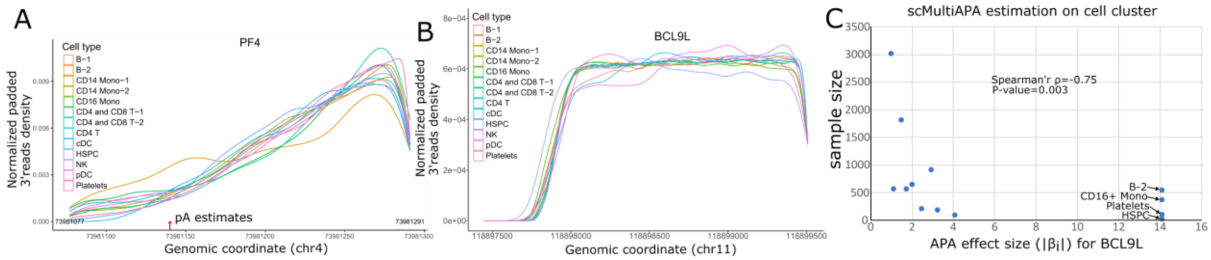

R. Figure 1 Normalized scRNA-Seq data density on the 3′-UTRs of PF4 and BCL9L (A, B, respectively). In A, the red arrow indicates the pA site identified by scMAPA. C. scMultiAPA estimated the APA degree (|βi| in scMultiAPA, see Methods) for each cell cluster for BCL9L, which is inversely correlated with the sample size.

- The authors should show explore false APA identification due to internal priming of A-rich internal regions.

Response: To explore false APA identification due to internal priming of A-rich internal regions, we implemented scMAPA to identify APA events potentially due to internal priming as written in Methods of the manuscript. "scMAPA can identify false APA identifications due to internal priming of A-rich internal regions if more than 7 consecutive adenines with up to 1 mismatch exists in 10 nt downstream of the predicted proximal PA site [4]. In the PBMC 10K data, we identified that 90 out of 3,574 APA events are due to suspected internal priming."

- The authors should show global 3'UTR size differences in PMBC cells as they did with brain cells.

Response: In response to the reviewer's suggestion, we showed global 3'UTR size differences in the 10k PMBC cells, which is now included in the supplementary material as follows. "The global size differences in PBMC cells are different from those in the mouse brain data in several aspects. First, 3'UTR lengthening occurs more than 3'UTR shortening in all the cell types (**S. Fig. 4E**). Second, however, the number of 3'UTR shortening genes is significantly correlated with that of lengthening genes across the cell types (P-value=$5e^{-5}$, **S. Fig. 4F**). Since both trends are not shown in the mouse brain data, scMAPA elucidates the unique APA profiles of the PBMC data."

- The writing and data presentation overall suffer from lack of clarity. Substantial editing is recommended to make this paper more readable.

Response: We thank the reviewer for suggesting this critical improvement. We updated our overall writing in the new submission.

Minor:

- In the Abstract, the authors indicate that they removed 'assumptions existing methods had…" The authors should make this more clear in the Abstract. I could not grasp what they are talking about.

Response: We updated that part in Abstract as follows "they assume certain read coverage shapes in the scRNA-Seq data, which can be violated in multiple APA genes."

- The authors should show some real examples in the main figure. For example, Figure 1 could be include examples like FLT3/GATA2 to illustrate their schematic.

Response: In response to the reviewer's suggestion, we moved the examples of FLT3/GATA2 into the main figure (**Fig. 1 A, B, C, D**).

- Fig. 3A. X-axis label "distance' is not clearly defined.

<u>Response:</u>
We updated the label into "proximity to match with annotated pA site (bp)" since we calculated the ratio of the identified pA sites that are close to the annotated pA sites.

Author's reference

[1] S. Anders, a Reyes, and W. Huber, "Detecting diferential usage of exons from RNA-seq data," *Genome Res*, vol. 22, no. 10, pp. 2008–2017, 2012, doi: 10.1101/gr.133744.111.

[2] A. Feiglin, B. K. Allen, I. S. Kohane, and S. W. Kong, "Comprehensive Analysis of Tissue-wide Gene Expression and Phenotype Data Reveals Tissues Affected in Rare Genetic Disorders," *Cell Syst.*, vol. 5, no. 2, pp. 140-148.e2, 2017, doi: 10.1016/j.cels.2017.06.016.

[3] A. Zeisel *et al.*, "Molecular Architecture of the Mouse Nervous System.," *Cell*, vol. 174, no. 4, pp. 999-1014.e22, Aug. 2018, doi: 10.1016/j.cell.2018.06.021.

[4] C. J. Herrmann, R. Schmidt, A. Kanitz, P. Artimo, A. J. Gruber, and M. Zavolan, "PolyASite 2.0: a consolidated atlas of polyadenylation sites from 3′ end sequencing," *Nucleic Acids Res.*, vol. 48, no. D1, pp. D174–D179, Jan. 2020, doi: 10.1093/nar/gkz918.
