# Supplementary material for: scMAPA: Identification of cell-type–specific alternative polyadenylation in complex tissues
Source: Gigascience. 2022 Apr 30;11:giac033. doi: 10.1093/gigascience/giac033 (PMC9055853; doi:10.1093/gigascience/giac033)
Supplement: giac033_GIGA-D-21-00240_Revision_1 [file giac033_giga-d-21-00240_revision_1.pdf]

GigaScience

scMAPA: Identification of Cell-type-specific Alternative Polyadenylation in Complex Tissues

--Manuscript Draft--

Manuscript Number:	GIGA-D-21-00240R1
Full Title:	scMAPA: Identification of Cell-type-specific Alternative Polyadenylation in Complex Tissues
Article Type:	Technical Note
Funding Information:	Joan Gollin Gaines Cancer Research Fund — Dr Hyun Jung Park<br>National Cancer Institute (P30CA047904) — Dr Hyun Jung Park
Abstract:	Alternative polyadenylation (APA) causes shortening or lengthening of the 3′-untranslated region (3′-UTR) of genes (APA genes) in diverse cellular processes such as cell proliferation and differentiation (cell-type-specific APA). To identify cell-type-specific APA genes in scRNA-Seq data, current bioinformatic methods have several limitations. First, they assume certain read coverage shapes in the scRNA-Seq data, which can be violated in multiple APA genes. Second, their identification is limited between two cell types and not directly applicable to the data of multiple cell types. Third, they do not control undesired source of variance that potentially introduces noise to the cell-type-specific identification of APA genes. To address these limitations, we developed a combination of a computational change-point algorithm and a statistical model, single-cell Multi-group identification of APA (scMAPA). To avoid the assumptions on the read coverage shape, scMAPA formulates a change-point problem after transforming the 3′ biased scRNA-Seq data to represent the full-length 3′UTR signal. To identify cell-type-specific APA genes while adjusting for undesired source of variation, scMAPA models APA isoforms in consideration of the cell types and the undesired source. In our novel simulation data and human peripheral blood monocellular data, scMAPA outperforms existing methods in terms of sensitivity, robustness, and stability. In mouse brain data consisting of multiple cell types sampled from multiple regions, scMAPA identifies cell-type-specific APA genes, elucidating novel roles of APA for dividing immune cells and differentiated neuron cells and in multiple brain disorders. Altogether, scMAPA elucidates the cell-type-specific function of APA events and sheds novel insights into the functional roles of APA events in complex tissues.
Corresponding Author:	Hyun Jung Park<br>University of Pittsburgh<br>Pittsburgh, PA UNITED STATES
Corresponding Author Secondary Information:	
Corresponding Author's Institution:	University of Pittsburgh
Corresponding Author's Secondary Institution:	
First Author:	Yulong Bai
First Author Secondary Information:	
Order of Authors:	Yulong Bai
	Yidi Qin
	Zhenjiang Fan
	Robert Morrison
	KyongNyon Nam
	Hassane Zarour

Powered by Editorial Manager® and ProduXion Manager® from Aries Systems Corporation

	Radosveta Koldamova
	Quasar Padiath
	Soyeon Kim
	Hyun Jung Park
**Order of Authors Secondary Information:**	
**Response to Reviewers:**	RESPONSE TO REVIEWERS' CRITIQUES<br>Reviewer reports:<br>Reviewer #1: The authors have addressed some of my concerns but not others. The remaining major issue is that the definition of 'confounding factor' is quite confusing. The authors are recommended to provide a more intelligible description.<br><br>Response: We realize that the word "confounding" can mean differently by context, potentially confusing readers. Thus, we removed the word and rewrote those parts with the specific meaning of it, which is "undesired source of variation". To clarify further, we kept our focus on a specific case where brain region information becomes the undesired source of variation in identifying brain cell-type-specific APA genes throughout the manuscript.<br><br>Reviewer #2:<br>The authors present a novel tool scMAPA for the identification and quantification of alternative poly-adenylation sites from scRNA-seq.<br><br>The manuscript has had a substantial re-write and additional analyses performed since the previous submission. It has been improved significantly and previous comments addressed.<br><br>Response: Thank you so much for acknowledging our effort to improve our manuscript.<br><br>Major comments<br><br>In the Findings section there is too much methodology mentioned in it but without the detail so makes reading it harder. In the Findings section a focus should be on the results such as the comparison to Sierra and scAPA and what was identified in the PBMC and Mouse data.<br>Response: Thanks to this reviewer's comment, we moved the descriptions about methodology to Methods section. Previously, there were 2,689 words in Findings section. This will make our manuscript to be more understable. Thank you for your comment.<br><br>The authors need to also ensure the past tense is used consistently throughout. An example<br>is:<br>p.13 para 2 line 14 "we test if they express highly"<br>is better as<br>"we tested if they were expressed highly"<br>Response: Thank you for your suggestion. We made this correction the reviewer suggested. Also, we made the following corrections we found in the same direction.<br>1.P.5 line 9, "this transformation made the APA short and long isoforms readily distinguishable" -> "this transformation makes the APA short and long isoforms readily distinguishable"<br>2.P.6 line 13, "which used the quadratic programming"-> "which uses the quadratic programming"<br>3.P.8 line 6, "the mouse brain data showed a narrow range"-> "the mouse brain data show a narrow range"<br>4.P.9 line 1, "scMAPA consistently outperformed the other methods"-> "scMAPA consistently outperforms the other methods"<br>5.P.9 line 13, "scMAPA identified a two-fold higher percentage of APA genes"-> "scMAPA identifies a two-fold higher percentage of APA genes"<br>6.P.10 line 17, "showed the dynamic APA isoform ratios across the cell types"-> "show the dynamic APA isoform ratios across the cell types"<br>7.P.11 line 4, "While both the analyses supported the previous finding" -> "While both

Powered by Editorial Manager® and ProduXion Manager® from Aries Systems Corporation

the analyses support the previous finding"
8.P.11 line 15, "they further revealed that immune cells are most different from all the other cell types"-> "they further reveal that immune cells are most different from all the other cell types"
9.P.13 line 2, "APA genes associated with a brain region can be mistakenly identified as cell-type-specific APA genes"-> "APA genes associated with a brain region could be mistakenly identified as cell-type-specific APA genes"
10.P.13 line 18, "GTEx does not collect the expression data for midbrain" -> "GTEx did not collect the expression data for midbrain"

On p. 9 para 1 line 4 different numbers of cells were defined: "6, 8, and 13 types for 1k, 5k, and 10k data respectively"
How representative are those numbers? There are 5x more cells in the 5k data than 1k yet only a third more cells types. The 5k vs 10k are more consistent: 2x cells and ~1.5x types.
Response: We determined the number of clusters by running a well-established algorithm. Especially, we used the parameters suggested particular for the 1k, 5k, and 10k data 1. To further validate the number of the clusters, we examined the percentage of variance explained (between-group variance/total variance) against the different number of clusters in elbow plot analysis (R. Fig. 1A, B, C for 1k, 5k, and 10k data respectively). From the elbow plots, we can see that the number of clusters was set in an acceptable range of the explained variance (between the steepest increase and the flattening point), suggesting that Seurat's method delineated an appropriate number of clusters in the 1k, 5k, and 10k data. Especially, although 5x more cells in the 5k data did not proportionally increase the number of clusters from the 1k data, the defined clusters explain a very similar percentage of the variance (~16.25%), supporting the number of clusters in the 1k and 5k data again.
Another support comes when checking the dimension-reduced space (UMAP) of the data (R. Fig. 1D, E, F for 1k, 5k, and 10k data respectively), since distinct cell types are expected to be well separated on the UMAP. Since it is the case for the 1k, 5k, 10k data, we believe that the numbers of the defined clusters were set appropriately.

p. 10 para 1 line 3. scMAPA found 40.7% genes as being APA compared to the other tools which found between 11.6-18.9%. Do the authors know whether that is a biological valid percentage?
Response: To identify a valid percentage in our experiment investigating how much APA genes are identified in different numbers of cells sampled, we ran scMAPA on the 1k and 10k data to find that 51.5% of the APA genes are common. Since the data were sampled from a same healthy donor, the number of APA genes common to the 1k and 10k data indicates the upper bound of the identification attributing mainly to the different number of cells in the data, and thus representing a biologically valid percentage for our experiment. Since 40.7% of genes commonly being APA is lower than this percentage, we believe that the results are valid, suggesting that scMAPA is most robust to the sample size changes.
Separately, to identify a biologically valid percentage in general, we searched literature reporting the number of APA events. Using a sequencing technique designed to comprehensively map polyadenylation sites, a recent study found that 78.5% of mRNA genes were found to undergo APA events on the RNA samples across a more diverse set of samples ((i) male and female whole bodies; (ii) embryos at 11, 15 and 17 days (d); (iii) brain and testis tissues at different postnatal stages; and (iv) over 11 cell lines2).

p. 10 para 2 line 4: "enrichments to 32 IPA terms that are characterized with keywords "blood" and "hematology", suggesting that the APA genes identified by scMAPA can play important roles in PBMC biology".
The PBMC dataset is a blood cell dataset which one would be always enriched with the terms "blood" and "hematology". Is that not so? How is the IPA returning enrichment for these terms a measure of scMAPA accuracy?

The reviewer is right that any functional component in the blood cells is expected to be enriched with keywords "blood" and "hematology". Based on this rationale, we wanted to see if the identified APA genes implicate any functions in the blood cells by inspecting their enrichment terms. To conduct this analysis stringently and reduce the chance of random enrichment for the keywords, we "set the 18,804 genes expressed

	in the data as the background" in the analysis, so, for the APA genes to be significantly enriched with the "blood" and "hematology" terms, the enrichment degree should exceed what could be expected generally from the expressed genes in the PBMC data.<br>Also, the reviewer is right that these enrichments couldn't be a measure of scMAPA accuracy. That is why we did not claim scMAPA accuracy with this finding, but to suggest "that the APA genes identified by scMAPA can play important roles in PBMC biology".<br><br>p. 11 para 1 line 9: "Since bone marrow is developmentally related to peripheral blood, GATA2 may undergo the APA event in the PBMC under similar molecular mechanisms."<br>This statement needs to be supported with further evidence or the authors should say this is speculation.<br><br>Response: We changed the text in the manuscript as follows. "Since hematopoietic stem and progenitor cells (HSPC in Fig. 1C, D) are originated from bone marrow3, we speculate that the molecular mechanisms rendering the APA event on GATA2 in the bone marrow mononuclear cells cause GATA2 to show different APA patterns than other cells in the PBMC."<br><br>Minor comments<br><br>Abstract: para 2, line 3: "To release the assumptions" should be "To avoid the assumptions"<br>Response: This has been changed according to your suggestion.<br><br>p-values reported in scientific notation should be in the form 2.2 x 10^-16 not 2.2e^-16 as reported on p. 7 para 2 line 15. Also 10^-2 (p. 10 para 2 line 5) is better as 0.01<br>Response: We changed "p<2.2e^-16" into "p-value<2.2×10-16". Also, we changed "B-H p<10^-2" to "0.01". Further, para 3 had two occurrences of "B-H P-val < 0.05". We changed them into "B-H p-val < 0.05" to make the same style. Several other places had similar issues. We changed them into the same style.<br><br>p. 10 para 2 line 4: spell out acronyms the first time they're used: B-H as Benjamini-Hochberg<br>Response: This has been changed according to your suggestion.<br><br>p. 11 para 1 line 10: "biologically reasonable APA genes" should be "biologically relevant APA genes"<br>Response: This has been changed according to your suggestion.<br><br>p. 14 para 2 line 9: is "10^3.5" what is meant here? Re-write in proper scientific notation as mentioned above.<br>Response: We changed it into "p-value < 2.2×10-4".<br><br>p. 22 para 1 line 6: "at least 20 raw counts" of what?<br>Response: It is "at least 20 raw counts of reads". With this change, we updated that part as follows. "In addition to gene-wise filtering, we also apply cell-wise filtering for each passed gene to keep only cell types with at least 20 raw counts of reads in the model. For each gene, cell types with extremely low coverage (< 20) will not be used to estimate the APA status."<br><br>p. 24 para 2 line 4: "than ln(2), corresponding to a 2-fold change in odds ratio" is incorrect as ln() is the natural log so ln(2) equals 0.693<br>Response: We used logistic regression where ln(2) corresponds to a 2-fold change in odds ratio. However, we understand that this might confuse readers. So, we changed the text as follows. "we further selected genes whose APA degrees change greater than 2-fold. If the APA degree increases greater than 2-fold, the respective gene is considered as 3′-UTR lengthening. And, if the APA degree decreases less than 2-fold, the respective gene is considered as 3′-UTR shortening. However, users can define a different cutoff value of fold change to call 3′-UTR lengthening or shortening."

Powered by Editorial Manager® and ProduXion Manager® from Aries Systems Corporation

	p. 24 para 3 line 1: "scMAPA can be easily extended" is better as "scMAPA has been extended".<br>Response: Although we decided not to do the extension in this manuscript for fair comparisons with other methods and for effective investigation of multiple cell types (see 3rd para in Discussion), we agree with the reviewer that this extension will be helpful for further analyses. We will work on this extension as future work. We made this point more specific in the 3rd para in Discussion.
Additional Information:	
Question	Response
Are you submitting this manuscript to a special series or article collection?	No
**Experimental design and statistics**<br><br>Full details of the experimental design and statistical methods used should be given in the Methods section, as detailed in our Minimum Standards Reporting Checklist. Information essential to interpreting the data presented should be made available in the figure legends.<br><br>Have you included all the information requested in your manuscript?	Yes
**Resources**<br><br>A description of all resources used, including antibodies, cell lines, animals and software tools, with enough information to allow them to be uniquely identified, should be included in the Methods section. Authors are strongly encouraged to cite Research Resource Identifiers (RRIDs) for antibodies, model organisms and tools, where possible.<br><br>Have you included the information requested as detailed in our Minimum Standards Reporting Checklist?	Yes
**Availability of data and materials**<br><br>All datasets and code on which the conclusions of the paper rely must be either included in your submission or deposited in publicly available repositories	Yes

Powered by Editorial Manager® and ProduXion Manager® from Aries Systems Corporation

(where available and ethically appropriate), referencing such data using a unique identifier in the references and in the "Availability of Data and Materials" section of your manuscript.<br><br>Have you have met the above requirement as detailed in our Minimum Standards Reporting Checklist?	

Manuscript Click here to access/download;Manuscript;ver25_ummarked.pdf

scMAPA: Identification of Cell-type-specific Alternative Polyadenylation in Complex Tissues

Yulong Bai[1], Yidi Qin[1], Zhenjiang Fan[2], Robert M. Morrison[5,6,7], KyongNyon Nam[3], Hassane Mohamed Zarour[5,6], Radosveta Koldamova[3], Quasar Saleem Padiath[1,4], Soyeon Kim[7,8†], Hyun Jung Park[1†]

[1] Department of Human Genetics, Graduate School of Public Health, University of Pittsburgh, Pittsburgh, USA

[2] Department of Computer Science, School of Computing and Information, University of Pittsburgh, Pittsburgh, USA

[3]Department of Environmental and Occupational Health, Graduate school of Public Health, University of Pittsburgh, Pittsburgh, USA

[4]Department of Neurobiology, School of Medicine, University of Pittsburgh, Pittsburgh, USA

[5]Department of Medicine and Division of Hematology/Oncology, University of Pittsburgh, School of Medicine, Pittsburgh, USA

[6]Department of Immunology, University of Pittsburgh, School of Medicine, Pittsburgh, USA

[7]Department of Computational and Systems Biology, University of Pittsburgh Medical Center, Pittsburgh, USA

[8]Department of Pediatrics, University of Pittsburgh Medical Center, Pittsburgh, USA

[9]Division of Pulmonary Medicine, Children's Hospital of Pittsburgh of UPMC, Pittsburgh, Pennsylvania, USA

†senior author; Correspondence: hyp15@pitt.edu (H.J.P.)

ABSTRACT

Alternative polyadenylation (APA) causes shortening or lengthening of the 3′-untranslated region (3′-UTR) of genes (APA genes) in diverse cellular processes such as cell proliferation and differentiation (cell-type-specific APA). To identify cell-type-specific APA genes in scRNA-Seq data, current bioinformatic methods have several limitations. First, they assume certain read coverage shapes in the scRNA-Seq data, which can be violated in multiple APA genes. Second, their identification is limited between two cell types and not directly applicable to the data of multiple cell types. Third, they do not control undesired source of variance that potentially introduces noise to the cell-type-specific identification of APA genes. To address these limitations, we developed a combination of a computational change-point algorithm and a statistical model, single-cell Multi-group identification of APA (scMAPA). To avoid the assumptions on the read coverage shape, scMAPA formulates a change-point problem after transforming the 3′ biased scRNA-Seq data to represent the full-length 3′UTR signal. To identify cell-type-specific APA genes while adjusting for undesired source of variation, scMAPA models APA isoforms in consideration of the cell types and the undesired source. In our novel simulation data and human peripheral blood monocellular data, scMAPA outperforms existing methods in terms of sensitivity, robustness, and stability. In mouse brain data consisting of multiple cell types sampled from multiple regions, scMAPA identifies cell-type-specific APA genes, elucidating novel roles of APA for dividing immune cells and differentiated neuron cells and in multiple brain disorders. Altogether, scMAPA elucidates the cell-type-specific function of APA events and sheds novel insights into the functional roles of APA events in complex tissues.

Keywords: post-transcriptional regulation, alternative polyadenylation, single-cell RNA, cell-type-specific regulation, confounding factors

BACKGROUND

Many mammalian messenger RNAs contain multiple polyadenylation (pA) sites, e.g., proximal and distal, in their 3′-untranslated region (3′-UTR) [1], [2]. Using multiple pA sites in each gene, alternative polyadenylation (APA) post-transcriptionally produces multiple APA isoforms with various 3′-UTR lengths. These APA events are involved in diverse cellular processes such as cell proliferation and differentiation in particular cell types. For example, cancer cells of diverse types are reported to undergo widespread 3′UTR shortening events [3], whereas senescent cells tend to show widespread 3′UTR lengthening events [4]. To identify such APA genes for each cell type (cell-type-specific APA genes) in complex tissues, developing a computational method that accurately analyzes single-cell RNA sequencing (scRNA-Seq) data is essential since the data presents the cell-type-specific transcriptome.

To identify cell-type-specific APA genes in scRNA-Seq data, several bioinformatic methods have been developed, such as scDAPA[5], Sierra [6], and scAPA [7]. Although they have various strengths, they also have several limitations to be used for complex tissue data. First, they only consider certain read coverage shapes in the input scRNA-Seq data to estimate APA events. This is because several scRNA-Seq techniques generate the 3′ enriched reads and the accumulation of the reads that originate from the same APA isoform forms a peak. To identify the signal part of the peak from noise, the existing methods assume certain signal shapes

in their peak calling. For example, scAPA utilizes findPeaks module in Homer package [8] with the preset peak size and height. However, these assumptions can be violated in multiple genes across multiple cell types. For example, one would be interested in quantifying APA isoforms of FLT3 and GATA2 in the scRNA-Seq data on Peripheral Blood Monocellular Cells (PBMC) of a healthy donor (10k in https://www.10xgenomics.com/) since their abnormality may lead to blood disorders [9], [10]. However, their 3′ tags form peaks with different sizes and heights across various cell types (**Fig. 1A, C**) that the existing methods would not be able to identify peaks from some of the cell types. Second, the existing methods cannot identify cell-type-specific APA genes when the scRNA-Seq data contains more than two cell types, which is typical for complex tissues. scDAPA and Sierra are only able to compare cell types in a pairwise fashion, which limits their ability for global comparison when more than two cell types exist. While scAPA is the only method to identify APA genes for multiple cell types, it identifies genes in which the APA isoform ratio (the ratio of long and short 3′-UTR isoforms) varies across the cell types and does not further identify which specific cell types drive this variation. Third, the existing methods do not adjust for other factors that affect the scRNA-Seq data across cell types. For example, when the scRNA-Seq data are sampled from various brain regions, some cell types reside in multiple brain regions [11]. Then, molecular dynamics specific to the brain regions would affect different portion of the residing cell types, introducing noise to the cell-type-specific identification of APA genes. Thus, to identify cell-type-specific APA genes, one may need to adjust for the brain region information. Fourth, there is no simulation platform to compare statistical power and specificity of the methods identifying APA genes in scRNA-Seq data. Although such a platform is necessary to evaluate the methods with the ground truth, it has

been challenging to simulate APA and non-APA genes since it is not clear how the read coverage shapes differ between APA and non-APA genes.

To address these limitations, we developed a combination of a computational optimization algorithm and a statistical model, single-cell Multi-group identification of APA (scMAPA). To address the first limitation and quantify APA isoforms without assumptions on the read coverage shape, scMAPA first transforms the input scRNA-Seq data and then formulates a change-point detection problem on the transformed data. First, scMAPA transforms the 3′-enriched signal of scRNA-Seq data to represent the full-length 3′UTR signal. For FLT3 and GATA2 in the PBMC of a healthy donor, this transformation makes the APA short and long isoforms readily distinguishable across all cell types regardless of the differences in read coverage shape (**Fig. 1B, D**). Then, on the transformed coverage shapes, scMAPA quantifies APA isoforms by detecting a change-point. To address the second and the third limitations to identify cell-type-specific APA genes while controlling undesired source of variation, scMAPA considers cell type information and the undesired source by developing a statistical model with them as covariates . To address the fourth limitation and simulate APA genes, we identified a common feature of APA genes in real data, a high variance in the APA isoform ratios across cell types and simulate the APA isoform specific count matrix based on the common feature. Since this simulation platform does not generate data at the level of read coverage shape, it can generate the ground truth APA genes without having to resolve the difference between APA and non-APA genes in the read coverage shape. By systematically addressing these limitations, scMAPA accurately and robustly identifies cell-type-specific APA genes and facilitates a systematic understanding of APA regulation in complex tissues in this manuscript.

FINDINGS

Single-cell multi-group identification of alternative polyadenylation (scMAPA)

To identify cell-type-specific APA genes accurately and robustly, scMAPA combines a computational algorithm and a statistical model in three steps. First, scMAPA transforms each read in the scRNA-Seq data by padding it from the annotated 3'UTR start site to where the read ends (step 1 in **Fig. 1E**). While the scRNA-Seq reads are usually 3' biased due to the 3′ selection and enrichment techniques in the library construction step, the transformed reads will represent the read coverage shape across the 3'UTRs. Second, scMAPA identifies a pA site that minimizes the difference between the expected coverage shape of the inferred APA isoforms and the accumulated observed coverage (change-point, step 2 in **Fig. 1E**). Since the difference can be calculated by a quadratic function, scMAPA detects the change-point by quadratic programming[12]. To solve this problem for multiple cell types in scRNA-Seq data, scMAPA extends multiple modules of DaPars2 [13], which uses the quadratic programming approach to identify APA genes in bulk RNA-Seq data. Third, to simultaneously identify APA genes across cell types and for each cell type based on the APA isoforms quantified, scMAPA develops a multinomial regression model that explicitly models each APA isoform (step 3 in **Fig. 1E**) with covariates representing the cell type and other source of variation (step 4 in **Fig. 1E**). On the model, scMAPA uses the log-likelihood test and the Wald test to identify across-cell-type APA genes and cell-type-specific APA genes, respectively. Altogether, scMAPA is the first method to simultaneously identify across-cell-type and cell-type-specific APA genes in scRNA-Seq data of multiple cell types.

scMAPA outperforms the other method in sensitivity for the multi-group setting

To assess the performance of scMAPA using the ground truth, we developed a novel simulation platform where APA isoform-specific expressions are simulated in multiple steps. First, to learn parameters from real data, we determined APA genes across five cell types of a mouse brain scRNA-Seq data [11] (neurons, astrocytes, immune cells, oligodendrocytes, and vascular, step 0 in **Fig. 2A**) as those identified by both scAPA and scMAPA. We used only scAPA and scMAPA since they are the only methods designed for more than two cell types. Then, we quantified a common feature of the APA genes by calculating the proportion of the long and short isoforms in each cell type and the standard deviation of the proportions across the five cell types ($SD_{isoprop}$, see Methods). To validate the effectiveness of this measure for APA simulation, we calculated $SD_{isoprop}$ values for non-APA genes that scAPA and scMAPA agreed on in the data. We found that $SD_{isoprop}$ values significantly distinguish APA genes from non-APA genes (0.127 vs. 0.009 of $SD_{isoprop}$ on average, p-value<10^{-16}, **S. Fig. 2A**), suggesting that it is reasonable to simulate APA genes to have high $SD_{isoprop}$ values in the data of multiple (≥2) cell types (multi-group setting).

To simulate APA long and short isoform expressions, we simulated gene expression values for 5 simulated cell clusters (step 3 in **Fig. 2A**) and divided the values into APA long and short isoforms based on the $SD_{isoprop}$ values. Since the $SD_{isoprop}$ values are the standard deviation of APA long and short isoform ratios, the simulation based on the high $SD_{isoprop}$ values estimated from the APA genes spreads the APA long and short isoform expressions across the 5 cell clusters. In the same sense, the simulation based on the low $SD_{isoprop}$ values produces a less variable isoform expressions across the clusters. On the simulated APA isoform

expressions for APA and non-APA genes, we ran scMAPA and scAPA to assess their sensitivity and specificity. In the first scenario simulating 500 APA and 4,500 non-APA genes, we varied $SD_{isoprop}$ values for APA genes in the range observed in the mouse brain data (0.06 to 0.18, **S. Fig. 2A**). Across all simulated $SD_{isoprop}$ values, scMAPA consistently outperforms scAPA with higher sensitivity (**Fig. 2B**) while having a similar specificity (**Fig. 2C**). In assessing specificity, we did not vary $SD_{isoprop}$ values for non-APA genes, since the mouse brain data show a narrow range of $SD_{isoprop}$ values for non-APA genes (**S. Fig. 2A**). In the second scenario, we varied the number of APA and non-APA genes and the cell group size while fixing the $SD_{isoprop}$ values for APA and non-APA genes (to 0.127 and 0.009, respectively). With various numbers of true APA genes (250, 500, and 1,000), scMAPA consistently outperforms scAPA in terms of sensitivity (**Fig. 2D and S. Fig. 2B, D**) with a slight loss of specificity (**Fig. 2E and S. Fig. 2C, E, F**). To sum, scMAPA outperforms scAPA in various simulation scenarios in terms of sensitivity with a similar level of specificity.

scMAPA outperforms existing methods in identifying APA isoforms with high robustness

To assess the performance of scMAPA using real data, we used three PBMC data sets of various numbers of cells (1k, 5k, and 10k data representing the number of cells) from 10x Genomics website (see Methods, **S. Table 1**). To assess the accuracy of scMAPA in identifying annotated pA sites, we identified pA sites in the 10k and 5k data using scMAPA, scAPA, and Sierra. scDAPA was not included in this comparison, because it does not return results that are compatible for the comparison, such as pA peaks, sites, or intervals. Among the identified pA sites, we calculated the proportion of them that are close to the annotated pA sites in PolyASite

2.0[14] (see Methods). scMAPA consistently outperforms the other methods by identifying the highest proportion of the annotated pA sites across all degrees of proximity (**Fig. 3A**, **S. Fig. 3A, B**). This result suggests the outperformance of scMAPA in identifying possible bona fide APA events originated from the annotated pA sites.

We further evaluated the robustness of the methods in two ways. First, we ran scMAPA, scAPA, scDAPA, and Sierra to identify APA genes in the 1k, 5k, and 10k PBMC data. Since the 1k, 5k, and 10k data sets comprise similar sets of cell types from healthy adults (1k and 10k from the same donor and 5k from another healthy donor, **S. Table 1**), the APA genes are expected to overlap across the data sets. Thus, a high percentage of APA genes identified commonly across the data sets would indicate the robustness of the methods to the number of cells in the data. Although Sierra and scDAPA cannot identify APA genes directly from multiple (>2) cell types, we artificially identified the APA genes for multiple cell types by combining all pairwise identifications after FDR control (see Methods). Compared to the competing methods, scMAPA identifies a two-fold higher percentage of APA genes commonly across the 3 types of the data sets (40.7% vs. 18.9%, 11.6%, and 18.6% respectively, **Fig. 3B**), showing that scMAPA identifies APA genes robustly to the number of cells in the data. Second, from the 10k data comprising the total of 13 cell types, we randomly sampled various numbers of cell types (5, 7, 9, and 11) and ran scMAPA and scAPA separately in each sample. For direct comparison, we compared scMAPA only with scAPA, the only other method that can directly handle the multi-group setting. In the APA genes identified in each sample (sample APA genes), we calculated the overlap with those identified using all the 13 cell types (total APA genes). Then, we calculated APA agreement ratio, defined as the number of the overlap between the sample and total APA genes normalized by the number of total APA genes. In all the numbers of cell types

sampled, scMAPA outperforms scAPA with higher APA agreement ratios (**Fig. 3C**). Since the APA agreement ratio indicates the number of the total APA genes that are found in the sample APA genes, the result shows that scMAPA identifies APA genes robustly to the number of cell types in the data.

Further, to investigate if the APA genes identified by scMAPA are biologically relevant, we performed Ingenuity Pathway Analysis (IPA) on 3,574 APA genes that scMAPA identified in the 10k PBMC data. Especially, to accurately investigate the APA genes' roles in PBMC biology, we set the 18,804 genes expressed in the data as the background (see Methods). This IPA analysis shows significant (Benjamini-Houchberg (B-H) p-value < 0.01) enrichments to 32 IPA terms that are characterized with keywords "blood" and "hematology" (**Fig. 3D**), suggesting that the APA genes identified by scMAPA can play important roles in PBMC biology.

To examine the unique contribution of scMAPA in characterizing the function of APA genes for PBMC biology, we manually inspected 1,432 APA genes that are identified only by scMAPA, not by other methods (scMAPA-unique APA genes, **S. Table 2**). In the scMAPA-unique APA genes, we found clear changes in the APA isoform ratios across the cell types and great potential to function for PBMC biology. For example, FLT3 and GATA2 are included in the scMAPA-unique APA genes and show the dynamic APA isoform ratios across the cell types especially after the data transformation step of scMAPA (**Fig. 1B, D**). Interestingly, GATA2 is an APA gene in the scRNA-Seq data of bone marrow mononuclear cells from acute myeloid leukemia patients [15]. Since hematopoietic stem and progenitor cells (HSPC in **Fig. 1D**) are originated from bone marrow[16], we speculate that the molecular mechanisms rendering the APA event on GATA2 in the bone marrow mononuclear cells cause GATA2 to show different

APA patterns than other cells in the PBMC. Together, scMAPA enables accurate and robust identification of biologically relevant APA genes in the PBMC scRNA-Seq data.

scMAPA estimates APA effect size and identifies APA genes across multiple cell types

Compared to other methods, scMAPA is the only method that can estimate the effect size and the significance of APA events for each cell type in the multi-group setting (see Methods). To demonstrate how the APA effect size enables us to understand the post-transcriptional regulation in each cell type, we analyzed the mouse brain scRNA-Seq data comprising five major cell types: neurons, astrocytes, immune cells, oligodendrocytes, and vascular [11] (**Fig. 4A,** see Methods). First, to identify the distances among the cell types in terms of the APA effect size, scMAPA estimated the effect size of 3,223 genes significantly (B-H p-val < 0.05) identified as APA genes across the five cell types (**Fig. 4B**). Based on these effect sizes, we performed the PCA analysis (**Fig. 4C**) and calculated Euclidean distance (**S. Fig. 4A**) between the cell types. While both the analyses support the previous finding that immune and neuron cells are most different in terms of the APA effect size [7], they further reveal that immune cells are most different from all the other cell types. Second, to identify the overall relationships between the APA regulation and the gene expression regulation, we correlated the APA effect sizes of all the identified genes with their expression level. The result shows that the APA effect sizes are not correlated with their expression level in all the cell types (e.g., Spearman's $\rho < 0.05$ for all cell types, **S. Fig. 4D-H**), demonstrating that APA events are regulated independently of gene expression in the mouse brain.

Further, cell-type-specific APA genes (3′-UTR shortening and lengthening genes) identified by scMAPA provide a systematic understanding of cellular status. Previous studies showed that APA is involved in regulating cell division status. For example, various types of dividing cells are associated with widespread 3′-UTR shortening [17], [18]. Likewise, differentiated and senescent cells are associated with widespread 3′-UTR lengthening [19], [20]. To systematically extend these findings that were made in cell line data [17], [19], [21] or heterogeneous tissue data [18], we ran scMAPA in the mouse data further to identify 438 significant (B-H p-val < 0.05) cell-type-specific APA genes in neurons, 891 in immune, 374 in astrocyte, 422 in vascular, and 430 in oligos with some overlaps across the cell types (**S. Fig. 4B**). A further division into 3′-UTR shortening and lengthening genes in each cell type (**Fig. 4D)** showed that 3′-UTR shortening and lengthening are significantly enriched in immune cells and neuron cells, respectively. As immune cells actively divide to dynamically regulate the immune system, the enriched 3′-UTR shortening may contribute to the active division. In the same sense, we could find a biological explanation for why 3′-UTR lengthening are enriched in neurons. While neurons do not divide once they are formed in the brain, our result suggests that the 3′-UTR lengthening can play a significant role in keeping neuron cells from further dividing. Together, by identifying cell-type-specific APA genes, scMAPA systematically links the cellular APA profile to dividing immune cells and differentiated neuron cells.

scMAPA adjusts for undesired source of variance to uncover APA functions which would be invisible without the adjustment

To show how scMAPA controls undesired source of variance in the data and why it is important, we analyzed the mouse brain data consisting of 5 cell types collected from 2 brain regions

(cortex and midbrain). Since some cell types were collected from multiple brain regions (**Fig. 4A, 5A**), APA genes associated with a brain region could be mistakenly identified as cell-type-specific APA genes, which would further confuse the study of cell-type-specific functions of APA genes. To see if scMAPA can remove such false positive APA genes, we set scMAPA to adjust for the brain region information (cortex and midbrain dorsal) (brain-region-adjusted scMAPA). Then, we compared the result from another scMAPA run that does not adjust for that information(brain-region-unadjusted model), separately. As the brain-region-adjusted scMAPA and the brain-region-unadjusted model identified 2,715 and 2,793 APA genes respectively (**S. Table 6**), 113 genes are not identified in the brain-region-adjusted scMAPA. Thus, these APA genes are expected to be related to the brain region it was sampled from (cortex and midbrain) (**Fig. 5B**). To test if the 113 genes function specifically for the brain region, we tested if they express highly specifically in the brain region. To conduct this test comprehensively, we identified their human homolog genes in the Mouse Genomic Informatics (MGI) homology database and compared expression of the human homologs between cortex and other brain regions in the Genotype-Tissue Expression (GTEx) [22] (see Methods). The result shows that these APA genes are significantly up-regulated in brain cortex compared to other brain regions (p-value=5.8×10^{-7}, **Fig. 5C**), suggesting that their functions are specific to brain cortex. Since GTEx did not collect the expression data for midbrain, we did not conduct this analysis for midbrain. This result suggests that scMAPA can successfully adjust for undesired source of variation and identify APA genes likely caused by differences between cell types.

To demonstrate why adjusting for undesired source of variation is critical for accurate downstream analysis, we further conducted IPA analysis on the 2,715 and 2,793 APA genes identified by the brain-region-adjusted scMAPA and the brain-region-unadjusted model

respectively (brain-region-adjusted and -unadjusted APA genes, respectively). Comparing the IPA enrichment between brain-region-adjusted and brain-region-unadjusted APA genes, we found considerable differences in important terms for brain study: among the 24 terms to which the brain-region-adjusted APA genes are uniquely and significantly (B-H p-value < 0.01) enriched, 7 terms are directly related to brain diseases (**Fig. 5D**). For example, two terms with the keyword "mental retardation" are significantly enriched (B-H p-value < 2.2×10^{-4}) only for the brain-region-adjusted APA genes. On the other hand, among the 30 terms to which the brain-region-unadjusted APA genes are uniquely and significantly enriched, no term refers to a brain disease (**S. Fig. 5A**). With potential roles of APA events in brain diseases[23], [24], this result suggests that adjusting for the variation from brain region uncovers the APA genes that can play critical roles in the brain disease, which would be invisible without the adjustment.

Supplemental material

APA regulation on expression

Previous studies have suggested that APA genes are more likely differentially expressed[1], [2], since either 3′-UTR shortening removes microRNA (miRNA) binding sites on the 3′-UTR and evades miRNA-mediated repression or 3′-UTR lengthening adds miRNA binding sites and enhance miRNA-mediated repression. Our analysis reaffirms the previous observations in the scRNA-Seq data.

scMAPA consensus with other methods

In the PBMC data, scMAPA results still recover most of the results from the other methods. To assess the overlap, we identified significant APA genes across all the cell types in scMAPA and

scAPA. Since scDAPA and Sierra identify APA genes only between cell-type pairs, we combined the pairwise significant APA genes in each method separately. After controlling FDR on the combined APA genes, we called APA genes if they are significant in any of the pairwise identifications. While scMAPA identifies an intermediate number of APA genes between scDAPA and Sierra/scAPA (10k in **S. Fig. 3C** and 5k in **S. Fig. 3D**), more than half of the scMAPA's findings are found in other methods (59.9% for 10k and 51.9% for 5k). While scMAPA solves an optimization problem based on the padding of 3′ biased reads (step 1 in **Fig. 1C**), it successfully recovers most results from other methods, validating the use of scMAPA for comprehensive identification.

Cell-type-specific APA genes in 10k PBMC data

The global size differences in PBMC cells are different from in the mouse brain data in several aspects. First, 3'UTR lengthening occurs more than 3'UTR shortening in all the cell types (**S. Fig. 4E**). Second, however, the number of 3'UTR shortening genes is significantly correlated with that of lengthening genes across the cell types (P-value=5×10^{-5}, **S. Fig. 4F**). Since both trends are not shown in the mouse brain data, scMAPA elucidates the unique APA profiles of the PBMC data.

Specificity of high expression in 113 brain-region-related APA genes for the brain cortex region

In demonstrating the high expression of the 113 brain-region-related APA genes in the brain cortex region, we further investigated if the APA genes are not down-regulated in neither brain vs. non-brain samples (**S. Fig. 5B)** nor cortex vs. non-cortex brain samples (**S. Fig. 5C)**. Also, this brain-region-specific expression pattern was not found for 2,715 APA genes identified by the brain-region-adjusted scMAPA (**S. Fig. 5D, E, F, G**). Together with our analysis on up-

regulation (**Fig. 5**), the results suggest that the 133 APA genes function specific to the brain region.

Discussion

To identify APA genes in scRNA-seq data for complex tissue data, we developed scMAPA that addresses several limitations in existing methods using a combination of a computational optimization algorithm and a statistical model. First, while existing methods detect APA signals with assumptions on the shape of the input data, scMAPA does not rely on such assumptions by formulating this task in quadratic programming. By solving this quadratic programming for genes with different read coverage shapes across cell types, scMAPA outperforms existing methods in accurately and robustly identifying APA genes in various simulated (**Fig. 2**) and PBMC data (**Fig. 3**). Second, scMAPA identifies APA genes specific to each cell type in a statistically rigorous model. These cell-type-specific APA genes elucidates their connections to the cell division status of immune and neuron cells in the mouse brain data (**Fig. 4**). Third, scMAPA can control confounding factors. In the mouse brain data of five cell types collected from two brain regions, scMAPA can distinguish the 113 APA genes that are likely related to the brain regions. By removing the false positive APA genes from further analyses, scMAPA could clarify the functions of APA genes on brain diseases such as 'mental retardation' (**Fig. 5**). Lastly, we developed a novel simulation platform in which to assess statistical power of APA identification methods based on a common feature of APA genes, the high variation of APA long and short isoforms ($SD_{isoprop}$) across cell clusters.

When identifying the annotated pA sites, scMAPA makes point estimations of the pA sites. While other methods mainly produce interval estimates, point estimations are more directly relevant to further analyses than interval estimations, e.g., conducting omics data analyses and designing validation experiments. However, when point estimation methods are naively compared to interval estimation methods in terms of the distance to the annotated pA sites, point estimations produce generally disadvantageous results, because point estimation returns a single point while interval estimation returns two points (start and end of the interval) to measure the distance. For example, the interval estimations produce better results than the point estimations within both Sierra and scAPA (**S. Fig. 3A, B**). Even with this disadvantage of point estimation for comparison purposes, the point estimation of scMAPA outperforms the interval estimation results of Sierra and scAPA in identifying the annotated pA sites, showing a clear advantage of scMAPA (**Fig. 3A, S. Fig. 3A, B**).

A limitation of this paper is that, although scMAPA can consider more than two pA sites (see Methods), our analysis focused on the use of two pA sites (most distal and most proximal) for the following reasons. First, some of the methods that compare with scMAPA consider only two pA sites, e.g. scAPA. For fair comparisons, we limited scMAPA to consider two pA sites. Second, we focused on this binary APA trend to make it easier to investigate across multiple cell types. In the future, we plan to consider more than two pA sites in complex tissues after characterizing the binary trend across multiple cell types. For example, after solving the quadratic programming with >2 pA sites and developing a multinomial logistic regression model with the identified pA sites in the mouse brain data, we can estimate the APA effect size for each use of the multiple pA sites.

scMAPA can be extended in the following directions in the future. First, the transformation step of scMAPA allows us to use other methods originally developed for bulk RNA-Seq data (e.g. APATrap [25], TAPAS [26]) to analyze scRNA-Seq data. Since the methods can identify APA genes in the full-length 3′ UTR signal of transcripts, scMAPA can employ such methods on the transformed scRNA-Seq data that represents the full-length 3′ UTR signal of transcripts. This extension can make those APA identification methods as reasonable alternatives since those methods are well established and studied in terms of sensitivity and specificity. Second, while existing methods developed for scRNA-Seq data are mostly designed for 3′biased scRNA-Seq data (e.g. 10x), scMAPA can be used for the scRNA-Seq data that are not 3′biased (e.g. Smart-seq2 [27]) simply by skipping the data transformation step, since the scRNA-Seq data already present the full-length 3′-UTRs.

Altogether, we developed scMAPA to identify APA genes in scRNA-Seq data of multiple cell types. With high sensitivity and robustness in addition to adjusting for undesired source of variations, scMAPA elucidates the cell-type-specific function of APA events, which is essential to shed novel insights into the functional roles of APA events in complex tissues.

METHODS

Processing data sets

PBMC data. Aligned BAM files were downloaded from the 10X genomics repository (https://support.10xgenomics.com/single-cell-gene-expression/datasets). According to the data description of 10X, 1K, and 10K data were generated from the same materials. 5K data was

generated from different cells. PCR duplicates were removed using UMI-tools 1.0.0 with "--method=unique --extract-umi-method=tag --umi-tag=UB --cell-tag=CB". Cell clustering was performed using R package Seurat 3.1.4[28]. We filtered to keep cells with more than 1000 UMI counts and 500 genes expressed. Cells with more than 15% UMI counts from mitochondrial genes were filtered out. Then, raw data were normalized by regressing against UMI count, mitochondrial mapping percentage, and ribosome genes mapping percentage using SCTransform function. We ran PCA analysis and took the top 20 principal components as input to FindNeighbors function. Finally, FindClusters function was run with resolution set to 0.2 to identify cell communities. Cell types were annotated by matching the expression pattern of well-known marker genes for PBMC [29].

Mouse brain data. Aligned BAM file and clustering results of cortex and midbrain dorsal from two donors were downloaded from [11]. PCR duplicates were removed using UMI-tools[30] same parameters used for PBMC data. To keep consistent with the analysis performed by scAPA, we included only neurons, immune cells, astrocytes, oligos, and vascular cells in our analysis. Differential expression analysis was performed by FindAllMarkers function of Seurat package with min.pct set to 0.25 and all other parameters as default.

Investigating sample-specific up-regulated genes in GTEx

First, the mouse-human homology data was downloaded from the Vertebrate homology database in the Mouse Genome Informatics (MGI) (http://www.informatics.jax.org/homology.shtml) and used to find homologs in human. Then, we ranked GTEx samples based on the overlap between the upregulated genes and the homolog genes using a database that curates the up- and down-regulated genes for each GTEx sample, Enrichr [31]. Enrichr evaluates the overlap by combining

p-value and odds ratio (Combined Score in Enrichr). We could not conduct this analysis for the midbrain dorsal region, since the GTEx did not collect data from the region.

scMAPA algorithm

Step 0. *Split aligned reads by cell clusters.*

scMAPA takes aligned BAM files and user-provided clustering information (e.g. cell type) as a match table to split the whole BAM file into each cluster using pysam. Clustering information should include all the categorical variables that the user would like to consider in the modeling, but not only cell type. For example, when detecting APA genes in the mouse brain data, we used both brain region and cell type as covariate variables. After splitting, UMI-tools is used to remove the PCR duplicates by grouping reads that sharing the same UMI. Further, scMAPA can identify false APA identifications due to internal priming of A-rich internal regions if more than 7 consecutive adenines with up to 1 mismatch exists in 10 nt downstream of the predicted proximal PA site[14]. In the PBMC 10K data, we identified that 90 out of 3574 APA events are due to suspected internal priming according to this standard.

Step 1. *Pad reads along the 3'UTR after preprocessing.*

We transform aligned scRNA-Seq data that utilize 3′ selection and/or enrichment techniques in library construction (e.g. Drop-Seq, CEL-Seq, and 10x Genomics). A 3′ biased read assigned to the 3′UTR of a gene represents the most 3′ end part of the transcript. With this reasoning, we extend the 3′ biased read starting from the annotated 3'UTR start site to where the read ends (Step 1 in **Fig. 1**). After padding all the reads this way, we recalculate the read coverage on the 3′UTRs using 'bedtools genomecov' in Bedtools package[32] for each gene. Since the result

represents the full-length read coverage of the transcript in the 3′UTR, our novel padding step enables us to employ sensitive statistical approaches as follows.

Step 2. *Quantify 3'UTR long/short isoforms.*

For further quantification, we formulate an optimization problem to infer the proximal pA site. Since our transformation reveals the proximal pA site where the read coverage changes, the optimization problem is minimizing the difference between the accumulated density of the isoforms and the input RNA-Seq read coverage as follows.

$$(w_{kL}^*, w_{kS}^*, P_k^*) = \underset{w_{kL}^*, w_{kS}^* \geq 0, 1 < P_k < L}{\operatorname{argmin}} || R_{ki} - (w_{kL} I_{kL} + w_{kS} I_{kP}) ||_2^2$$

where w_{kL} and w_{kS} are the transcript abundances of long and short 3′-UTR isoforms for cell cluster k, respectively. $R_{ki} = [R_{ki1}, \dots, R_{kij}, \dots, R_{kiL}]^T$ is the corresponding read coverage at single-nucleotide resolution normalized by total sequencing depth. L is the length of the longest 3′-UTR length from annotation, P_k is the length of alternative proximal 3′-UTR to be estimated, I_{kL} is an indicator function with L times of 1, andI_{kP} has P_k times of 1 and $L - P_k$ times of 0. We solve this equation using quadratic programming [18] as was done in DaPars2. We will describe how this is extended to identify genes with more than two pA sites at the end of this section.

Step 3*. estimate APA significance across cell clusters.*

To make sure only genes with strong APA signals among multiple cell types are identified, we first filter out genes in which only 1 PA site is detected in less than 3 cell types. Then, for each gene, we calculate the CPM for long and short isoforms separately and average over all cell types. Only genes with an average CPM larger than 10 for both long and short isoforms are kept. In addition to gene-wise filtering, we also apply cell-wise filtering for each passed gene to keep

only cell types with at least 20 raw counts of reads in the model. For each gene, cell types with extremely low coverage (< 20) will not be used to estimate the APA status.

To model the relationship between the long/short isoform identified above and the given cell types, we build logistic regression for each gene with log-odds of the event that transcript uses distal polyA site (having long isoform) as the outcome and cell types as predictors using weighted effect coding scheme. When scRNA-Seq data were collected from multiple samples or individuals, scMAPA can be easily extended to control the effect of unmatched confounding factors by adding them into the regression model:

$$\ell = \ln \frac{p}{1-p} = \beta_0 + \sum_{i}^{n-1} \beta_i * C_i + \sum_{j}^{m} \beta_j * V_j$$

where $\frac{p}{1-p}$ is the odds of the transcript having a long isoform. β_i and C_i denote the coefficients and the binary indicator of each cell type, respectively. n is the number of cell types. Since one cell type needs to be chosen as a reference for model fitting, scMAPA fits the model twice to get the estimates of coefficients for all cell types. V_j and β_j denote the sample-specific binary confounding variables (e.g., clinical variable) and their coefficients, respectively. m is the number of confounding factors.

When there is no confounding factor, the likelihood ratio test (LRT) between cell type only model and null model is conducted to test the unadjusted effect of cell type, which is equivalent to the likelihood ratio chi-squared test of independence between long/short isoforms and cell types. With the existence of confounding variables, LRT between the full model and confounding variables only model is conducted to test the adjusted effect of cell type. P-values from all tests are further adjusted by the Benjamini–Hochberg (B-H) procedure to control the

false-discovery rate (FDR) at 5%. In addition, to ensure there is a significant change in effect size, the odds ratio of each cell type against the grand mean of all included cell types is calculated. There should be at least one cell type whose odds ratio is greater than 0.25 for a gene to be called an APA gene.

Currently, scMAPA assumes only 2 pA sites in the 3′-UTRs. However, our logistic model for step 2 can be easily extended to detect >2 peaks if employing other quantifiers that can consider >2 pA sites. For example, when only 2 peaks are detected for a gene, a binary logistic regression model would be fitted. However, when more than 2 peaks are detected for a gene, a multinomial logistic regression model would be fitted. To the best of our knowledge, since the only current tool that detects >2 peaks is scAPA, a multinomial logistic regression model is only compatible with the peak detection result of scAPA. LRT test is used to estimate the significance of APA among multiple peaks and cell types similarly.

Identification of cluster-specific 3′-UTR dynamics.

For the genes where significant APA dynamics is detected, scMAPA further analyses which cell type significantly contributes to the APA in which direction within each gene. By using a weighted effect coding scheme, each coefficient in the logistic regression can be interpreted as a measurement of deviation from the grand mean of all cells. This grand mean is not the mean of all cell type means, rather it is the estimate of the proportion of long isoforms of all cells for each gene. So, the unbalanced cell population sizes, which are common in scRNA-Seq would not affect the accuracy of estimation.

We use the following two criteria to determine the cluster-specific significant 3′-UTR dynamics:

First, given coefficients estimated from logistic regression, we use the Wald test to determine the p-value of each coefficient. P-values among all genes with significant APA of the same cell type are further adjusted by FDR. Then, we further selected genes whose APA degrees change greater than 2-fold. If the APA degree increases greater than 2-fold, the respective gene is considered as 3′-UTR lengthening. And, if the APA degree decreases less than 2-fold, the respective gene is considered as 3′-UTR shortening. However, users can define a different cutoff value of fold change to call 3′-UTR lengthening or shortening.

Identification of genes of more than two pA sites.

scMAPA can be easily extended to detect more than two pA sites and subsequently identify the significant differential usage of them. To detect more than two pA sites, scMAPA employs a similar approach to DaPars as follows. Instead of optimizing the regression model with a fixed number of predictors (proximal and distal pA sites), the case with more than 2 pA sites across n cell types can be formulated as follows.

$$\begin{bmatrix} r_{11} & r_{12} & \cdots & r_{1n} \\ r_{21} & r_{22} & \cdots & r_{2n} \\ \vdots & \vdots & \cdots & \vdots \\ r_{m1} & r_{m2} & \cdots & r_{mn} \end{bmatrix} = \begin{bmatrix} 1 & 1 & \cdots & 1 \\ 0 & 1 & \cdots & 1 \\ \vdots & \vdots & \cdots & \vdots \\ 0 & 0 & \cdots & 1 \end{bmatrix}_{m\times m} \begin{bmatrix} w_{11} & \cdots & w_{1n} \\ w_{21} & \cdots & w_{21} \\ \vdots & \cdots & \vdots \\ w_{m1} & \cdots & w_{mn} \end{bmatrix}_{m\times n}$$

where m is the length of the longest 3′-UTR of a transcript. w_{ij} is the estimated abundance of one possible 3′-UTR i in cell type j. Then, detecting multiple PA sites and estimating the abundance can be optimized by a LASSO regularization, in which the following equation should be optimized.

$$\underset{W}{\operatorname{argmin}} \frac{1}{2} || C - MW ||_2^2 + \lambda \|W\|_1$$

While the number of non-zero w_{ij} indicates the number of pA sites for this gene, scMAPA will consider the genes with up to four estimated non-zero w_{ij} by default that can be further changed by the user. While this would avoid overfitting, we expect the default value to allow us to capture most genes according to a recent study on the number of pA sites for genes[33].

After PA sites detection, the binomial logistic regression could be extended to a multinomial logistic regression to identify differential PA site usage when more than 2 PA sites exist. If in total P PA sites are detected by PA site detection module, the differential PA sites identification could be modeled as following,

$$\mathrm{Prob}(\mathrm{PA_i} = \mathrm{p}) = \frac{e^{\beta_p} \cdot X_i}{\sum_{k=1}^{P} e^{\beta_k} \cdot X_i}$$

where p is one of the P PA sites. X_i is a row vector of features of an observed transcript. β_p is the coefficients *associated with PA site p*.

Simulation

First, we used Splatter[34], a widely known scRNA-Seq simulator, to simulate the cell-level count matrix, which acts as the base of synthetic data. Splatter was trained by unfiltered mouse brain data and set to generate count matrices containing 5000 genes and 3000 cells. The matrix then collapsed into 5 columns, representing the total count of 5 cell groups. We call this 5000 × 5 matrix a cluster-level count matrix.

From the analyses of PBMC and mouse brain data, we found that the standard deviation of PDUI (percentage of distal polyA site usage, which is equivalent to the proportion of long isoforms) of

each gene could act as a classifier of APA gene and non-APA gene. Based on that, the standard deviation of PDUI for APA genes in synthetic data is estimated by calculating the mean of standard deviations of PDUI from APA genes detected by both scMAPA and scAPA from mouse brain data. Similarly, the standard deviation of PDUI for non-APA genes was estimated by calculating the mean of standard deviations of PDUI from genes identified as non-APA by both scMAPA and scAPA. With the estimated standard deviations, a PDUI matrix with the same size (5000 × 5) as the cluster-level count matrices was generated. Each row of the PDUI matrix has a standard deviation equal to either the estimated standard deviation for the APA gene or the non-APA gene. This is achieved by centering 5 randomly selected numbers from standard normal distribution to 0. Then multiply the desired standard deviation to these centered numbers and add them to the desired mean. The mean of each row was randomly picked from 0.05 to 0.95. Since the estimated $SD_{isoprop}$ values are averaged to 0.127 and 0.009 for the APA and the non-APA genes respectively, we generated simulation data with $SD_{isoprop}$ for APA genes in a range centered on 0.13 while fixing that for non-APAs at 0.009. The rows representing true APA genes were randomly selected. Then, each number in the cluster-level count matrix is divided into the count of long isoforms and the count of short isoforms by multiplying and PDUI matrix or (1-PDUI matrix), respectively. Finally, Pearson's chi-squared test (scAPA), logistic regression model + LRT (scMAPA) could be applied to assess the performance of these three methods. For each repeat of simulation, PDUI matrix is regenerated but the cluster-level count matrix keeps the same for the sake of computational burden. Every simulation design was repeated 100 times to derive summarized statistics.

To examine the impact of experimental design on statistical power to detect significant APA genes, we assess the performance of scMAPA and scAPA in the following aspects: 1) To test the

impact of unbalanced cell populations, the proportion of 5 cell types in the synthetic cell-level count matrices were set to three scenarios with different distribution of cell-type populations: (20%, 20%, 20%, 20%, 20%), (30%, 17.5%, 17.5%, 17.5%, 17.5%), and (50%, 12.5%, 12.5%, 12.5%, 12.5%). 2) To test the impact of the proportion of true APA genes, we set three levels of true APA proportions, 5%, 10%, and 20%. 3) To test the impact of the extent of APA dynamics, instead of using mean of standard deviations, we set the standard deviations of true APA genes in the simulated PDUI matrix to the 15 equally spaced sequence of numbers between the first quartile and the third quartile of standard deviations estimated from APA genes in mouse brain data. In total, there were 9 scenarios, corresponding to 9 combinations of factors 1) and 2). When testing factor 3), we chose balanced cell type proportion (0.2, 0.2, 0.2, 0.2, 0.2) and 10% true APA genes.

Assessing accuracy of PA site estimation

To assess the PA site/ peak interval prediction accuracy, we used peak lists or PA site lists from scMAPA, scAPA, and Sierra on PBMC data. The estimation accuracy is measured by the percentage of the predicted peaks or PA sites overlapped with PA sites annotated in PolyASite 2.0. Since it is meaningless to find the overlap between two-point estimates, we expanded the point position from the annotation database to an interval by manually adding a distance ranging from 10 bp to 150 bp in a 10 bp increment to both sides of the annotated PA sites. scMAPA gives a point estimate of PA site as predicted proximal PA site and Sierra gives two-point estimates as fit max position and max position. To make the comparison more comprehensive, we calculated the midpoint of peak interval as the pseudo point estimate of scAPA. The point estimates from these methods are considered as supported by the annotation database if the point position falls in the annotated interval (annotated PA site ± distance). For peak intervals

estimated by scAPA and Sierra, as long as there is 1 bp overlap between the estimated interval and the annotated interval (either start or end of estimated interval falls in annotated PA site ± distance), the estimate would be considered as supported by annotation database. Then, the percentage supported by annotation is calculated as the number of PA sites or peak intervals supported by the annotation database divided by total peaks detected for each method.

Running scDAPA, scAPA and Sierra

Sierra and scDAPA were run with default parameters. scAPA was run with default parameters and intronic regions omitted. The genes with a CPM of less than 10 were filtered out. We want to point out that scAPA employs chisq.test function in R to estimate the significance of dynamic PA sites usage among multiple clusters. This potentially makes the identification of scAPA much conservative than other tools in the multi-group setting since it does not allow any cell type to have 0 count, as R's chisq.test would return NA as p-value if there is 0 presented in the count table. However, it is common to observe that a few cell types would not express certain genes in scRNA-Seq, especially when the whole cell population is split into more than 5 clusters (cell types), which is typical for complex biological systems.

To compare scDAPA and Sierra with scAPA and scMAPA in multiple-cluster settings, since scDAPA and Sierra identify APA genes only between cell cluster pairs, we combined the pairwise significant APA genes in each method separately. After controlling FDR on the combined APA genes, we called APA genes if they are significant in any of the pairwise identifications.

Controlling undesired source of variance in cell-type-specific identification of APA genes

To compare the running modes, we first divided the mouse brain data into 10 cell groups by cell type and brain region (5 cell types × 2 brain regions). In each data, we quantified the APA isoforms using scMAPA in two running modes, referred to as brain-region-confounding/controlled in the main text. The brain-region-confounding model is formulated as

$APA\_Isoform \sim cell\_type$.

And the brain-region-controlled model is formulated as

$APA\_Isoform \sim cell\_type$+brain_region.

Availability of supporting source code and requirements

Project name: scMAPA

Project home page: https://github.com/ybai3/scMAPA

RRID: SCR_021822

biotoolsID: biotools:scmapa

Operating system: Platform independent

Programming language: R

License: GNU GPL

DECLARATIONS

Competing interests The authors declare no competing financial interests.

Funding This work was supported partly by the Joan Gollin Gaines Cancer Research Fund at the University of Pittsburgh to H.J.P.. This project used the UPMC Hillman Cancer Center Biostatistics Shared Resource that is supported in part by award P30CA047904.

Author Contributions H.J.P and Y.B. conceived the project, designed the experiments. Y.B. and Z.F. implemented the software. Y.B., Y.Q., R.M. performed the analysis. S.K., K.N., H.M.Z., R.K., Q.P. interpreted the results statistically and/or biologically.

Acknowledgements We thank Daniel Weeks, Ph.D., Professor, Department of Human Genetics, University of Pittsburgh for valuable discussion. This research was supported in part by the University of Pittsburgh Center for Research Computing through the resources provided. We also acknowledge the authors of scAPA for their generous provision of their data.

REFERENCES

[1] A. Derti *et al.*, "A quantitative atlas of polyadenylation in five mammals.," *Genome Res.*, vol. 22, no. 6, pp. 1173–1183, Jun. 2012, doi: 10.1101/gr.132563.111.

[2] C. P. Masamha *et al.*, "CFIm25 links alternative polyadenylation to glioblastoma tumour suppression.," *Nature*, vol. 510, no. 7505, pp. 412–416, May 2014, doi: 10.1038/nature13261.

[3] L. C. Cheng *et al.*, "Widespread transcript shortening through alternative polyadenylation in secretory cell differentiation," *Nat. Commun.*, vol. 11, no. 1, p. 3182, 2020, doi: 10.1038/s41467-020-16959-2.

[4] M. Chen *et al.*, "3 ′ UTR lengthening as a novel mechanism in regulating cellular

senescence,” *Genome Res.*, vol. 28, no. 3, pp. 285–294, 2018, doi: 10.1101/gr.224451.117.Freely.

[5] C. Ye *et al.*, “scDAPA: detection and visualization of dynamic alternative polyadenylation from single cell RNA-seq data,” *Bioinformatics*, vol. 36, no. 4, pp. 1262–1264, 2020, doi: 10.1093/bioinformatics/btz701.

[6] R. Patrick *et al.*, “Sierra: discovery of differential transcript usage from polyA-captured single-cell RNA-seq data,” *Genome Biol.*, vol. 21, no. 1, p. 167, 2020, doi: 10.1186/s13059-020-02071-7.

[7] E. D. Shulman and R. Elkon, “Cell-type-specific analysis of alternative polyadenylation using single-cell transcriptomics data,” *Nucleic Acids Res.*, vol. 47, no. 19, pp. 10027–10039, 2019, doi: 10.1093/nar/gkz781.

[8] S. Heinz *et al.*, “Simple combinations of lineage-determining transcription factors prime cis-regulatory elements required for macrophage and B cell identities,” *Mol. Cell*, vol. 38, no. 4, pp. 576–589, May 2010, doi: 10.1016/j.molcel.2010.05.004.

[9] A. H. Shih *et al.*, “Mutational Cooperativity Linked to Combinatorial Epigenetic Gain of Function in Acute Myeloid Leukemia,” *Cancer Cell*, vol. 27, no. 4, pp. 502–515, 2015, doi: https://doi.org/10.1016/j.ccell.2015.03.009.

[10] M. Jung *et al.*, “GATA2 deficiency and human hematopoietic development modeled using induced pluripotent stem cells.,” *Blood Adv.*, vol. 2, no. 23, pp. 3553–3565, Dec. 2018, doi: 10.1182/bloodadvances.2018017137.

[11] A. Zeisel *et al.*, “Molecular Architecture of the Mouse Nervous System.,” *Cell*, vol. 174,

no. 4, pp. 999-1014.e22, Aug. 2018, doi: 10.1016/j.cell.2018.06.021.

[12] R. Bohnert and G. Rätsch, "rQuant.web: a tool for RNA-Seq-based transcript quantitation.," *Nucleic Acids Res.*, vol. 38, no. Web Server issue, pp. W348-51, Jul. 2010, doi: 10.1093/nar/gkq448.

[13] L. Li *et al.*, "An atlas of alternative polyadenylation quantitative trait loci contributing to complex trait and disease heritability," *Nat. Genet.*, vol. 53, no. July, 2021, doi: 10.1038/s41588-021-00864-5.

[14] C. J. Herrmann, R. Schmidt, A. Kanitz, P. Artimo, A. J. Gruber, and M. Zavolan, "PolyASite 2.0: a consolidated atlas of polyadenylation sites from 3′ end sequencing," *Nucleic Acids Res.*, vol. 48, no. D1, pp. D174–D179, Jan. 2020, doi: 10.1093/nar/gkz918.

[15] C. Ye, Q. Zhou, Y. Hong, and Q. Q. Li, "Role of alternative polyadenylation dynamics in acute myeloid leukaemia at single-cell resolution.," *RNA Biol.*, vol. 16, no. 6, pp. 785–797, Jun. 2019, doi: 10.1080/15476286.2019.1586139.

[16] W. D. E., W. A. J., G. A. Pathak, J. F. L., and W. I. L., "Physiological Migration of Hematopoietic Stem and Progenitor Cells," *Science (80-.).*, vol. 294, no. 5548, pp. 1933–1936, Nov. 2001, doi: 10.1126/science.1064081.

[17] Y. Xiang *et al.*, "Comprehensive Characterization of Alternative Polyadenylation in Human Cancer," vol. 110, no. November 2017, pp. 1–11, 2018, doi: 10.1093/jnci/djx223.

[18] Z. Xia *et al.*, "Dynamic Analyses of Alternative Polyadenylation from RNA- Seq Reveal Landscape of 3 ' UTR Usage Across 7 Tumor Types," *Nat. Commun.*, pp. 1–38, 2014.

[19] P. J. Shepard, E.-A. Choi, J. Lu, L. A. Flanagan, K. J. Hertel, and Y. Shi, "Complex and

dynamic landscape of RNA polyadenylation revealed by PAS-Seq.," *RNA*, vol. 17, no. 4, pp. 761–772, Apr. 2011, doi: 10.1261/rna.2581711.

[20] V. Hilgers, M. W. Perry, D. Hendrix, A. Stark, M. Levine, and B. Haley, "Neural-specific elongation of 3' UTRs during Drosophila development.," *Proc. Natl. Acad. Sci. U. S. A.*, vol. 108, no. 38, pp. 15864–15869, Sep. 2011, doi: 10.1073/pnas.1112672108.

[21] Z. Ji, J. Y. Lee, Z. Pan, B. Jiang, and B. Tian, "Progressive lengthening of 3' untranslated regions of mRNAs by alternative polyadenylation during mouse embryonic development.," *Proc. Natl. Acad. Sci. U. S. A.*, vol. 106, no. 17, pp. 7028–7033, Apr. 2009, doi: 10.1073/pnas.0900028106.

[22] A. Feiglin, B. K. Allen, I. S. Kohane, and S. W. Kong, "Comprehensive Analysis of Tissue-wide Gene Expression and Phenotype Data Reveals Tissues Affected in Rare Genetic Disorders," *Cell Syst.*, vol. 5, no. 2, pp. 140-148.e2, 2017, doi: 10.1016/j.cels.2017.06.016.

[23] K. A. Wanke, P. Devanna, and S. C. Vernes, "Understanding Neurodevelopmental Disorders: The Promise of Regulatory Variation in the 3′UTRome," *Biol. Psychiatry*, vol. 83, no. 7, pp. 548–557, 2018, doi: https://doi.org/10.1016/j.biopsych.2017.11.006.

[24] M. Göpferich *et al.*, "Single cell 3'UTR analysis identifies changes in alternative polyadenylation throughout neuronal differentiation and in autism," *bioRxiv*, p. 2020.08.12.247627, Jan. 2020, doi: 10.1101/2020.08.12.247627.

[25] C. Ye, Y. Long, G. Ji, Q. Q. Li, and X. Wu, "APAtrap: identification and quantification of alternative polyadenylation sites from RNA-seq data," doi: 10.1093/bioinformatics/bty029.

[26] A. Arefeen, J. Liu, X. Xiao, and T. Jiang, "TAPAS : tool for alternative polyadenylation site analysis," *Bioinformatics*, vol. 34, no. February, pp. 2521–2529, 2018, doi: 10.1093/bioinformatics/bty110.

[27] S. Picelli, O. R. Faridani, A. K. Björklund, G. Winberg, S. Sagasser, and R. Sandberg, "Full-length RNA-seq from single cells using Smart-seq2.," *Nat. Protoc.*, vol. 9, no. 1, pp. 171–181, Jan. 2014, doi: 10.1038/nprot.2014.006.

[28] Y. Hao *et al.*, "Integrated analysis of multimodal single-cell data.," *Cell*, vol. 184, no. 13, pp. 3573-3587.e29, Jun. 2021, doi: 10.1016/j.cell.2021.04.048.

[29] X. Zhang *et al.*, "CellMarker: a manually curated resource of cell markers in human and mouse," *Nucleic Acids Res.*, vol. 47, no. D1, pp. D721–D728, Jan. 2019, doi: 10.1093/nar/gky900.

[30] T. Smith, A. Heger, and I. Sudbery, "UMI-tools: Modeling sequencing errors in Unique Molecular Identifiers to improve quantification accuracy," *Genome Res.*, vol. 27, no. 3, pp. 491–499, 2017, doi: 10.1101/gr.209601.116.

[31] M. V. Kuleshov *et al.*, "Enrichr: a comprehensive gene set enrichment analysis web server 2016 update," *Nucleic Acids Res.*, vol. 44, no. W1, pp. W90–W97, 2016, doi: 10.1093/nar/gkw377.

[32] A. R. Quinlan, *BEDTools: The Swiss-Army tool for genome feature analysis*, vol. 2014. 2014.

[33] B. J. Harrison *et al.*, "Detection of Differentially Expressed Cleavage Site Intervals Within 3′ Untranslated Regions Using CSI-UTR Reveals Regulated Interaction Motifs ,"

Frontiers in Genetics , vol. 10. p. 182, 2019, [Online]. Available: https://www.frontiersin.org/article/10.3389/fgene.2019.00182.

[34] L. Zappia, B. Phipson, and A. Oshlack, “Splatter: simulation of single-cell RNA sequencing data,” *Genome Biol.*, vol. 18, no. 1, p. 174, 2017, doi: 10.1186/s13059-017-1305-0.

S. Table 1. Cell type annotation based on marker genes curated in CellMarker[20] for 10k, 5k, and 1k in the PBMC data.

S. Table 2. Detailed information of APA genes detected by scMAPA, scAPA, scDAPA, and Sierra on the PBMC data including Ingenuity Pathway Analysis (IPA) analysis result.

S. Table 3. scMAPA estimation result for APA genes on the mouse brain data.

S. Table 4. Result of IPA comparison analysis on the “Disease & Function” terms enriched for APA genes identified uniquely by scAPA, scMAPA, and commonly by both on the mouse brain data (1,446, 2,175, and 1,048 respectively).

S. Table 5. Result of IPA comparison analysis on the “Disease & Function” terms enriched for APA genes identified uniquely in astrocyte, immune, oligos, vascular, and neuron cells.

S. Table 6. scMAPA estimates on the input data that are split by cell type and brain region either with brain region as a undesired source of variation or not.

S. Table 7. IPA upstream regulator analysis result (enrichment p-value) on 113 and 2,715 APA genes that are supposed to be brain-region-specific and non-specific, respectively.

Figure Click here to access/download;Figure;Fig1.docx

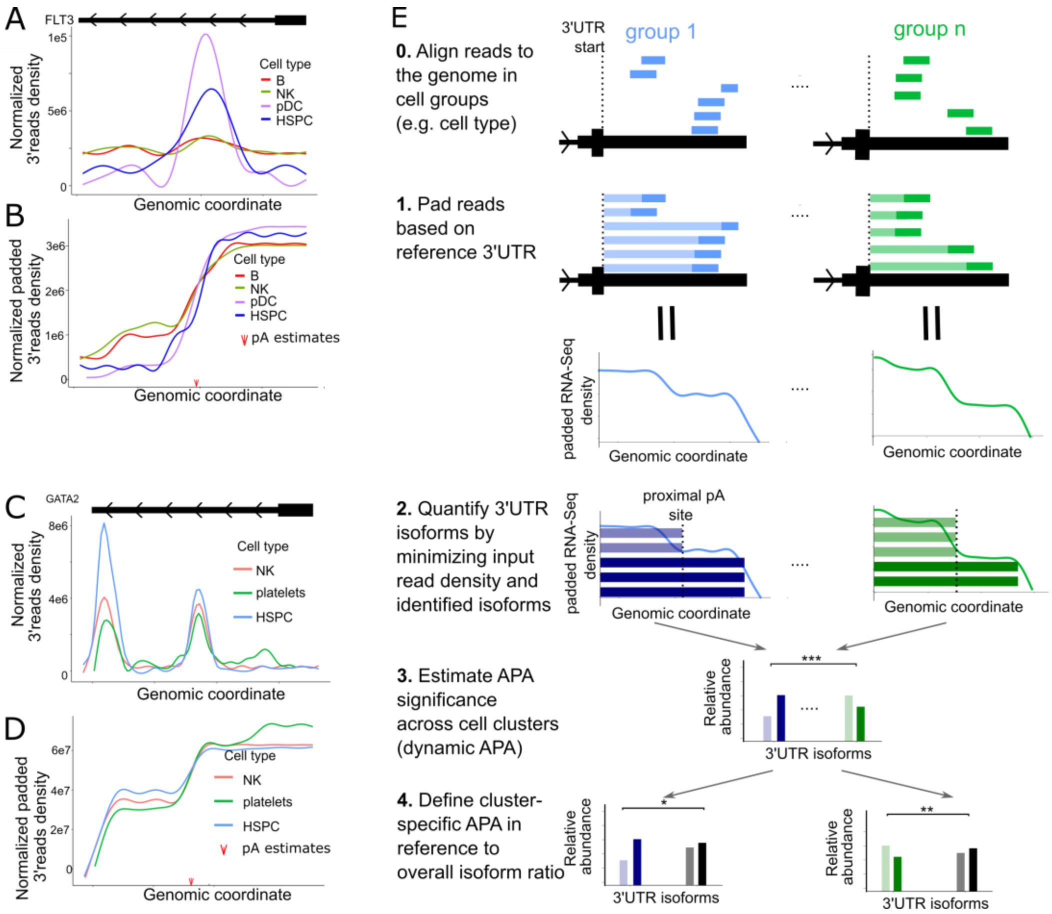

Figure 1. Motivation and schematic illustration of scMAPA. (A) The read density shape on the FLT3 3′-UTR in multiple cell types of 10k PBMC scRNA-Seq data. (B) The transformed read density shape on the FLT3 3′-UTR in multiple cell types of 10k PBMC scRNA-Seq data. The red arrow indicates the proximal polyA site predicted. (C) The read density shape on the GATA2 3′-UTR in multiple cell types of 10k PBMC scRNA-Seq data. (D) The transformed read density shape on the GATA2 3′-UTR in multiple cell types of 10k PBMC scRNA-Seq data. The red arrow indicates the proximal polyA site predicted. (E) In Step 0 and 1, bars in solid color represent 3′ biased scRNA-Seq reads and bars in light color indicate how the 3′ biased reads are padded from the 3′ start site to the end of the read to represent the full-length 3′ UTR of the transcript. In Step 2, the blue and green bars indicate the estimated isoforms in each cell type, where solid and light coloring mode indicate 3′ UTR long and short isoforms. In Step 3 and 4, the bars represent the estimated number of APA isoforms in each cell type.

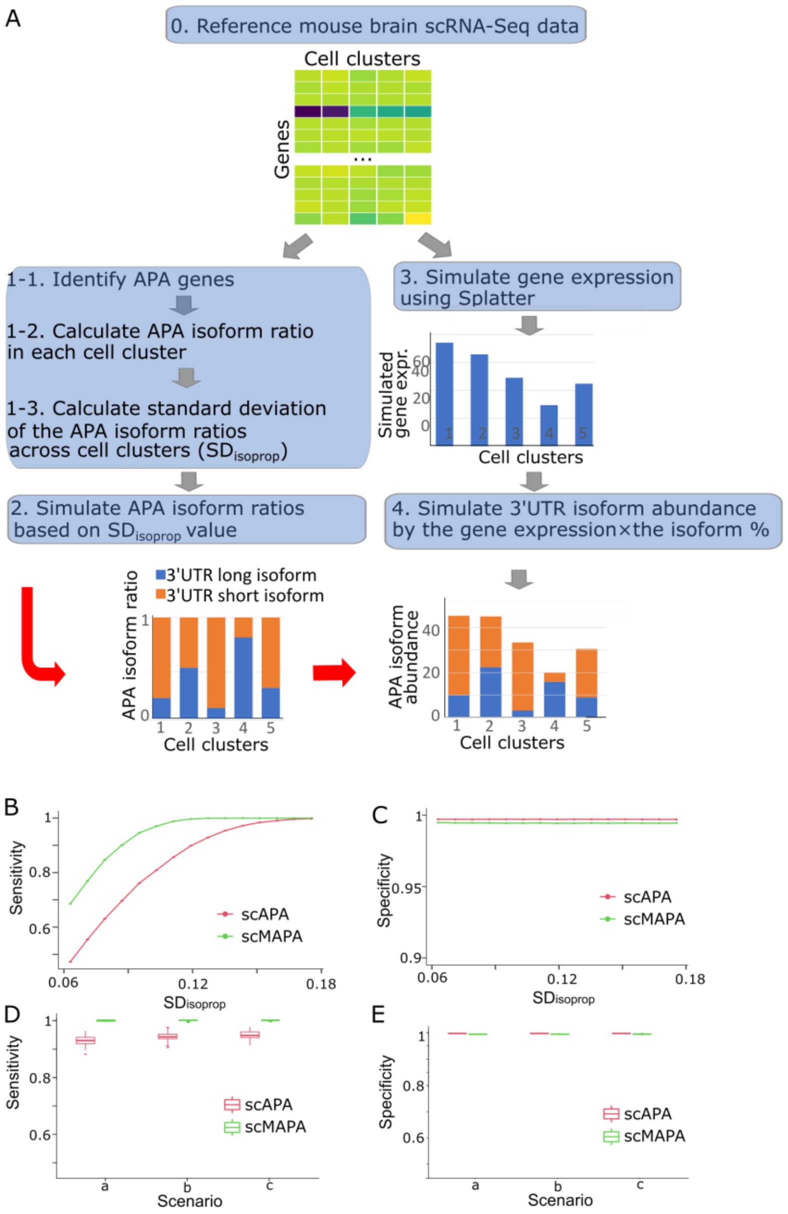

Figure 2. Performance assessment on the statistical component of scMAPA and scAPA using simulated data. With fixed number of true APA events (500 out of 5000) and uniform distribution of cell cluster size (600 cells in each cell type) (A). Illustration of the simulation process. Genes identified as significant APA genes by both scMAPA and scAPA were considered as APA genes. Genes identified as non-significant APA genes by both methods were considered as non-APA genes. (B) Comparison of scMAPA vs. scAPA in terms of sensitivity. We varied the standard deviation (SD) of APA isoforms across clusters ($SD_{isoprop}$) for 500 true APA genes (0.06 to 0.18) with the fixed $SD_{isoprop}$ value for 4,500 non-APA genes (0.009). (C) Comparison of scMAPA vs. scAPA in terms of specificity in the same scenario. (D) Comparison of scMAPA vs. scAPA in terms of sensitivity. We varied cell cluster size: (20%, 20%, 20%, 20%, 20%) for scenario a, (30%, 17.5%, 17.5%, 17.5%, 17.5%) for b, and (50%, 12.5%, 12.5%, 12.5%, 12.5%) for c.

 Click here to access/download;Figure;Fig3.docx

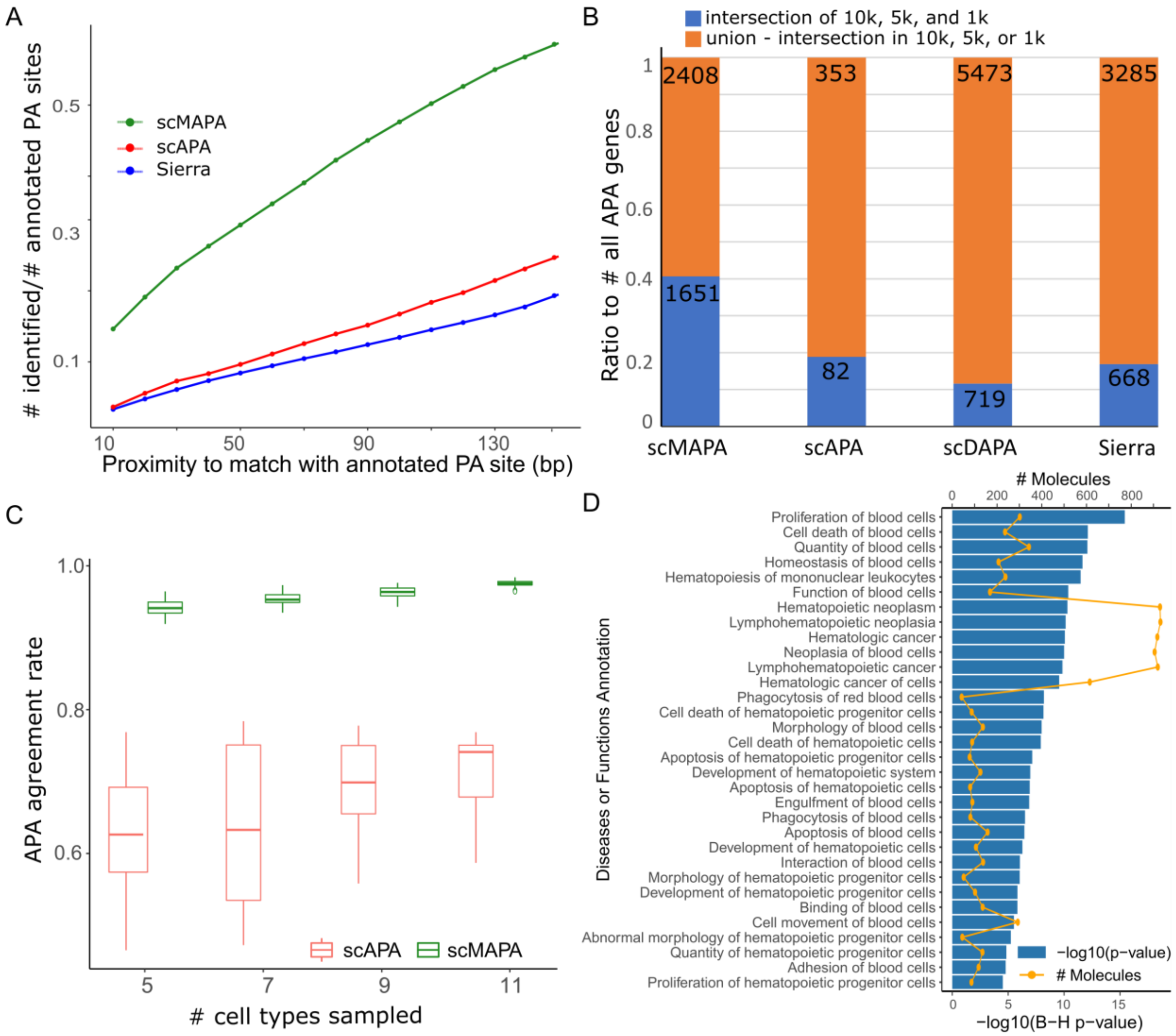

Figure 3. Performance assessment of scMAPA, scAPA, scDAPA, and Sierra using PBMC data. (A) The ratio of annotated pA sites identified by scMAPA vs. scAPA and Sierra on the PBMC 10k data. The identified pA sites were deemed annotated when they are within a range to any annotated pA sites while the range was set from 10 bp to 130 bp, respectively. We extracted the annotated pA sites from PolyASite 2.0. (B) The ratio of significant APA genes found in all three PBMC data (10k, 5k, and 1k) in blue bar and in any combination but all three in orange by scMAPA, scAPA, scDAPA, and Sierra (C) Box plots showing the proportion of the overlap between sample APA genes and total APA genes normalized to total APA genes (APA agreement ratio). The APA agreement ratio values were evaluated in various numbers of cell types sampled. (D) Significance of enrichment (blue bar) and number of overlaps (orange line) of 3,574 scMAPA APA genes on IPA Disease and Function terms with the keyword “blood” or “hematopoiesis”.

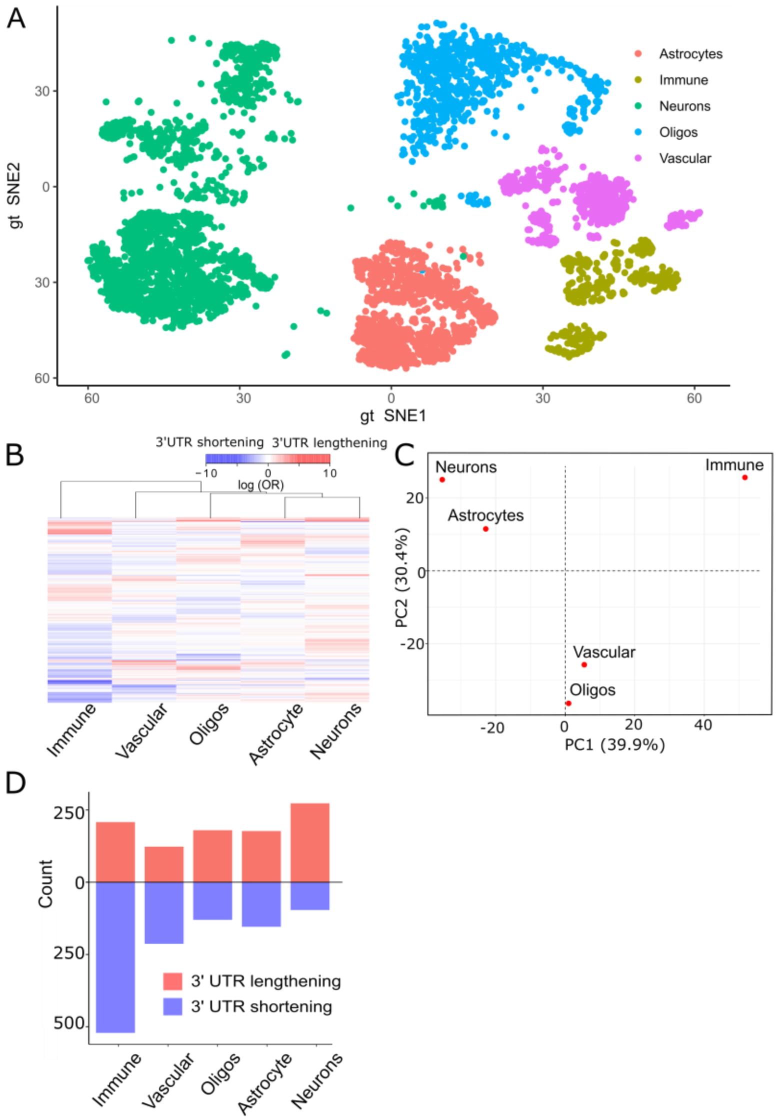

Figure 4. A novel module of scMAPA cell-type-specific APA identification on the mouse brain data. (A) tSNE plot showing the cell types of the mouse brain scRNA-Seq data. (B) Heatmap of the APA effect sizes estimated for each cell type, representing the coefficients in the scMAPA logistic regression model. (C) PCA plot showing how the cell types are similar or dissimilar in the APA effect size. PC1 and PC2 together account for 70.3% of the variation. (D) Bar plot showing the number of significant 3′-UTR lengthening (red) and shortening (blue) identified in each cell type.

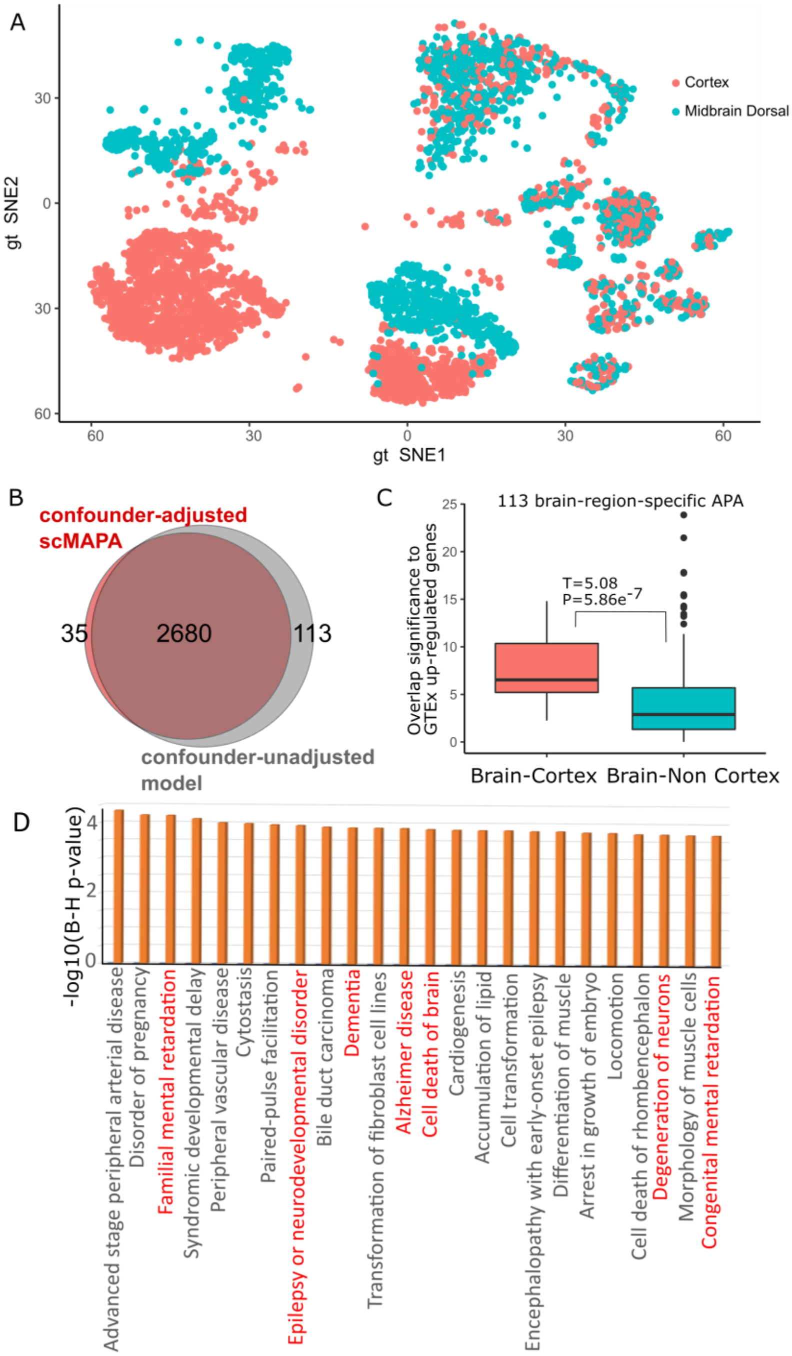

Figure 5. (A) tSNE plot showing the brain region of the mouse brain scRNA-Seq data. (B) Venn diagram showing the APA genes identified by the confounder-adjusted scMAPA and the confounder-unadjusted model. (C) Box plot showing significance of overlap between the 113 genes and the up-regulated genes in GTEx brain samples whether they are from cortex (red) or not (green). (D) Significance (B-H p-value) of IPA enrichment terms that are uniquely and significantly (B-H p-value<10^{-2}) enriched to 2,793 confounder-adjusted scMAPA.

Supplementary Figures

Click here to access/download
Supplementary Material
ver24_SFigures.docx

Supplementary Table 1

Click here to access/download
Supplementary Material
STable1_Supplementary Material.xlsx

Supplementary Table 2

Click here to access/download
Supplementary Material
STable2_Supplementary Material.xlsx

Supplementary Table 3

Click here to access/download
Supplementary Material
STable3_Supplementary Material.xlsx

Supplementary Table 4

Click here to access/download
Supplementary Material
STable4_Supplementary Material.xlsx

Supplementary Table 5

Click here to access/download
Supplementary Material
STable5_Supplementary Material.xlsx

Supplementary Table 6

Click here to access/download
Supplementary Material
STable6_Supplementary Material.xlsx

Supplementary Table 7

Click here to access/download
Supplementary Material
STable7_Supplementary Material.xlsx

Marked version of the updated manuscript

Click here to access/download
Supplementary Material
ver25_marked.pdf

Cover letter and our responses to the reviewers
Click here to access/download;Personal Cover;ReviewerComments.docx

University of Pittsburgh
Graduate School of Public Health
Department of Human Genetics

Hyun Jung Park, Ph.D.
Assistant Professor
Department of Human Genetics
Department of Biostatistics
School of Public Health
Department of Biomedical Informatics
University of Pittsburgh
E-mail: hyp15@pitt.edu
http://www.parklab.pitt.edu/

Nov. 18th, 2021
Hans Zauner, Ph.D.
GigaScience

Dear Dr. Zauner,

Enclosed please find marked and unmarked versions of our revised manuscript, entitled I would like to submit our manuscript, " *scMAPA: Identification of Cell-type-specific Alternative Polyadenylation in Complex Tissues*", as well as our detailed responses to the reviewers' comments (see below). In response to your suggestion, we made RRID and biotoolsID (SCR_021822 and biotools:scmapa, respectively) for scMAPA and included this information under **Availability of supporting source code and requirements** in the manuscript.

We thank you and the reviewers for helpful comments, which have substantially strenghtned our report.

Thanks for considering our revised manuscripts for publication in GigaScience.

Sincerely yours,

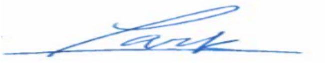

Hyun Jung Park, Ph.D.
Assistant Professor of Human Genetics
University of Pittsburgh

RESPONSE TO REVIEWERS' CRITIQUES

Reviewer reports:
Reviewer #1: The authors have addressed some of my concerns but not others. The remaining major issue is that the definition of 'confounding factor' is quite confusing. The authors are recommended to provide a more intelligible description.

Response: We realize that the word "confounding" can mean differently by context, potentially confusing readers. Thus, we removed the word and rewrote those parts with the specific meaning of it, which is "undesired source of variation". To clarify further, we kept our focus on a specific case where brain region information becomes the undesired source of variation in identifying brain cell-type-specific APA genes throughout the manuscript.

Reviewer #2:
The authors present a novel tool scMAPA for the identification and quantification of alternative poly-adenylation sites from scRNA-seq.

The manuscript has had a substantial re-write and additional analyses performed since the previous submission. It has been improved significantly and previous comments addressed.

Response: Thank you so much for acknowledging our effort to improve our manuscript.

Major comments

In the Findings section there is too much methodology mentioned in it but without the detail so makes reading it harder. In the Findings section a focus should be on the results such as the comparison to Sierra and scAPA and what was identified in the PBMC and Mouse data.
Response: Thanks to this reviewer's comment, we moved the descriptions about methodology to Methods section. Previously, there were 2,689 words in Findings section. This will make our manuscript to be more understable. Thank you for your comment.

The authors need to also ensure the past tense is used consistently throughout. An example is:
p.13 para 2 line 14 "we test if they express highly"
is better as
"we tested if they were expressed highly"
Response: Thank you for your suggestion. We made this correction the reviewer suggested. Also, we made the following corrections we found in the same direction.

1. P.5 line 9, "this transformation made the APA short and long isoforms readily distinguishable" -> "this transformation makes the APA short and long isoforms readily distinguishable"
2. P.6 line 13, "which used the quadratic programming"-> "which uses the quadratic programming"
3. P.8 line 6, "the mouse brain data showed a narrow range"-> "the mouse brain data show a narrow range"
4. P.9 line 1, "scMAPA consistently outperformed the other methods"-> "scMAPA consistently outperforms the other methods"
5. P.9 line 13, "scMAPA identified a two-fold higher percentage of APA genes"-> "scMAPA identifies a two-fold higher percentage of APA genes"

6. P.10 line 17, “showed the dynamic APA isoform ratios across the cell types”-> “show the dynamic APA isoform ratios across the cell types”
7. P.11 line 4, “While both the analyses supported the previous finding” -> “While both the analyses support the previous finding”
8. P.11 line 15, “they further revealed that immune cells are most different from all the other cell types”-> “they further reveal that immune cells are most different from all the other cell types”
9. P.13 line 2, “APA genes associated with a brain region can be mistakenly identified as cell-type-specific APA genes”-> “APA genes associated with a brain region could be mistakenly identified as cell-type-specific APA genes”
10. P.13 line 18, “GTEx does not collect the expression data for midbrain” -> “GTEx did not collect the expression data for midbrain”

On p. 9 para 1 line 4 different numbers of cells were defined: "6, 8, and 13 types for 1k, 5k, and 10k data respectively"
How representative are those numbers? There are 5x more cells in the 5k data than 1k yet only a third more cells types. The 5k vs 10k are more consistent: 2x cells and ~1.5x types.
Response: We determined the number of clusters by running a well-established algorithm. Especially, we used the parameters suggested particular for the 1k, 5k, and 10k data [1]. To further validate the number of the clusters, we examined the percentage of variance explained (between-group variance/total variance) against the different number of clusters in elbow plot analysis (R. Fig. 1A, B, C for 1k, 5k, and 10k data respectively). From the elbow plots, we can see that the number of clusters was set in an acceptable range of the explained variance (between the steepest increase and the flattening point), suggesting that Seurat’s method delineated an appropriate number of clusters in the 1k, 5k, and 10k data. Especially, although 5x more cells in the 5k data did not proportionally increase the number of clusters from the 1k data, the defined clusters explain a very similar percentage of the variance (~16.25%), supporting the number of clusters in the 1k and 5k data again.

Another support comes when checking the dimension-reduced space (UMAP) of the data (R. Fig. 1D, E, F for 1k, 5k, and 10k data respectively), since distinct cell types are expected to be well separated on the UMAP. Since it is the case for the 1k, 5k, 10k data, we believe that the numbers of the defined clusters were set appropriately.

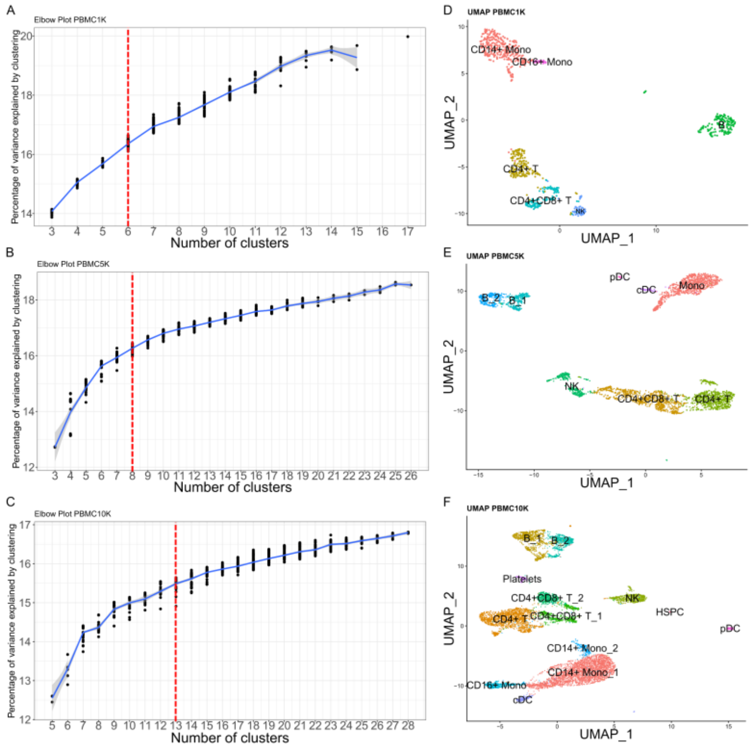

R. Figure 1. Percentage of variance explained by different numbers of clustering for PBMC 1k (A), 5k (B), and 10k (C) data where red dotted lines indicate the number of clusters we decided to use. UMAP presentation of the 1k (D), 5k (E), and 10K (F) data.

p. 10 para 1 line 3. scMAPA found 40.7% genes as being APA compared to the other tools which found between 11.6-18.9%. Do the authors know whether that is a biological valid percentage?
Response: To identify a valid percentage in our experiment investigating how much APA genes are identified in different numbers of cells sampled, we ran scMAPA on the 1k and 10k data to find that 51.5% of the APA genes are common. Since the data were sampled from a same healthy donor, the number of APA genes common to the 1k and 10k data indicates the upper bound of the identification attributing mainly to the different number of cells in the data, and thus representing a biologically valid percentage for our experiment. Since 40.7% of genes commonly being APA is lower than this percentage, we believe that the results are valid, suggesting that scMAPA is most robust to the sample size changes.

Separately, to identify a biologically valid percentage in general, we searched literature reporting the number of APA events. Using a sequencing technique designed to comprehensively map polyadenylation sites, a recent study found that 78.5% of mRNA genes were found to undergo APA events on the RNA samples across a more diverse set of samples ((i) male and female whole bodies; (ii) embryos at 11, 15 and 17 days (d); (iii) brain and testis tissues at different postnatal stages; and (iv) over 11 cell lines[2]).

p. 10 para 2 line 4: "enrichments to 32 IPA terms that are characterized with keywords "blood" and "hematology", suggesting that the APA genes identified by scMAPA can play important roles in PBMC biology".
The PBMC dataset is a blood cell dataset which one would be always enriched with the terms "blood" and "hematology". Is that not so? How is the IPA returning enrichment for these terms a measure of scMAPA accuracy?

The reviewer is right that any functional component in the blood cells is expected to be enriched with keywords “blood” and “hematology”. Based on this rationale, we wanted to see if the identified APA genes implicate any functions in the blood cells by inspecting their enrichment terms. To conduct this analysis stringently and reduce the chance of random enrichment for the keywords, we “set the 18,804 genes expressed in the data as the background” in the analysis, so, for the APA genes to be significantly enriched with the “blood” and “hematology” terms, the enrichment degree should exceed what could be expected generally from the expressed genes in the PBMC data.

Also, the reviewer is right that these enrichments couldn’t be a measure of scMAPA accuracy. That is why we did not claim scMAPA accuracy with this finding, but to suggest “that the APA genes identified by scMAPA can play important roles in PBMC biology”.

p. 11 para 1 line 9: "Since bone marrow is developmentally related to peripheral blood, GATA2 may undergo the APA event in the PBMC under similar molecular mechanisms."
This statement needs to be supported with further evidence or the authors should say this is speculation.

Response: We changed the text in the manuscript as follows. “Since hematopoietic stem and progenitor cells (HSPC in Fig. 1C, D) are originated from bone marrow[3], we speculate that the molecular mechanisms rendering the APA event on GATA2 in the bone marrow mononuclear cells cause GATA2 to show different APA patterns than other cells in the PBMC.”

Minor comments

Abstract: para 2, line 3: "To release the assumptions" should be "To avoid the assumptions"
Response: This has been changed according to your suggestion.

p-values reported in scientific notation should be in the form 2.2 x 10^-16 not 2.2e^-16 as reported on p. 7 para 2 line 15. Also 10^-2 (p. 10 para 2 line 5) is better as 0.01
Response: We changed "p<2.2e^-16" into "p-value<2.2×10^{-16}". Also, we changed "B-H p<10^-2" to "0.01". Further, para 3 had two occurrences of "B-H P-val < 0.05". We changed them into "B-H p-val < 0.05" to make the same style. Several other places had similar issues. We changed them into the same style.

p. 10 para 2 line 4: spell out acronyms the first time they're used: B-H as Benjamini-Hochberg
Response: This has been changed according to your suggestion.

p. 11 para 1 line 10: "biologically reasonable APA genes" should be "biologically relevant APA genes"
Response: This has been changed according to your suggestion.

p. 14 para 2 line 9: is "10^3.5" what is meant here? Re-write in proper scientific notation as mentioned above.
Response: We changed it into "p-value < 2.2×10^{-4}".

p. 22 para 1 line 6: "at least 20 raw counts" of what?
Response: It is "at least 20 raw counts of reads". With this change, we updated that part as follows. "In addition to gene-wise filtering, we also apply cell-wise filtering for each passed gene to keep only cell types with at least 20 raw counts of reads in the model. For each gene, cell types with extremely low coverage (< 20) will not be used to estimate the APA status."

p. 24 para 2 line 4: "than ln(2), corresponding to a 2-fold change in odds ratio" is incorrect as ln() is the natural log so ln(2) equals 0.693
Response: We used logistic regression where ln(2) corresponds to a 2-fold change in odds ratio. However, we understand that this might confuse readers. So, we changed the text as follows. "we further selected genes whose APA degrees change greater than 2-fold. If the APA degree increases greater than 2-fold, the respective gene is considered as 3′-UTR lengthening. And, if the APA degree decreases less than 2-fold, the respective gene is considered as 3′-UTR shortening. However, users can define a different cutoff value of fold change to call 3′-UTR lengthening or shortening."

p. 24 para 3 line 1: "scMAPA can be easily extended" is better as "scMAPA has been extended".
Response: Although we decided not to do the extension in this manuscript for fair comparisons with other methods and for effective investigation of multiple cell types (see 3rd para in Discussion), we agree with the reviewer that this extension will be helpful for further analyses. We will work on this extension as future work. We made this point more specific in the 3rd para in Discussion.

References

1. Seurat for PBMC [Internet]. Available from: https://satijalab.org/seurat/articles/pbmc3k_tutorial.html

2. Hoque M, Ji Z, Zheng D, Luo W, Li W, You B, Park JY, Yehia G, Tian B. Analysis of alternative

cleavage and polyadenylation by 3′ region extraction and deep sequencing. Nat Methods. 2013;10(2):133–139.

3. E. WD, J. WA, Pathak GA, L. JF, L. WI. Physiological Migration of Hematopoietic Stem and Progenitor Cells. Science (80-). American Association for the Advancement of Science; 2001 Nov 30;294(5548):1933–1936.

4. Elkon R, Ugalde AP, Agami R. Alternative cleavage and polyadenylation : extent, regulation and function. Nat Rev Genet. Nature Publishing Group; 2013;14(7):496–506.
