# Supplementary material for: scMAPA: Identification of cell-type–specific alternative polyadenylation in complex tissues
Source: Gigascience. 2022 Apr 30;11:giac033. doi: 10.1093/gigascience/giac033 (PMC9055853; doi:10.1093/gigascience/giac033)
Supplement: giac033_Supplemental_Files [file giac033_supplemental_files.zip › ver24_SFigures.docx]

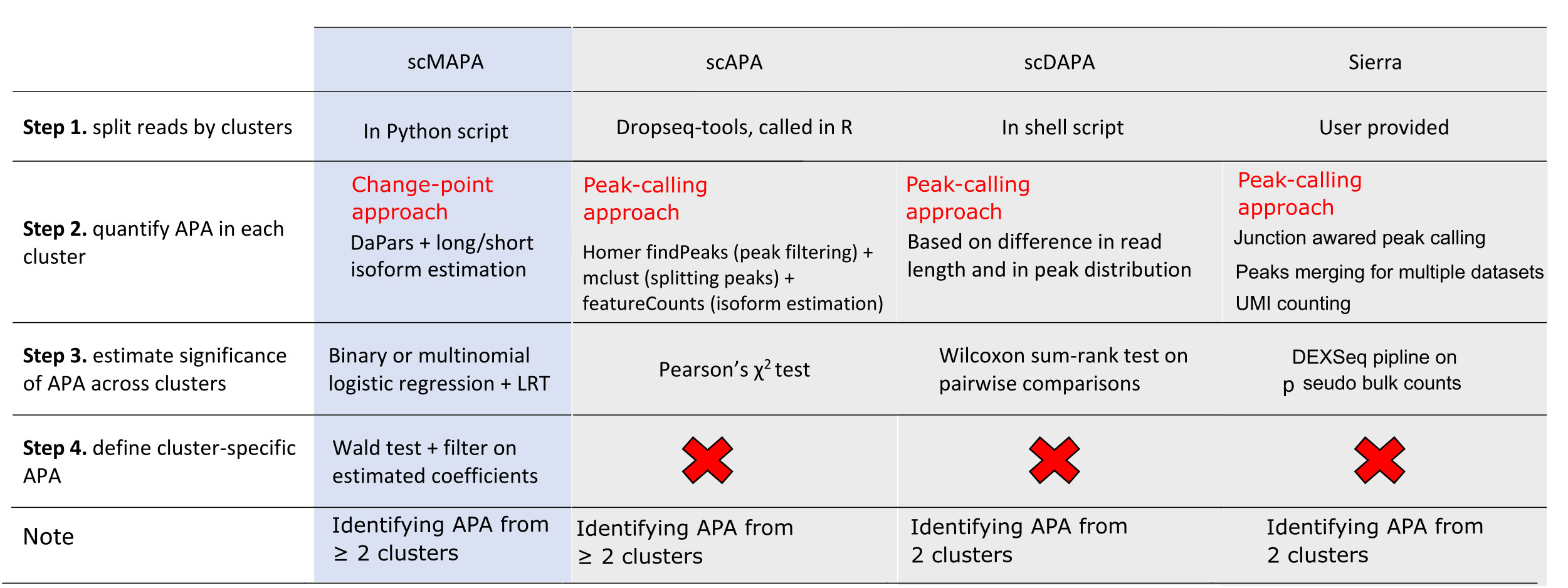


**Supplemental Figure 1.** Comparison of bioinformatic tools and statistical methods to identify dynamic APAs in scRNA-Seq data. Note that only scMAPA employs the change-point approach and can identify cell-type-specific APA genes.


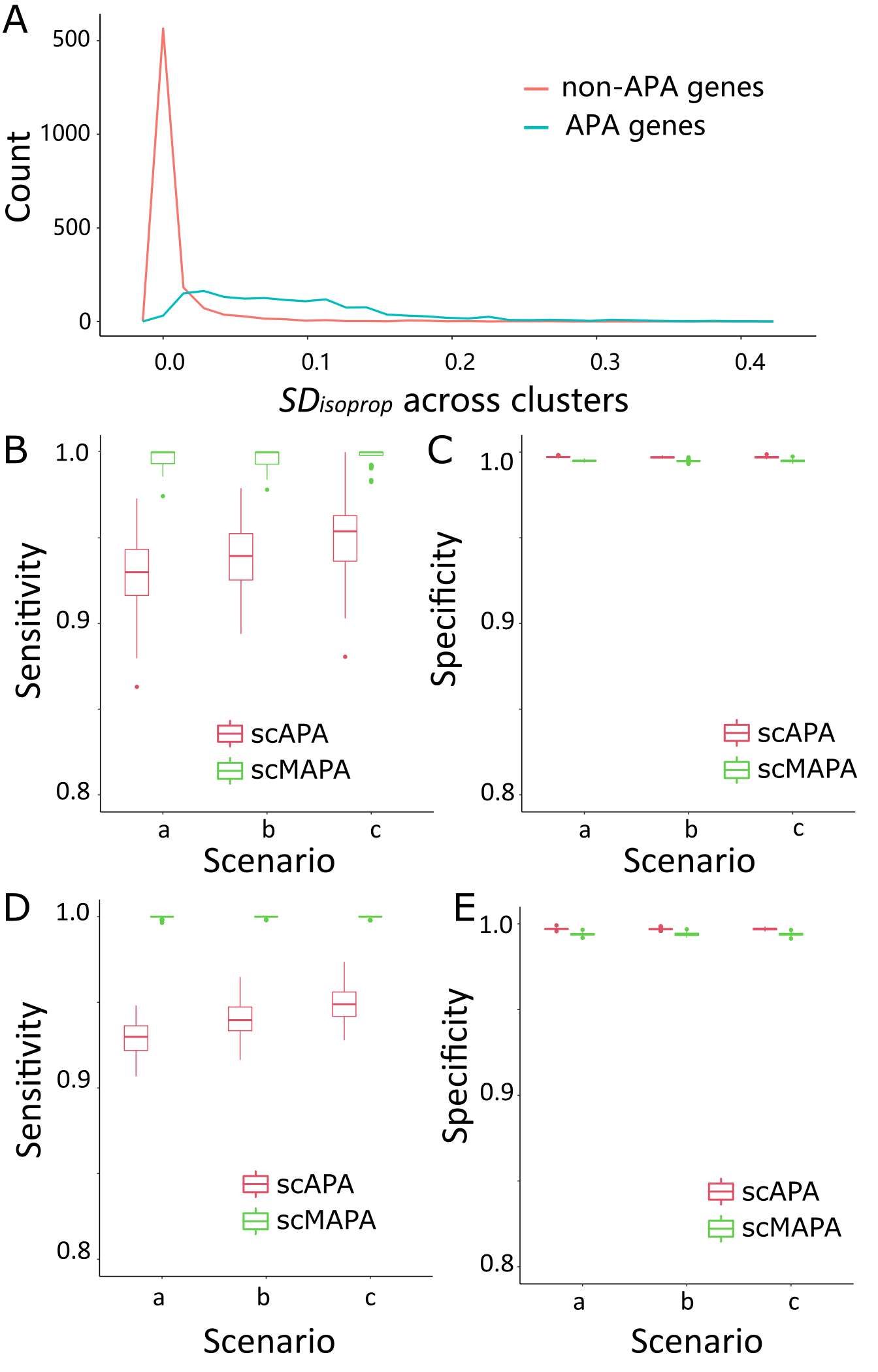


**Supplemental Figure 2**. Performance assessment on the statistical component of scMAPA (regression + LRT) and scAPA (Pearson’s χ^2^) using simulated data. (A) distribution of the standard deviation (SD) of PDUI values across clusters from mouse brain data. (B) to (E) show the performance assessment using simulated data. With fixed number of true APA events (250) and SD values (0.1268 for true APA genes and 0.009190 for non-APA genes), box plots in (B) and (C) show the sensitivity and specificity in scenarios with different distributions of cell type populations: (20%, 20%, 20%, 20%, 20%) for scenario a, (30%, 17.5%, 17.5%, 17.5%, 17.5%) for b, and (50%, 12.5%, 12.5%, 12.5%, 12.5%) for c. Box plots in (D) and (E) show the sensitivity and specificity with the number of true APA events set to 1,000 and all other factors remain same.


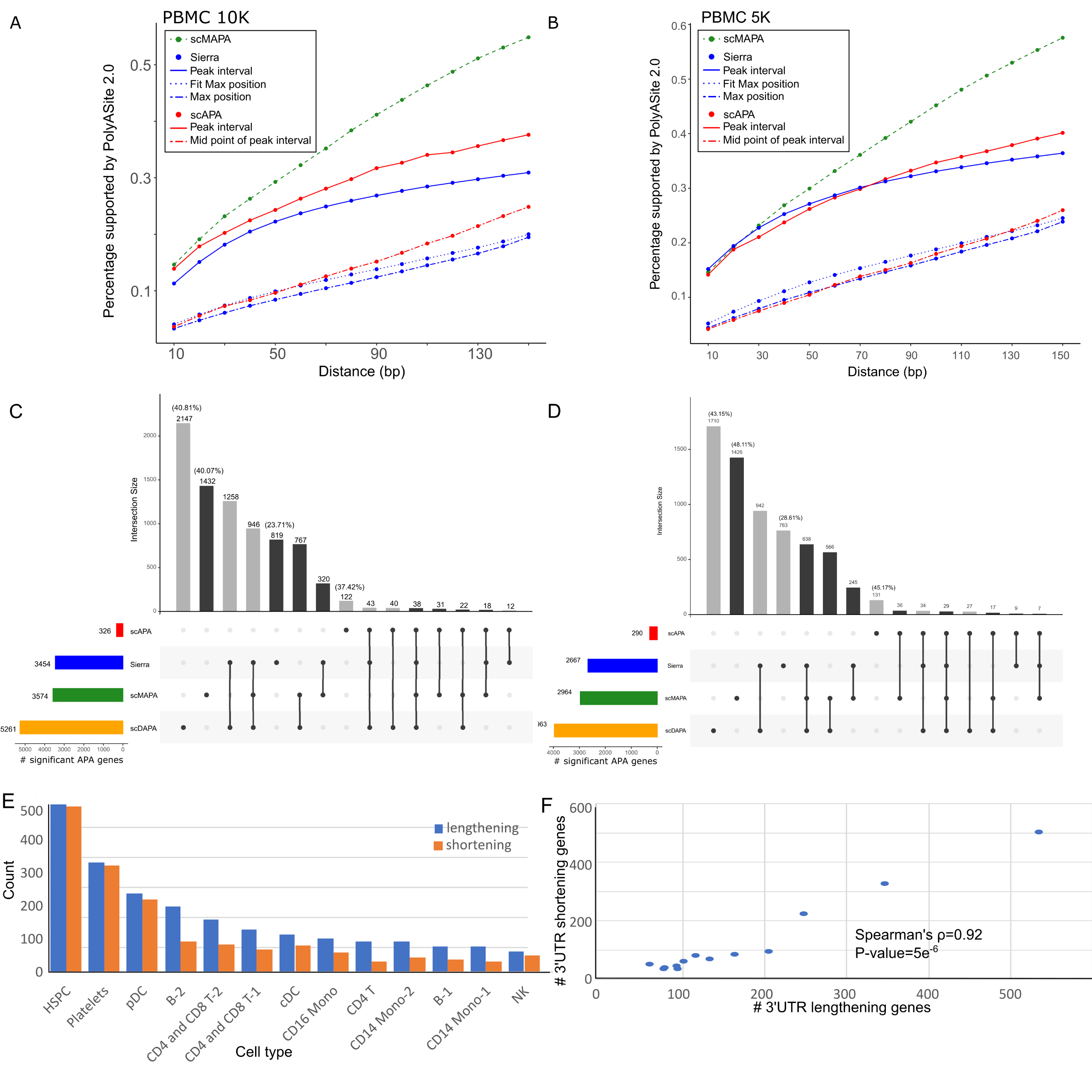


**Supplemental Figure 3.** Percentage of pA sites each method identified in the PBMC 10k (A) and 5k (B) data that are in proximity to known pA sites annotated in PolyASite 2.0 by the distance defining the proximity. Upset plot showing diverse overlaps among APA genes in the 10K (C) and 5k (D) data identified by four methods, scAPA, Sierra, scMAPA and scDAPA. Barplot on top shows the number of genes corresponding to the set combination indicated below. Black bars correspond to the sets involving scMAPA results. Colored horizontal bars represent the total number of APA genes identified by each method. (E) Barplot and (F) scatterplot of number of 3'UTR lengthening (blue) and shortening (red) genes in each of the PBMC cell types.


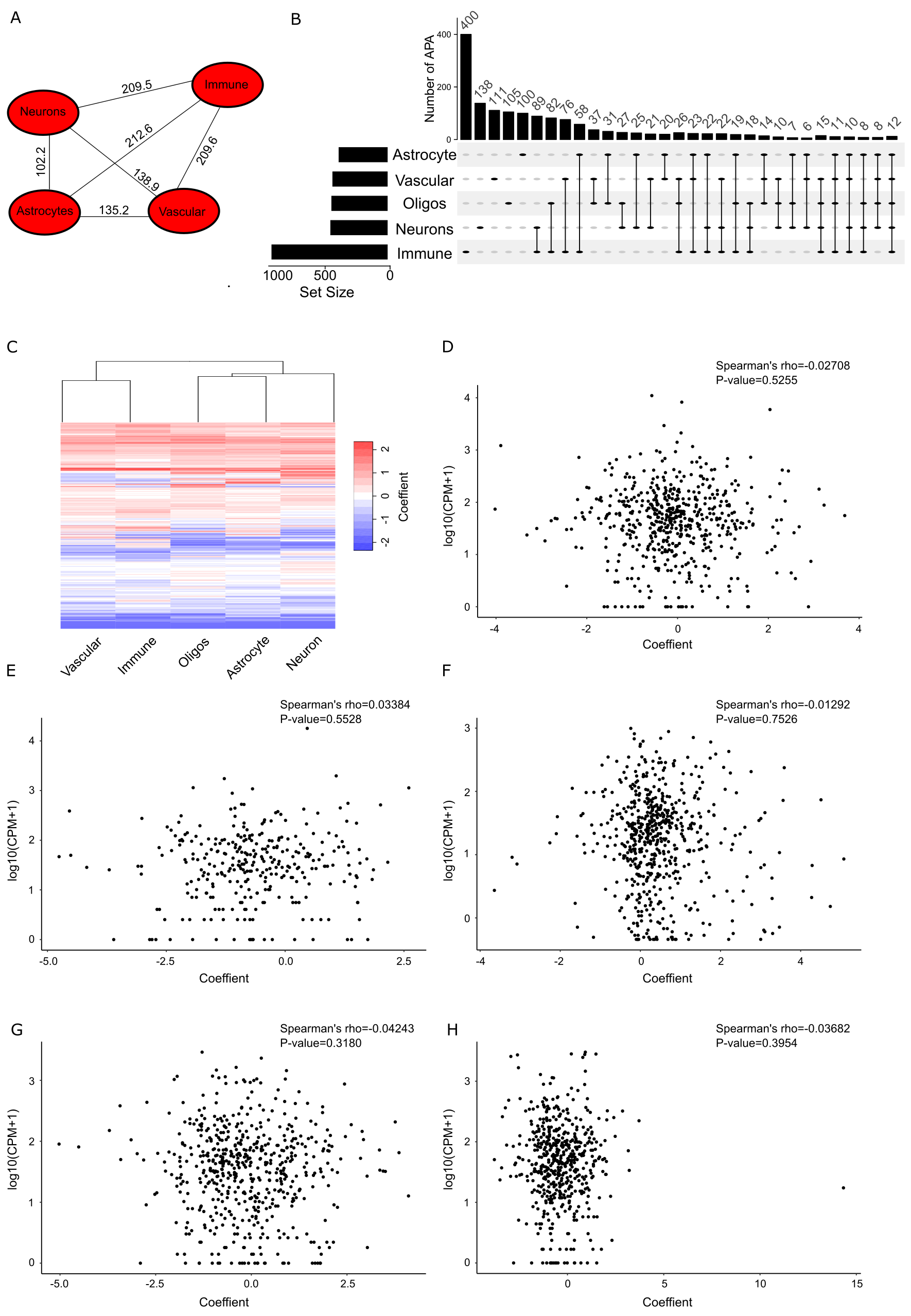


**Supplemental Figure 4.** (A) Euclidean distance between the cell types in the mouse brain data. This figure is not scaled according to the actual distance values. (B) Upset plot showing intersection of APA genes identified in each cell type. The bar in the upper panel indicates the size of the set described in the lower panel as it shows 5 types of cells and intersections across different combinations of cells. The bar on the left side shows the total number of APA genes specific to each cell type, including the intersection. (C) Heatmap of the effect sizes of expression level differences estimated for each cell type (D)-(H) Scatter plots show the correlation pattern between APA dynamic and expression of genes in Fig 4.C by cell type. X-axis represents coefficients shown in Fig 4.C, Y-axis represents log(CPM+1) shown in Fig S3.A. (D) shows the pattern for Astrocytes, (E) for Immune, (F) for Neurons, (G) for Oligos, (H) for Vascular cells.


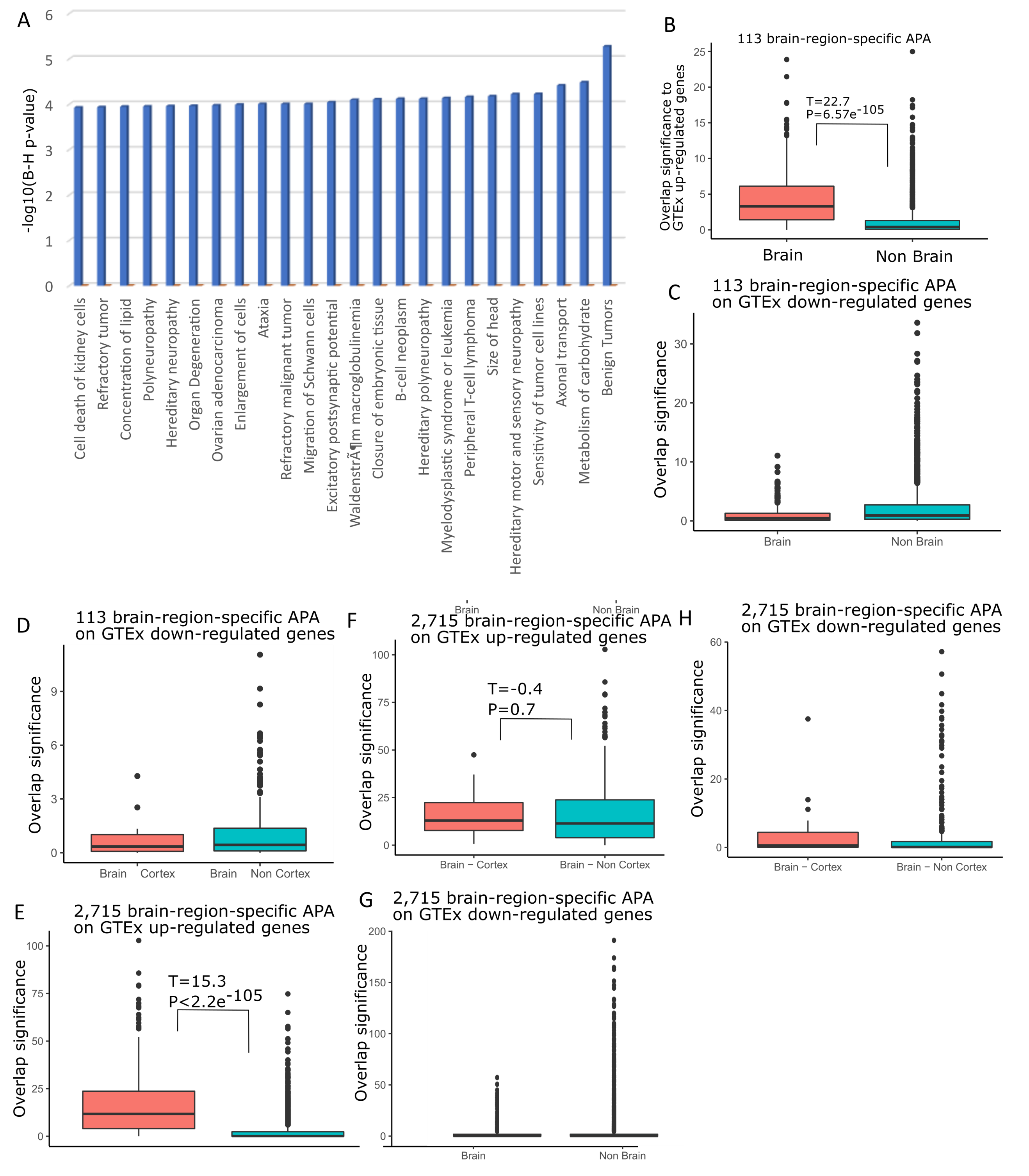


**Supplemental Figure 5.** (A) Significance (B-H p-value) of IPA enrichment of 2,715 confounder-unadjusted APA genes for the scMAPA. The -log(B-H p-value) of those terms using 2,715 confounder-adjusted APA genes are 0. The 24 terms are enriched only for the confounder-adjusted APA genes (Δlog(p-value) > 3 between confounder-adjusted vs. confounder-unadjusted). (B) Box plot showing significance of overlap between the 113 brain-region-specific APA genes and the up-regulated genes in GTEx samples whether they are from brain (red) or not (green). A higher overlap significance indicates a more enriched overlap, calculated by Enrichr. Significance of overlap between the 2,715 APA genes that are not specific to brain regions and the down-regulated genes in GTEx samples (C) whether they are from brain (red) or not (green) (D) whether they are from brain cortex region (red) or not (green). Significance of overlap between the 2,715 APA genes that are not specific to brain regions and the up-regulated genes in GTEx samples (E) whether they are from brain (red) or not (green) (F) whether they are from brain cortex region (red) or not (green). A higher overlap significance indicates a more significant overlap, calculated by Enrichr. Significance of overlap between the 2,715 APA genes that are not specific to brain regions and the down-regulated genes in GTEx samples (E) whether they are from brain (red) or not (green) (F) whether they are from brain cortex region (red) or not (green). A higher overlap significance indicates a more significant overlap, calculated by Enrichr.
