# Supplementary material for: scMAPA: Identification of cell-type–specific alternative polyadenylation in complex tissues
Source: Gigascience. 2022 Apr 30;11:giac033. doi: 10.1093/gigascience/giac033 (PMC9055853; doi:10.1093/gigascience/giac033)
Supplement: giac033_Response_to_Reviewer_Comments_Original_Submission [file giac033_response_to_reviewer_comments_original_submission.pdf]

Author's Response To Reviewer Comments

Close

RESPONSE TO REVIEWERS' CRITIQUES
Reviewer reports:
Reviewer #1: The authors have addressed some of my concerns but not others. The remaining major issue is that the definition of 'confounding factor' is quite confusing. The authors are recommended to provide a more intelligible description.

Response: We realize that the word "confounding" can mean differently by context, potentially confusing readers. Thus, we removed the word and rewrote those parts with the specific meaning of it, which is "undesired source of variation". To clarify further, we kept our focus on a specific case where brain region information becomes the undesired source of variation in identifying brain cell-type-specific APA genes throughout the manuscript.

Reviewer #2:
The authors present a novel tool scMAPA for the identification and quantification of alternative poly-adenylation sites from scRNA-seq.

The manuscript has had a substantial re-write and additional analyses performed since the previous submission. It has been improved significantly and previous comments addressed.

Response: Thank you so much for acknowledging our effort to improve our manuscript.

Major comments

In the Findings section there is too much methodology mentioned in it but without the detail so makes reading it harder. In the Findings section a focus should be on the results such as the comparison to Sierra and scAPA and what was identified in the PBMC and Mouse data.
Response: Thanks to this reviewer's comment, we moved the descriptions about methodology to Methods section. Previously, there were 2,689 words in Findings section. This will make our manuscript to be more understable. Thank you for your comment.

The authors need to also ensure the past tense is used consistently throughout. An example is:
p.13 para 2 line 14 "we test if they express highly"
is better as
"we tested if they were expressed highly"
Response: Thank you for your suggestion. We made this correction the reviewer suggested. Also, we made the following corrections we found in the same direction.
1. P.5 line 9, "this transformation made the APA short and long isoforms readily distinguishable" -> "this transformation makes the APA short and long isoforms readily distinguishable"
2. P.6 line 13, "which used the quadratic programming"-> "which uses the quadratic programming"
3. P.8 line 6, "the mouse brain data showed a narrow range"-> "the mouse brain data show a narrow range"
4. P.9 line 1, "scMAPA consistently outperformed the other methods"-> "scMAPA consistently outperforms the other methods"
5. P.9 line 13, "scMAPA identified a two-fold higher percentage of APA genes"-> "scMAPA identifies a two-fold higher percentage of APA genes"
6. P.10 line 17, "showed the dynamic APA isoform ratios across the cell types"-> "show the dynamic APA isoform ratios across the cell types"
7. P.11 line 4, "While both the analyses supported the previous finding" -> "While both the analyses support the previous finding"
8. P.11 line 15, "they further revealed that immune cells are most different from all the other cell types"-> "they further reveal that immune cells are most different from all the other cell types"
9. P.13 line 2, "APA genes associated with a brain region can be mistakenly identified as cell-type-

specific APA genes"-> "APA genes associated with a brain region could be mistakenly identified as cell-type-specific APA genes"
10. P.13 line 18, "GTEx does not collect the expression data for midbrain" -> "GTEx did not collect the expression data for midbrain"

On p. 9 para 1 line 4 different numbers of cells were defined: "6, 8, and 13 types for 1k, 5k, and 10k data respectively"
How representative are those numbers? There are 5x more cells in the 5k data than 1k yet only a third more cells types. The 5k vs 10k are more consistent: 2x cells and ~1.5x types.
Response: We determined the number of clusters by running a well-established algorithm. Especially, we used the parameters suggested particular for the 1k, 5k, and 10k data 1. To further validate the number of the clusters, we examined the percentage of variance explained (between-group variance/total variance) against the different number of clusters in elbow plot analysis (R. Fig. 1A, B, C for 1k, 5k, and 10k data respectively). From the elbow plots, we can see that the number of clusters was set in an acceptable range of the explained variance (between the steepest increase and the flattening point), suggesting that Seurat's method delineated an appropriate number of clusters in the 1k, 5k, and 10k data. Especially, although 5x more cells in the 5k data did not proportionally increase the number of clusters from the 1k data, the defined clusters explain a very similar percentage of the variance (~16.25%), supporting the number of clusters in the 1k and 5k data again.
Another support comes when checking the dimension-reduced space (UMAP) of the data (R. Fig. 1D, E, F for 1k, 5k, and 10k data respectively), since distinct cell types are expected to be well separated on the UMAP. Since it is the case for the 1k, 5k, 10k data, we believe that the numbers of the defined clusters were set appropriately.

p. 10 para 1 line 3. scMAPA found 40.7% genes as being APA compared to the other tools which found between 11.6-18.9%. Do the authors know whether that is a biological valid percentage?
Response: To identify a valid percentage in our experiment investigating how much APA genes are identified in different numbers of cells sampled, we ran scMAPA on the 1k and 10k data to find that 51.5% of the APA genes are common. Since the data were sampled from a same healthy donor, the number of APA genes common to the 1k and 10k data indicates the upper bound of the identification attributing mainly to the different number of cells in the data, and thus representing a biologically valid percentage for our experiment. Since 40.7% of genes commonly being APA is lower than this percentage, we believe that the results are valid, suggesting that scMAPA is most robust to the sample size changes.
Separately, to identify a biologically valid percentage in general, we searched literature reporting the number of APA events. Using a sequencing technique designed to comprehensively map polyadenylation sites, a recent study found that 78.5% of mRNA genes were found to undergo APA events on the RNA samples across a more diverse set of samples ((i) male and female whole bodies; (ii) embryos at 11, 15 and 17 days (d); (iii) brain and testis tissues at different postnatal stages; and (iv) over 11 cell lines2).

p. 10 para 2 line 4: "enrichments to 32 IPA terms that are characterized with keywords "blood" and "hematology", suggesting that the APA genes identified by scMAPA can play important roles in PBMC biology".
The PBMC dataset is a blood cell dataset which one would be always enriched with the terms "blood" and "hematology". Is that not so? How is the IPA returning enrichment for these terms a measure of scMAPA accuracy?

The reviewer is right that any functional component in the blood cells is expected to be enriched with keywords "blood" and "hematology". Based on this rationale, we wanted to see if the identified APA genes implicate any functions in the blood cells by inspecting their enrichment terms. To conduct this analysis stringently and reduce the chance of random enrichment for the keywords, we "set the 18,804 genes expressed in the data as the background" in the analysis, so, for the APA genes to be significantly enriched with the "blood" and "hematology" terms, the enrichment degree should exceed what could be expected generally from the expressed genes in the PBMC data.
Also, the reviewer is right that these enrichments couldn't be a measure of scMAPA accuracy. That is why we did not claim scMAPA accuracy with this finding, but to suggest "that the APA genes identified by scMAPA can play important roles in PBMC biology".

p. 11 para 1 line 9: "Since bone marrow is developmentally related to peripheral blood, GATA2 may undergo the APA event in the PBMC under similar molecular mechanisms."
This statement needs to be supported with further evidence or the authors should say this is speculation.

Response: We changed the text in the manuscript as follows. “Since hematopoietic stem and progenitor cells (HSPC in Fig. 1C, D) are originated from bone marrow3, we speculate that the molecular mechanisms rendering the APA event on GATA2 in the bone marrow mononuclear cells cause GATA2 to show different APA patterns than other cells in the PBMC.”

Minor comments

Abstract: para 2, line 3: "To release the assumptions" should be "To avoid the assumptions"
Response: This has been changed according to your suggestion.

p-values reported in scientific notation should be in the form 2.2 x 10^-16 not 2.2e^-16 as reported on p. 7 para 2 line 15. Also 10^-2 (p. 10 para 2 line 5) is better as 0.01
Response: We changed “p<2.2e^-16” into “p-value<2.2×10-16”. Also, we changed “B-H p<10^-2” to “0.01”. Further, para 3 had two occurrences of “B-H P-val < 0.05”. We changed them into “B-H p-val < 0.05” to make the same style. Several other places had similar issues. We changed them into the same style.

p. 10 para 2 line 4: spell out acronyms the first time they're used: B-H as Benjamini-Hochberg
Response: This has been changed according to your suggestion.

p. 11 para 1 line 10: "biologically reasonable APA genes" should be "biologically relevant APA genes"
Response: This has been changed according to your suggestion.

p. 14 para 2 line 9: is "10^3.5" what is meant here? Re-write in proper scientific notation as mentioned above.
Response: We changed it into “p-value < 2.2×10-4”.

p. 22 para 1 line 6: "at least 20 raw counts" of what?
Response: It is “at least 20 raw counts of reads”. With this change, we updated that part as follows. “In addition to gene-wise filtering, we also apply cell-wise filtering for each passed gene to keep only cell types with at least 20 raw counts of reads in the model. For each gene, cell types with extremely low coverage (< 20) will not be used to estimate the APA status.”

p. 24 para 2 line 4: "than ln(2), corresponding to a 2-fold change in odds ratio" is incorrect as ln() is the natural log so ln(2) equals 0.693
Response: We used logistic regression where ln(2) corresponds to a 2-fold change in odds ratio. However, we understand that this might confuse readers. So, we changed the text as follows. “we further selected genes whose APA degrees change greater than 2-fold. If the APA degree increases greater than 2-fold, the respective gene is considered as 3'-UTR lengthening. And, if the APA degree decreases less than 2-fold, the respective gene is considered as 3'-UTR shortening. However, users can define a different cutoff value of fold change to call 3'-UTR lengthening or shortening.”

p. 24 para 3 line 1: "scMAPA can be easily extended" is better as "scMAPA has been extended".
Response: Although we decided not to do the extension in this manuscript for fair comparisons with other methods and for effective investigation of multiple cell types (see 3rd para in Discussion), we agree with the reviewer that this extension will be helpful for further analyses. We will work on this extension as future work. We made this point more specific in the 3rd para in Discussion.

Close
