# Supplementary material for: scMAPA: Identification of cell-type–specific alternative polyadenylation in complex tissues
Source: Gigascience. 2022 Apr 30;11:giac033. doi: 10.1093/gigascience/giac033 (PMC9055853; doi:10.1093/gigascience/giac033)
Supplement: giac033_Response_to_Reviewer_Comments_Revision_1 [file giac033_response_to_reviewer_comments_revision_1.pdf]

Author's Response To Reviewer Comments

Close

This is in response to the Editor's request to upload a word version of our manuscript. In the manuscript, we added the citation to GigaDB as requested.

Close
