# Supplementary material for: scMAPA: Identification of cell-type–specific alternative polyadenylation in complex tissues
Source: Gigascience. 2022 Apr 30;11:giac033. doi: 10.1093/gigascience/giac033 (PMC9055853; doi:10.1093/gigascience/giac033)
Supplement: giac033_Reviewer_2_Report_Original_Submission — Christian Cole -- 10/3/2021 Reviewed [file giac033_reviewer_2_report_original_submission.pdf]

Reviewer Report

Title: scMAPA: Identification of Cell-type-specific Alternative Polyadenylation in Complex Tissues

Version: Original Submission **Date:** 10/3/2021

Reviewer name: Christian Cole

Reviewer Comments to Author:

The authors present a novel tool scMAPA for the identification and quantification of alternative poly-adenylation sites from scRNA-seq.
The manuscript has had a substantial re-write and additional analyses performed since the previous submission. It has been improved significantly and previous comments addressed.
Major comments
In the Findings section there is too much methodology mentioned in it but without the detail so makes reading it harder. In the Findings section a focus should be on the results such as the comparison to Sierra and scAPA and what was identified in the PBMC and Mouse data.
The authors need to also ensure the past tense is used consistently throughout. An example is:
p.13 para 2 line 14 "we test if they express highly"
is better as
"we tested if they were expressed highly"
On p. 9 para 1 line 4 different numbers of cells were defined: "6, 8, and 13 types for 1k, 5k, and 10k data respectively"
How representative are those numbers? There are 5x more cells in the 5k data than 1k yet only a third more cells types. The 5k vs 10k are more consistent: 2x cells and ~1.5x types.
p. 10 para 1 line 3. scMAPA found 40.7% genes as being APA compared to the other tools which found between 11.6-18.9%. Do the authors know whether that is a biological valid percentage?
p. 10 para 2 line 4: "enrichments to 32 IPA terms that are characterized with keywords "blood" and "hematology", suggesting that the APA genes identified by scMAPA can play important roles in PBMC biology".
The PBMC dataset is a blood cell dataset which one would be always enriched with the terms "blood" and "hematology". Is that not so? How is the IPA returning enrichment for these terms a measure of scMAPA accuracy?
p. 11 para 1 line 9: "Since bone marrow is developmentally related to peripheral blood, GATA2 may undergo the APA event in the PBMC under similar molecular mechanisms."
This statement needs to be supported with further evidence or the authors should say this is speculation.
Minor comments
Abstract: para 2, line 3: "To release the assumptions" should be "To avoid the assumptions"

p-values reported in scientific notation should be in the form 2.2 x 10^-16 not 2.2e^-16 as reported on p. 7 para 2 line 15. Also 10^-2 (p. 10 para 2 line 5) is better as 0.01
p. 10 para 2 line 4: spell out acronyms the first time they're used: B-H as Benjamini-Hochberg
p. 11 para 1 line 10: "biologically reasonable APA genes" should be "biologically relevant APA genes"
p. 14 para 2 line 9: is "10^3.5" what is meant here? Re-write in proper scientific notation as mentioned above.
p. 22 para 1 line 6: "at least 20 raw counts" of what?
p. 24 para 2 line 4: "than ln(2), corresponding to a 2-fold change in odds ratio" is incorrect as ln() is the natural log so ln(2) equals 0.693
p. 24 para 3 line 1: "scMAPA can be easily extended" is better as "scMAPA has been extended".

Level of Interest

Please indicate how interesting you found the manuscript: Choose an item.

Quality of Written English

Please indicate the quality of language in the manuscript: Choose an item.

Declaration of Competing Interests

Please complete a declaration of competing interests, considering the following questions:

- Have you in the past five years received reimbursements, fees, funding, or salary from an organisation that may in any way gain or lose financially from the publication of this manuscript, either now or in the future?
- Do you hold any stocks or shares in an organisation that may in any way gain or lose financially from the publication of this manuscript, either now or in the future?
- Do you hold or are you currently applying for any patents relating to the content of the manuscript?
- Have you received reimbursements, fees, funding, or salary from an organization that holds or has applied for patents relating to the content of the manuscript?
- Do you have any other financial competing interests?
- Do you have any non-financial competing interests in relation to this paper?

If you can answer no to all of the above, write 'I declare that I have no competing interests' below. If your reply is yes to any, please give details below.

I declare that I have no competing interests

I agree to the open peer review policy of the journal. I understand that my name will be included on my report to the authors and, if the manuscript is accepted for publication, my named report including any attachments I upload will be posted on the website along with the authors' responses. I agree for my report to be made available under an Open Access Creative Commons CC-BY license (http://creativecommons.org/licenses/by/4.0/). I understand that any comments which I do not wish to be included in my named report can be included as confidential comments to the editors, which will not be published.

Choose an item.

To further support our reviewers, we have joined with Publons, where you can gain additional credit to further highlight your hard work (see: https://publons.com/journal/530/gigascience). On publication of this paper, your review will be automatically added to Publons, you can then choose whether or not to claim your Publons credit. I understand this statement.

Yes Choose an item.
